# Supplementary material for: Concerted dynamics of metallo-base pairs in an A/B-form helical transition
Source: Nat Commun. 2019 Oct 23;10:4818. doi: 10.1038/s41467-019-12440-x (PMC6811676; doi:10.1038/s41467-019-12440-x)
Supplement: Supplementary file 1 — Supplementary Information [file 41467_2019_12440_MOESM1_ESM.pdf]

SUPPLEMENTARY INFORMATION

Concerted dynamics of metallo-base pairs in an A/B-form helical transition

Schmidt et al.

Table of Contents

Supplementary Figures **3**

Supplementary Tables **45**

Supplementary Methods **51**

Synthesis and Folding of Duplex DNA **51**
Melting Temperature Analysis (T_m) and CD Spectroscopy **51**
Sample Preparation of Duplex DNA used for NMR studies **52**
NMR Spectra Measurements **52**
NMR Solution Structure Calculations **54**
HR-MS of $Hg(ClO_4)_2$ **55**
Calculation of Pseudorotation Phase Angle **55**
Rate Constants of Nucleobase-Metal-Nucleobase Isomerization **56**
Variable Ionic Strength **57**
Variable pH **58**
Rate Constants of Global Helix Interconversion **58**
Hg^{II}-Induced Conformational Switching from B- to A-Form **61**
Gel Electrophoreses **61**
Neo-BODIPY Binding Assay **61**
A → B Helical Switching by Unlabeled Neomycin B **62**
A → B Helical Switching by *N*-Acetylcysteine **62**
Circular Dichroism Spectroscopy of DNA Repeat Sequences **62**
Hg^{II}–ODN^{13} Binding and Reversibility by *N*-Acetylcysteine **63**
Hg^{II}–ODN^{13} ds Binding and Reversibility by *N*-Acetylcysteine **63**

Supplementary References **63**

Supplementary Figures

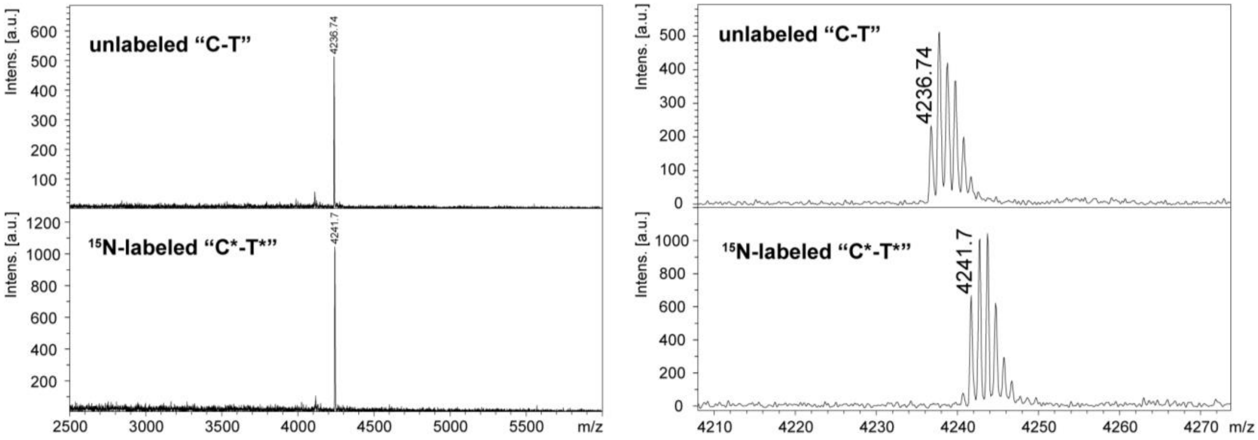

Supplementary Figure 1 | MALDI-MS analysis of unlabeled ODN[1] "C-T" and ^{15}N-labeled ODN[1]* "C*-T*". ^{15}N-labeled "C*-T*" showed a mass elevation of 5 dalton.

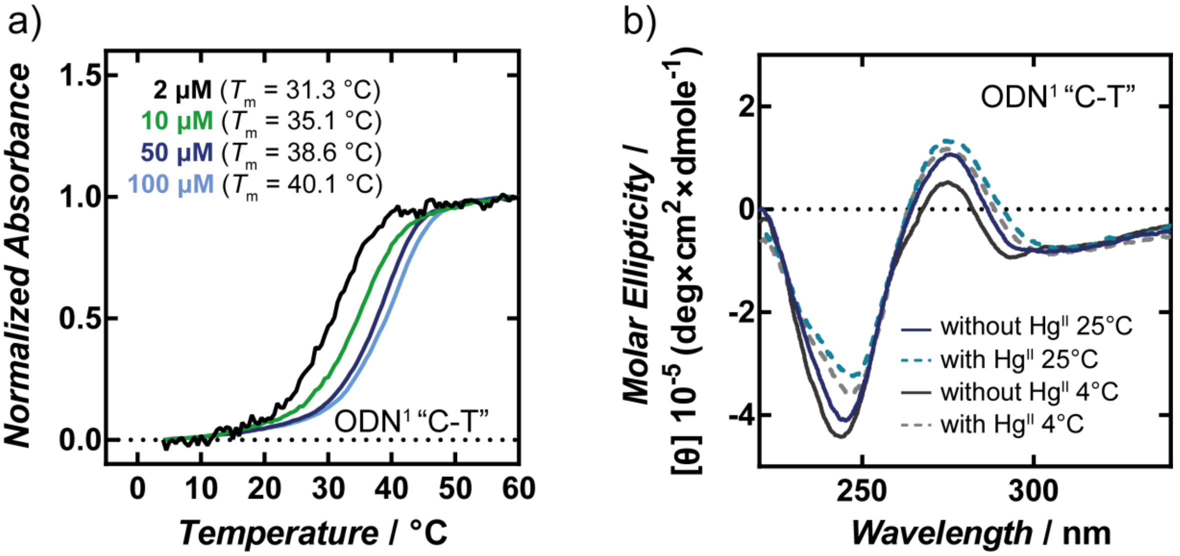

Supplementary Figure 2 | Melting temperature analysis (T_m) and circular dichroism spectroscopy (CD). a) Thermal melting of ODN[1] "C-T" at variable DNA concentrations.[1] b) CD spectra of ODN[1] "C-T" in the presence and absence of Hg^{II} at 4 °C and 25 °C.[1] Parts of the figure were taken from ref 1. For duplex sequence see Supplementary Table 1.

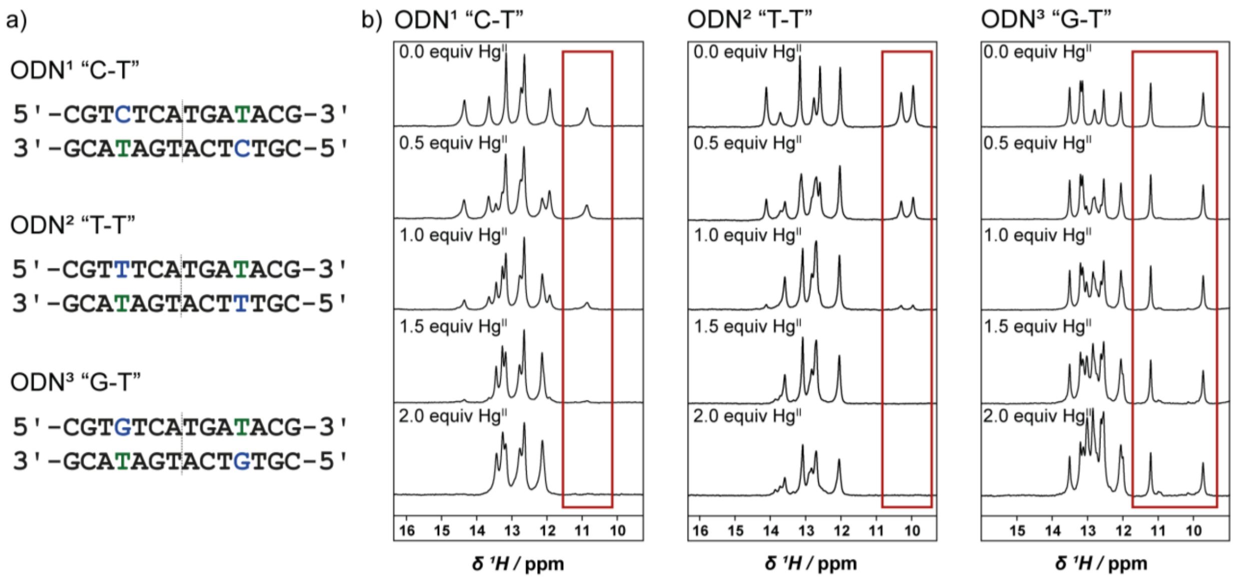

Supplementary Figure 3 | C-Hg^{II}-T base pair formation according to changes in ^{1}H NMR spectra upon addition of Hg^{II}. a) Names and sequences of palindromic, 14-mer duplex DNA "C-T" and "T-T", and "G-T". b) Disappearance of mismatched thymidine imino proton resonances upon addition of Hg^{II} to C-T- and T-T mismatch containing duplex DNA revealed stoichiometric binding of Hg^{II} to C-T- and T-T-mismatches. Addition of Hg^{II} to an analogous duplex containing G-T wobble base pairs caused no such deprotonation. All samples contained 0.5 mM of duplex DNA in aqueous buffer (200 mM $NaClO_4$, 50 mM cacodylic acid in H_2O / D_2O (9:1) at pH = 7.0). DNA samples were incubated with variable concentrations of $Hg(ClO_4)_2$ at r.t. for 15 min, followed by cooling at 4 °C for 15 min. Equiv of Hg^{II} given relative to mismatch.

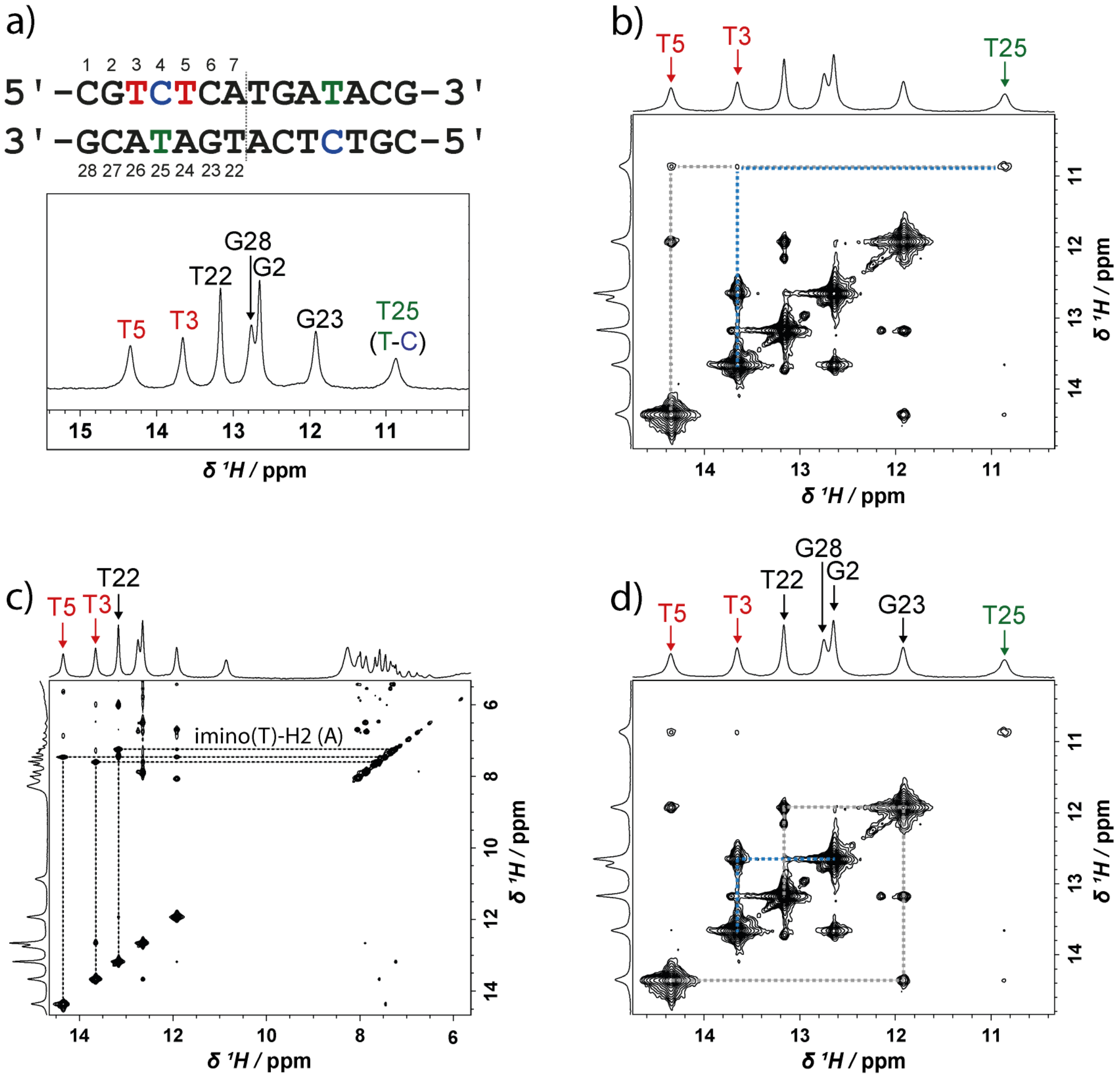

Supplementary Figure 4 | Assignment of *NH* imino protons of the duplex ODN[1] "C-T" in the absence of Hg^{II}. a) "C-T" sequence and imino region of ^1H NMR spectrum. Blue and green bases indicate C-T mismatches and red bases indicate residues flanking C-T mismatches. b) Assignment of thymidine residues flanking (red) the C-T mismatch (blue and green) according to NOE cross peaks between *NH* imino protons of thymidine. c) Assignment of thymidine imino protons according to NOE cross peaks between *NH* imino signals of thymidine residues and H2 of adenine residues. d) Assignment of guanosine residues of "C-T" according to NOE cross peaks between *NH* imino protons. The DNA sample contained 0.5 mM duplex DNA in aqueous buffer (200 mM $NaClO_4$, 50 mM cacodylic acid in H_2O / D_2O (9:1) at pH = 7.0). Spectra were recorded at 4 °C.

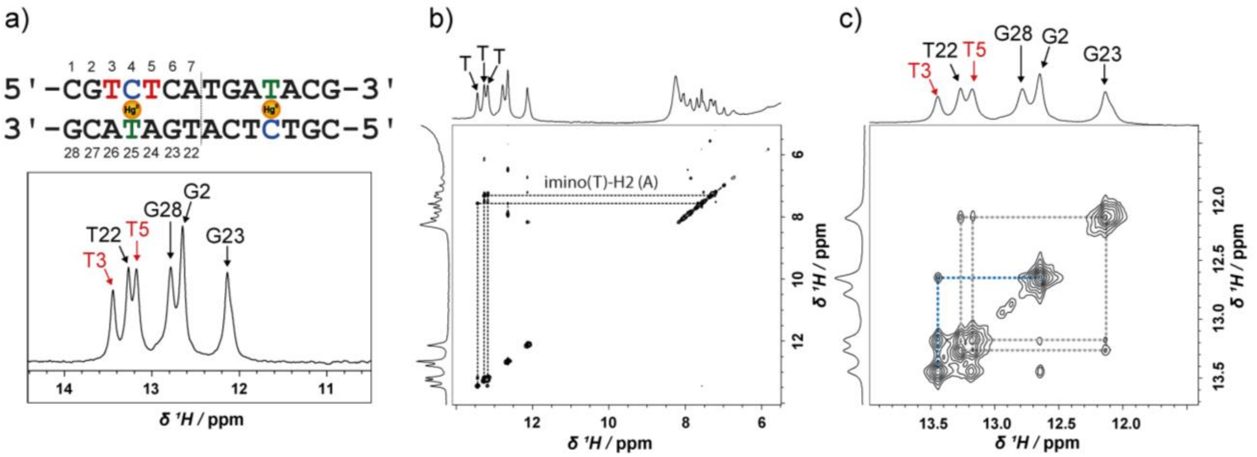

Supplementary Figure 5 | Assignment of *NH* imino protons of ODN[1] "C-T" in the presence of 1.5 equiv Hg^{II}. a) "C-T" sequence and imino region of ^{1}H NMR spectrum. Blue and green bases indicate C-T mismatches and red bases indicate residues flanking C-T mismatches. b) Assignment of thymidine imino protons according to NOE cross peaks between *NH* imino signals of thymidine residues and H2 of adenine residues. c) Assignment of thymidine and guanosine imino proton signals in the presence of Hg^{II} according to NOE cross peaks between *NH* imino protons. The DNA sample contained 0.5 mM duplex DNA and 1.5 mM Hg^{II} (1.5 equiv relative to mismatch) in aqueous buffer (200 mM $NaClO_4$, 50 mM cacodylic acid in H_2O / D_2O (9:1) at pH = 7.0). Spectra were recorded at 4 °C. Equiv of Hg^{II} given relative to mismatch.

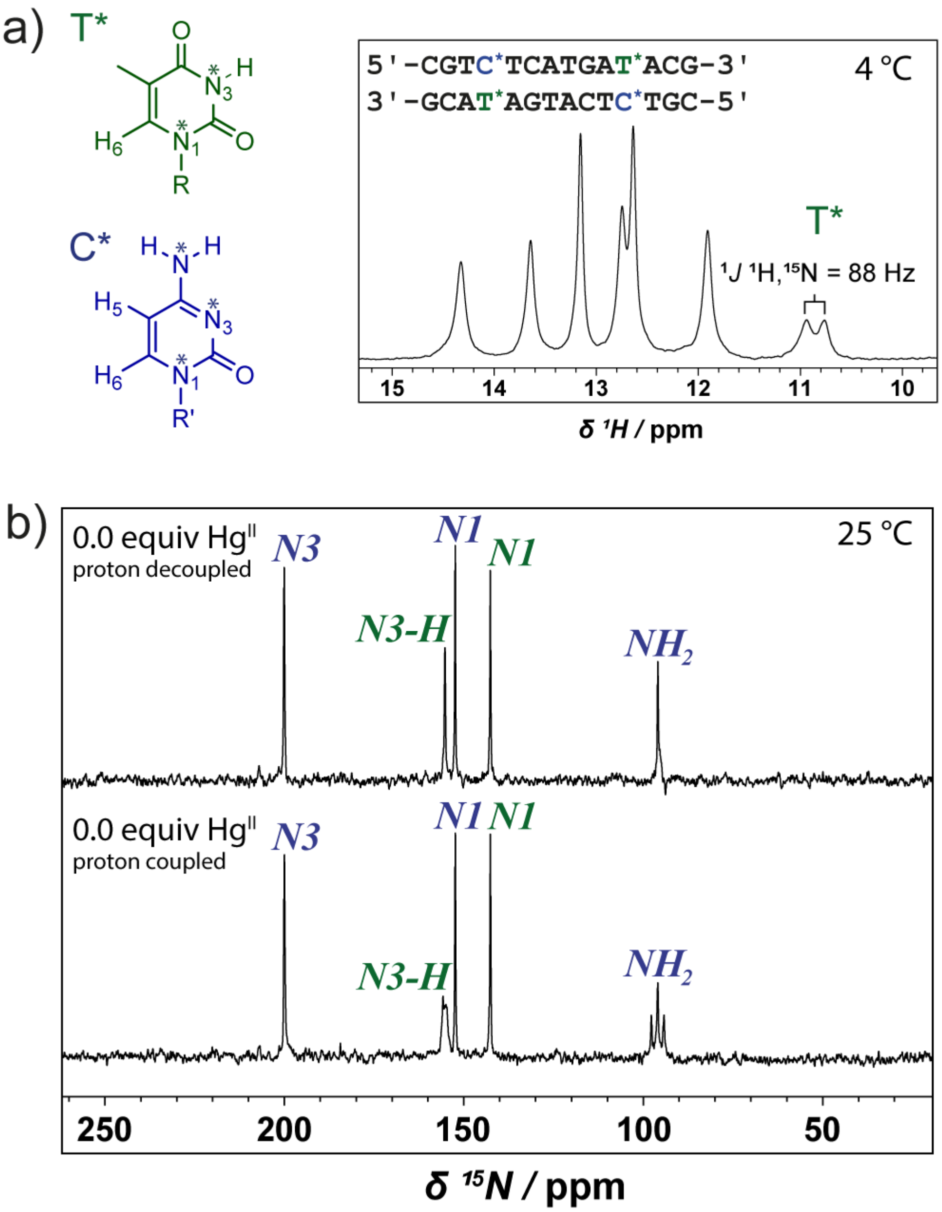

Supplementary Figure 6 | ^{1}H- and ^{15}N NMR spectra of ODN[1] "C*-T*" in the absence of Hg^{II}. a) ^1J ^{1}H,^{15}N coupling (^1J = 88 Hz) of N3-H imino proton of mismatched thymidine according to ^{1}H NMR spectrum of ODN^{1*} "C*-T*" containing a site-selective, ^{15}N-labeled C-T mismatch at 4 °C (N* = ^{15}N). b) Assigned proton decoupled- and proton coupled ^{15}N NMR spectra of ODN^{1*} "C*-T*" at 25 °C. Blue indicates ^{15}N resonances of C* and green indicates ^{15}N resonances of T*. DNA samples contained 0.5 mM (^{1}H) and 1 mM (^{15}N) duplex DNA in aqueous buffer (200 mM $NaClO_4$, 50 mM cacodylic acid in H_2O / D_2O (9:1) at pH = 7.8).

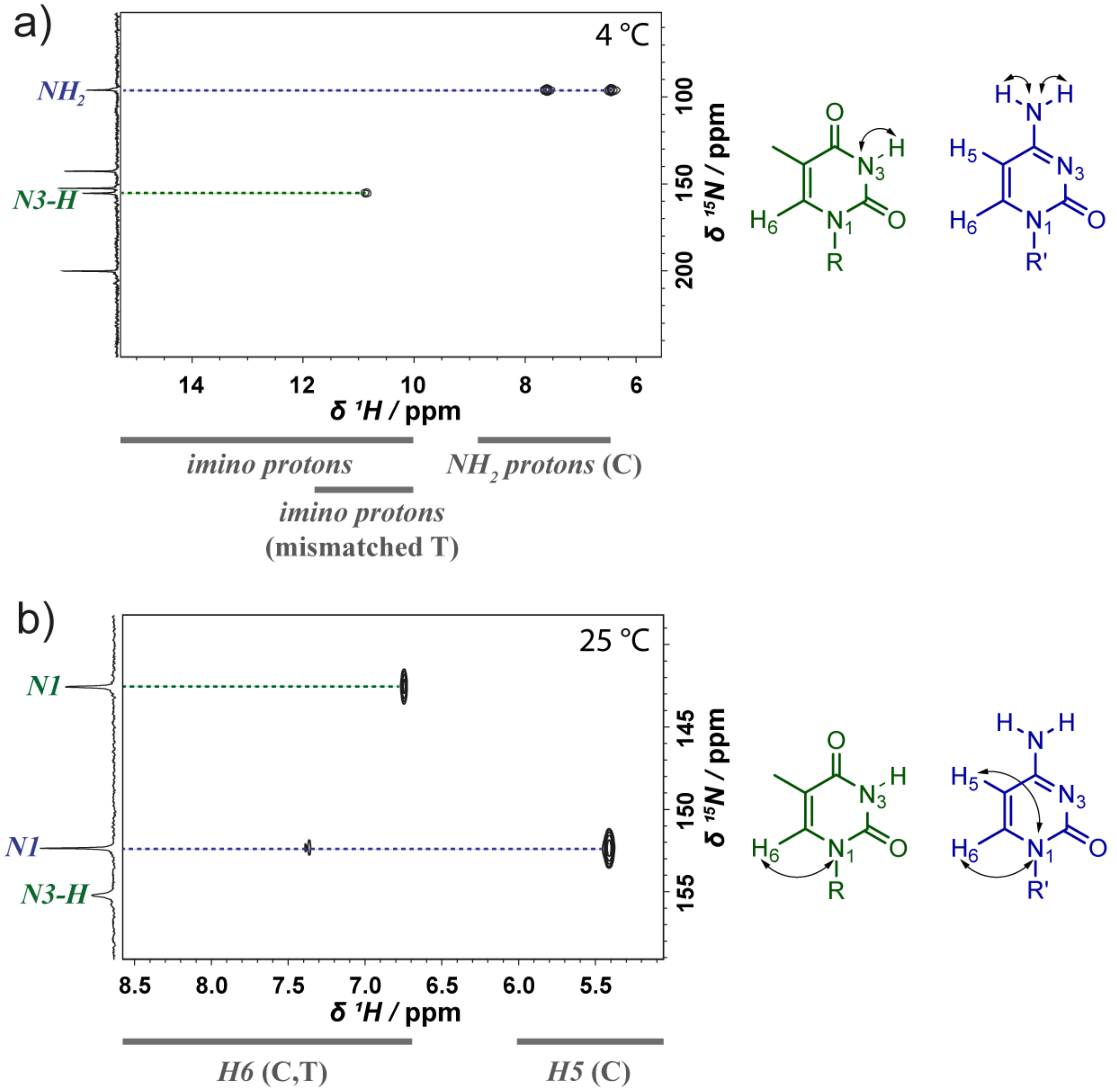

Supplementary Figure 7 | ^{15}N resonance assignment of ODN^{1} "C*-T*" in the absence of $\mathrm{Hg}^{\mathrm{II}}$. a) ^{1}J ^{1}H,^{15}N coupling of thymidine N3-H (green) and ^{1}J ^{1}H,^{15}N coupling of cytosine exocyclic amine (blue) according to [^{15}N,^{1}H]-HSQC spectrum at 4 °C. b) ^{2}J ^{1}H,^{15}N coupling between thymidine N1 and H6 (green) and ^{3}J- and ^{2}J ^{1}H,^{15}N coupling between cytosine N1 and H5 and H6 (blue) observed in the long-range [^{15}N,^{1}H]-HSQC spectrum at 25 °C. DNA samples contained 1.0 mM duplex DNA (ODN^{1}* "C*-T*") in aqueous buffer (200 mM $NaClO_4$, 50 mM cacodylic acid in H_2O / D_2O (9:1) at pH = 7.8) and were measured at the indicated temperature.

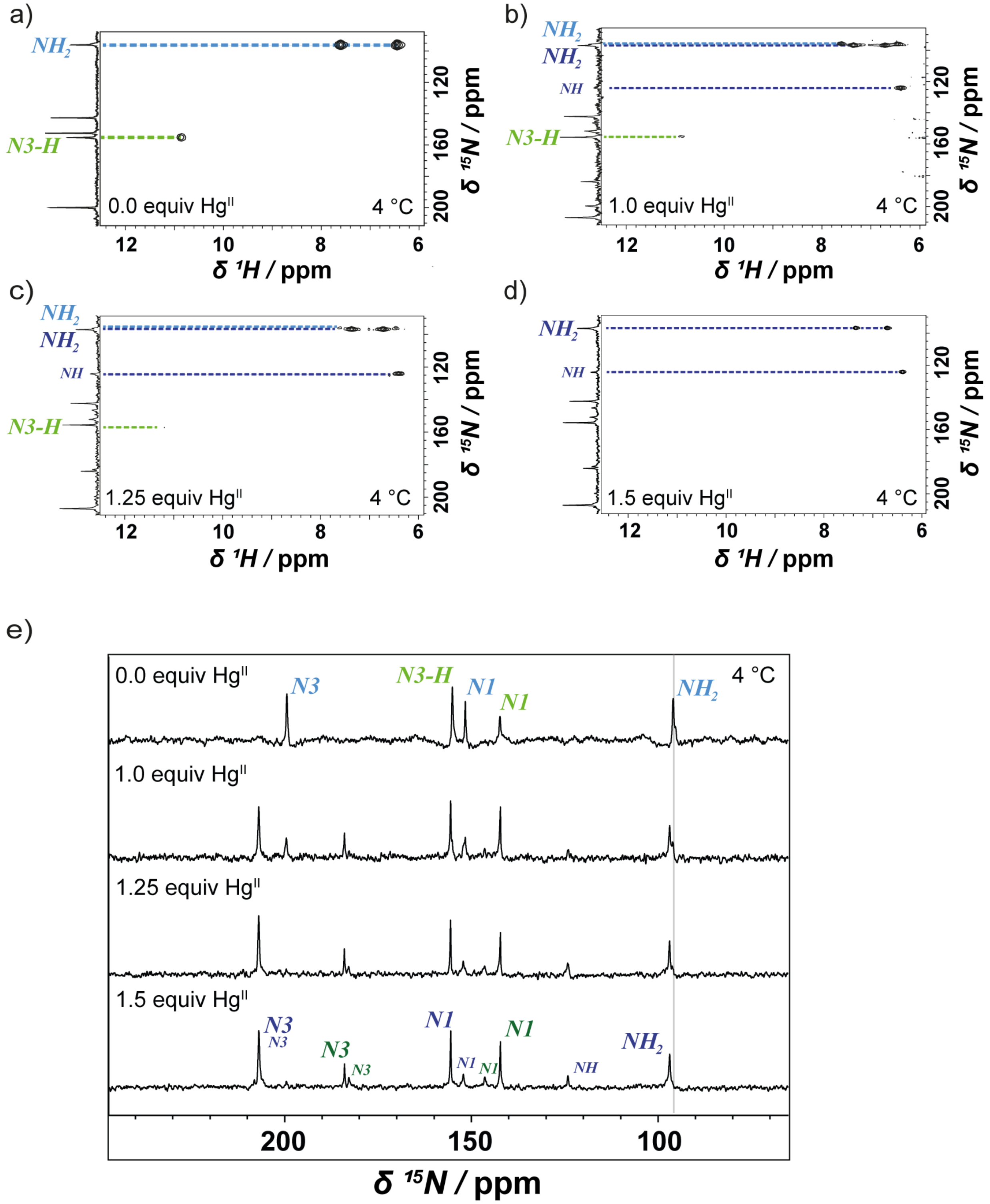

Supplementary Figure 8 | Changes in [^{15}N,^{1}H]-HSQC spectra and 1D ^{15}N spectra upon addition of Hg^{II} to ODN^{1*} "C*-T*". [^{15}N,^{1}H]-HSQC spectra (a – d) and e) 1D ^{15}N spectra. 1.5 equiv of Hg^{II} were needed to saturate C-T mismatch-containing duplex DNA and induce complete disappearance of ^{15}N signals of unbound duplex (Hg^{II}-unbound signals: light blue and light green; Hg^{II}-bound signals: dark blue, dark green). DNA samples contained 0.5 mM duplex DNA (ODN^{1*} "C*-T*") in aqueous buffer (200 mM $NaClO_4$, 50 mM cacodylic acid in H_2O / D_2O (9:1) at pH = 7.8). Spectra were recorded at 4 °C. Equiv of Hg^{II} given relative to mismatch.

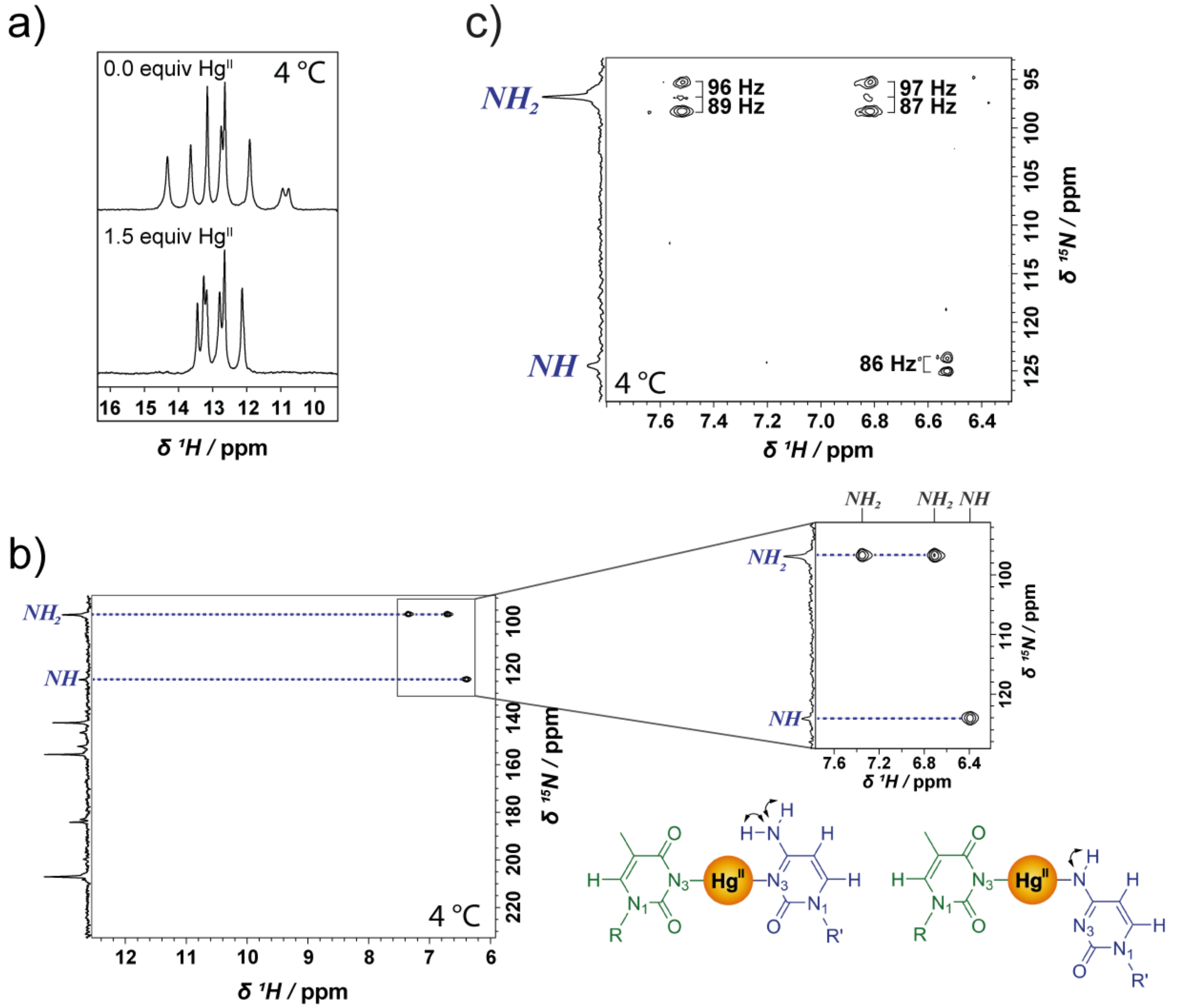

Supplementary Figure 9 | ^{1}H- and [^{15}N,^{1}H]-HSQC spectrum of ODN^{1*} "C*-T*" in the presence of Hg^{II}. a) Deprotonation of mismatched thymidine N3-H upon addition of 1.5 equiv of Hg^{II}. b) ^{1}J ^{1}H,^{15}N coupling of thymidine (N4)NH$_2^{major}$ and (N4)NH^{minor} of cytosine according to [^{15}N,^{1}H]-HSQC spectrum. c) Doublet (124 ppm, ^{1}J = 86 Hz) observed in the proton-coupled [^{15}N,^{1}H]-HSQC spectrum confirmed deprotonation of exocyclic amine in the minor binding mode. Blue indicates ^{15}N resonances of C*. DNA samples contained 0.5 mM duplex DNA (ODN^{1*} "C*-T*") and 1.5 mM Hg^{II} (1.5 equiv relative to mismatch) in aqueous buffer (200 mM NaClO$_4$, 50 mM cacodylic acid in H_2O / D_2O (9:1) at pH = 7.8) and were measured at the indicated temperature.

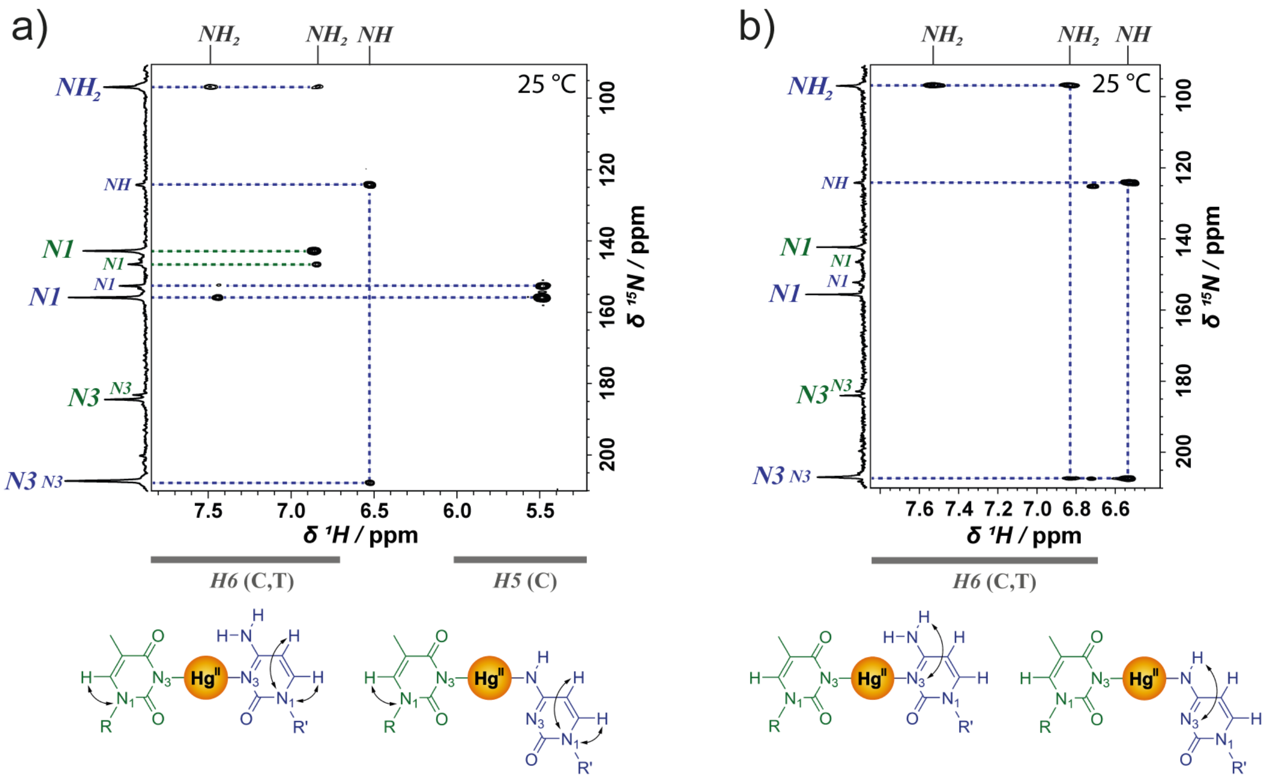

Supplementary Figure 10 | ^{15}N resonance assignment of major- and minor C-Hg^{II}-T coordination mode. a) Assignment of N1^{major} and N1^{minor} of thymidine and cytosine by ^{3}J- and ^{2}J ^{1}H,^{15}N coupling between N1 and H5 and/or H6 in the long-range [^{15}N,^{1}H]-HSQC spectrum. b) Assignment of N3^{major} and N3^{minor} of cytosine according to ^{3}J ^{1}H,^{15}N coupling to (N4)NH$_2^{major}$ and (N4)NH^{minor} in the band-selective, long-range [^{15}N,^{1}H]-HSQC spectrum at 25 °C. Blue indicates ^{15}N resonances of C* and green indicates ^{15}N resonances of T*. DNA samples contained 0.5 mM (a) and 1 mM (b) duplex DNA (ODN^{1*} "C*-T*") and 1.5 equiv of $Hg(ClO_4)_2$ in aqueous buffer (200 mM $NaClO_4$, 50 mM cacodylic acid in H_2O / D_2O (9:1) at pH = 7.8). Equiv of Hg^{II} are given relative to mismatch.

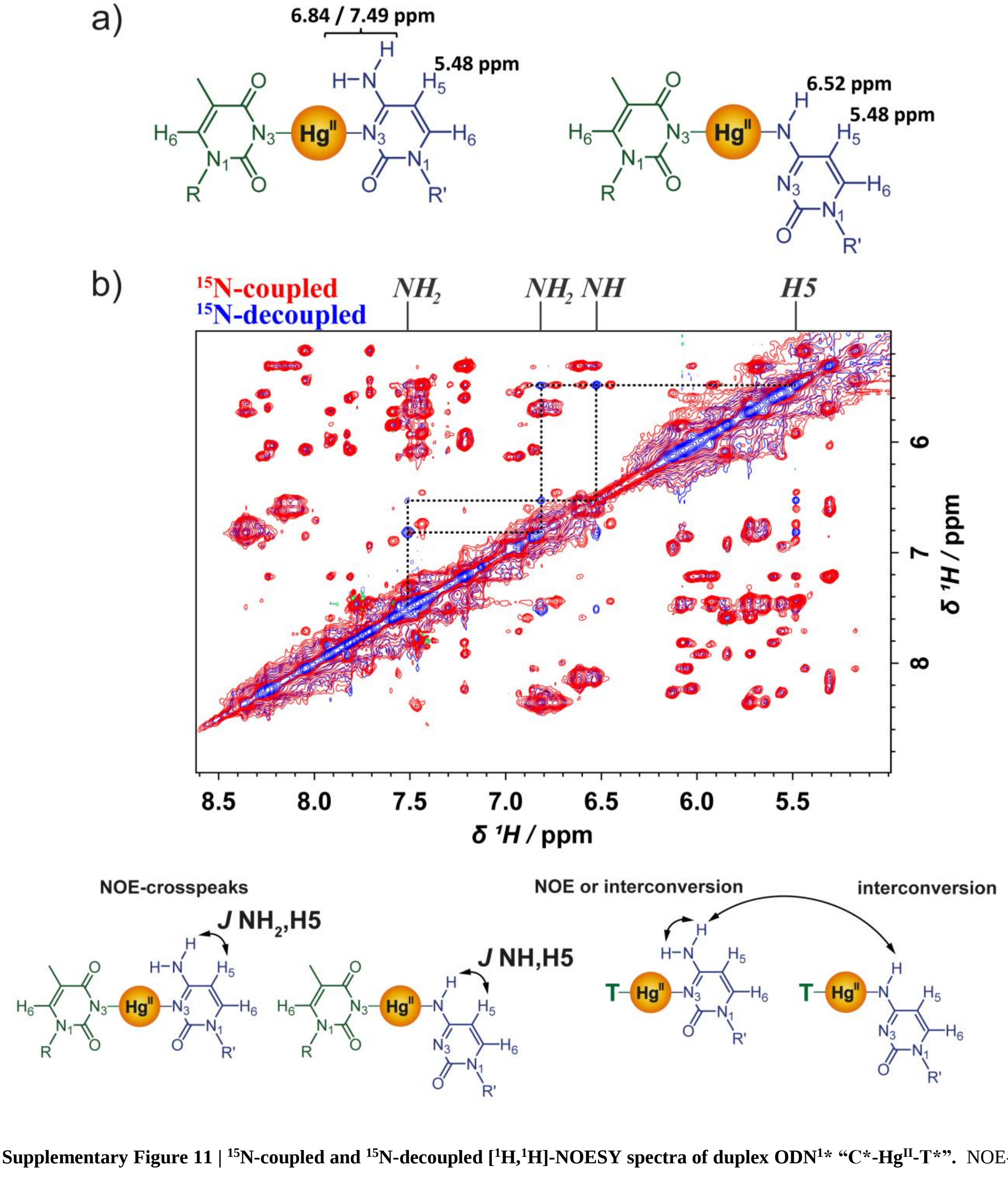

Supplementary Figure 11 | ^{15}N-coupled and ^{15}N-decoupled [^{1}H,^{1}H]-NOESY spectra of duplex ODN^{1*} "C*-Hg^{II}-T*". NOE-cross peak between (N4)NH^{minor} and cytosine H5 confirms ^{15}N-resonance assignment as being the deprotonated exocyclic amine of cytosine. a) Chemical shift values of cytosine H5, (N4)NH$_2^{major}$ and (N4)NH^{minor} derived from [^{15}N,^{1}H]-HSQC and band-selective, long-range [^{15}N,^{1}H]-HSQC spectra (Supplementary Figures 9 and 10). b) NH$_2$,H5 and NH,H5 NOE cross peaks and NH$_2$,NH exchange cross peaks observed in the ^{15}N-coupled (red) and ^{15}N-decoupled (blue) [^{1}H,^{1}H]-NOESY spectra at 25 °C. The DNA sample contained 1 mM duplex DNA (ODN^{1*} "C*-T*") and 3 mM of $Hg(ClO_4)_2$ (1.5 equiv Hg^{II} relative to mismatch) in aqueous buffer (200 mM $NaClO_4$, 50 mM cacodylic acid in H_2O / D_2O (9:1) at pH = 7.8).

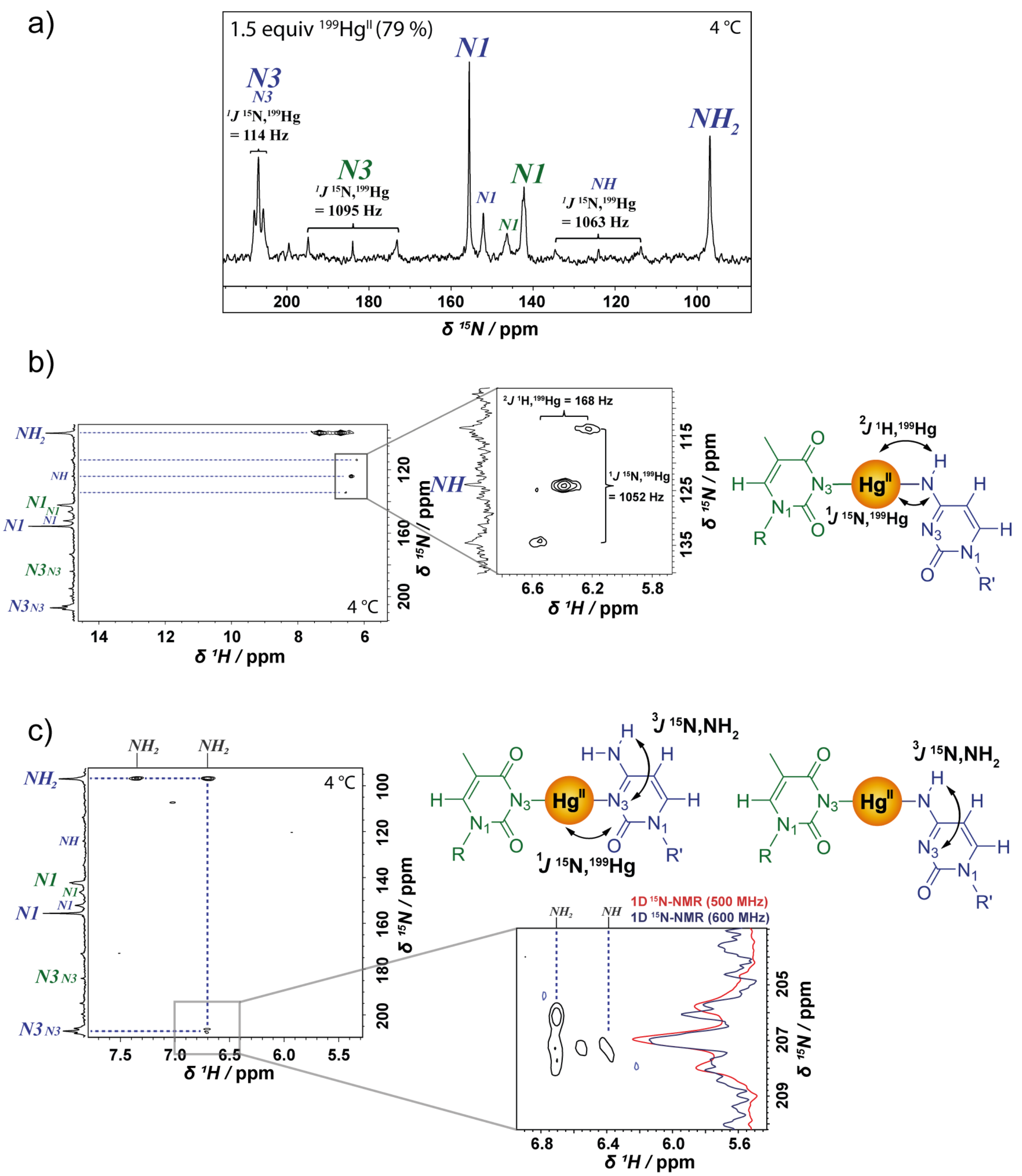

Supplementary Figure 12 | Unambiguous determination of metal-nucleobase connectivities. Changes in ^{15}N- and [^{15}N,^{1}H]-HSQC spectra of ODN^{1}* "C*-T*" upon addition of ^{199}Hg-isotopically enriched Hg(ClO$_4$)$_2$ provided direct evidence for Hg^{II}-nucleobase coordination sites. a) ^{1}J ^{15}N,^{199}Hg coupling according to ^{15}N NMR spectrum of ODN^{1}* "C*-T*" with 1.5 equiv of ^{199}Hg-enriched Hg(ClO$_4$)$_2$ (79 % enriched, Supplementary Figure 13). b) ^{1}J ^{15}N,^{199}Hg and ^{2}J ^{1}H,^{199}Hg coupling according to [^{15}N,^{1}H]-HSQC. c) ^{3}J ^{1}H,^{15}N coupling between N3^{major} and (N4)NH$_2$^{major} of cytosine and ^{3}J ^{1}H,^{15}N coupling between N3^{minor} and deprotonated (N4)NH^{minor} of cytosine observed in the band-selective, long-range [^{15}N,^{1}H]-HSQC spectrum. Blue indicates ^{15}N resonances of C* and green indicates ^{15}N resonances of T*. The DNA sample contained 1 mM duplex DNA and 3 mM of ^{199}Hg-enriched Hg(ClO$_4$)$_2$ (1.5 equiv Hg^{II} relative to mismatch) in aqueous buffer (200 mM NaClO$_4$, 50 mM cacodylic acid in H$_2$O / D$_2$O (9:1) at pH = 7.8), and the spectra were measured at 4 °C.

a)

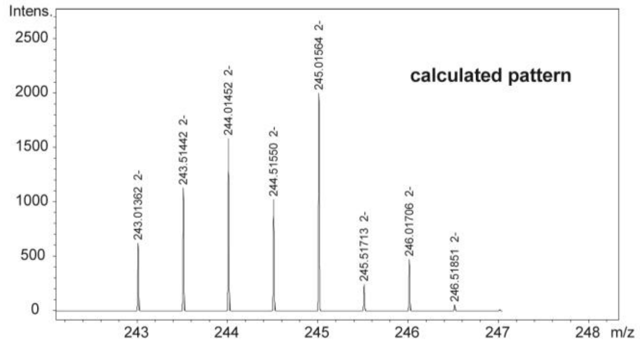

#	m/z	I	I %
1	242.01315	9	0.5
2	243.01362	623	31.2
3	243.51442	1129	56.4
4	244.01452	1584	79.2
5	244.51550	1022	51.1
6	245.01564	2000	100.0
7	245.51713	246	12.3
8	246.01706	474	23.7
9	246.51851	55	2.8
10	247.01940	10	0.5

b)

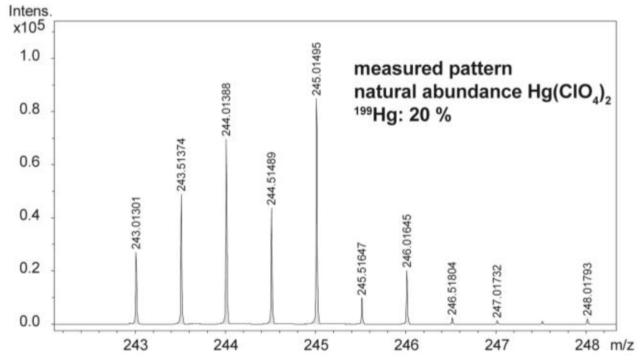

#	m/z	I	I %
1	243.01301	27110	31.9
2	243.51374	48892	57.6
3	244.01388	69689	82.1
4	244.51489	43677	51.4
5	245.01495	84906	100.0
6	245.51647	9834	11.6
7	246.01645	20313	23.9
8	246.51804	2457	2.9
9	247.01732	1309	1.5
10	247.51764	1002	1.2

c)

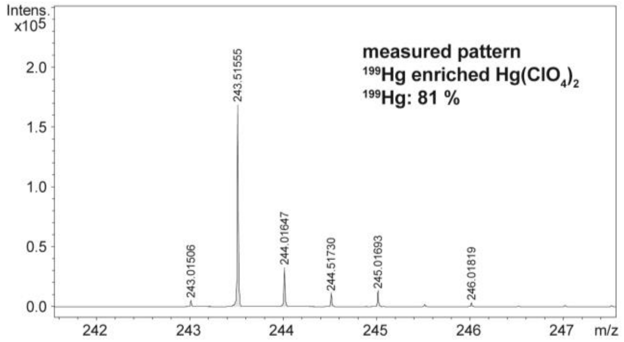

#	m/z	I	I %
1	243.01506	4966	2.9
2	243.51555	168697	100.0
3	244.01647	32522	19.3
4	244.51730	10812	6.4
5	245.01693	12804	7.6
6	245.51852	1601	0.9
7	246.01819	3148	1.9
8	247.01901	800	0.5

d)

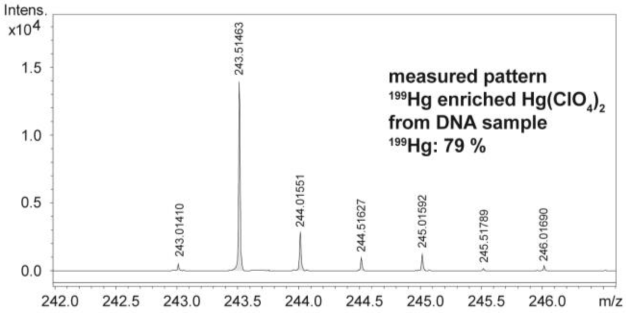

#	m/z	I	I %
1	243.01410	485	3.5
2	243.51463	13943	100.0
3	244.01551	2887	20.7
4	244.51627	947	6.8
5	245.01592	1211	8.7
6	245.51789	159	1.1
7	246.01690	372	2.7

Supplementary Figure 13 | HR-ESI-MS analysis of Hg^{II}-$EDTA^{2-}$ complex. a) Calculated isotopic pattern of Hg^{II}-$EDTA^{2-}$. b) Measured isotopic pattern of natural abundance Hg^{II}-$EDTA^{2-}$. c) Measured isotopic pattern of ^{199}Hg-enriched Hg^{II}-$EDTA^{2-}$. d) Measured isotopic pattern of ^{199}Hg-enriched Hg^{II}-$EDTA^{2-}$ from “C*-T*” duplex DNA.

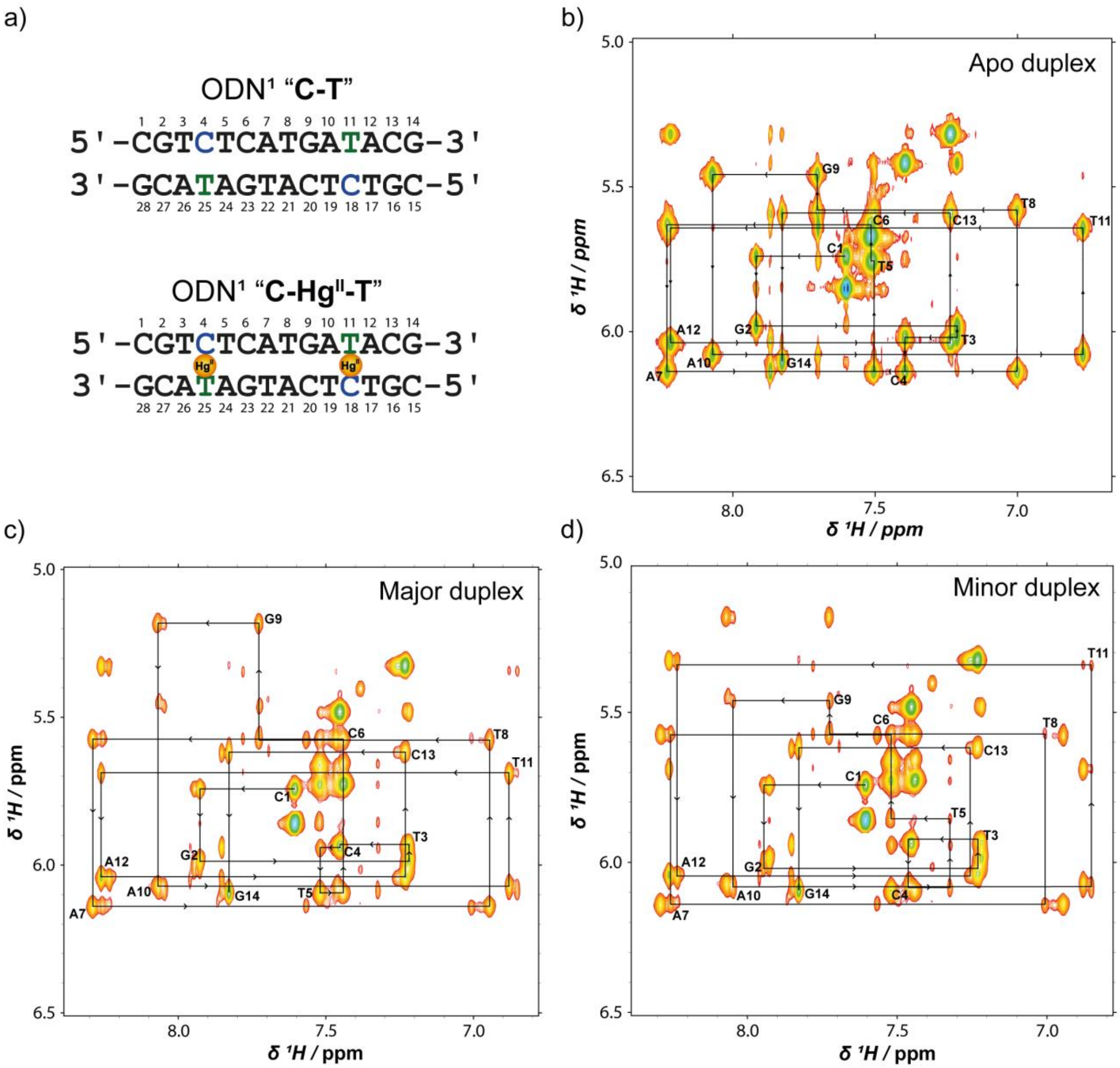

Supplementary Figure 14 | Sequential walking along H1' and aromatic protons in [1H,1H]-NOESY spectra. a) ODN1 "C-T" and ODN1 "C-HgII-T" sequence. Blue and green bases indicate C-T mismatches. Sequential walking along H1' and aromatic protons in the [1H,1H]-NOESY spectra through the entire sequence from residues C1 to G14 for apo- (b), major- (c) and minor (d) duplex DNA. The DNA sample of the apo duplex (ODN1 "C-T") contained 0.3 mM duplex DNA in an aqueous solution of $NaClO_4$ (50 mM, D_2O, pD = 7.75) and was measured at 25 °C. The DNA sample of the "C-HgII-T" contained 0.4 mM duplex DNA (ODN1 "C-T") and 1.2 mM HgII (1.5 equiv HgII relative to mismatch) in an aqueous solution of $NaClO_4$ (50 mM, D_2O, pD = 7.75) and was measured at 25 °C.

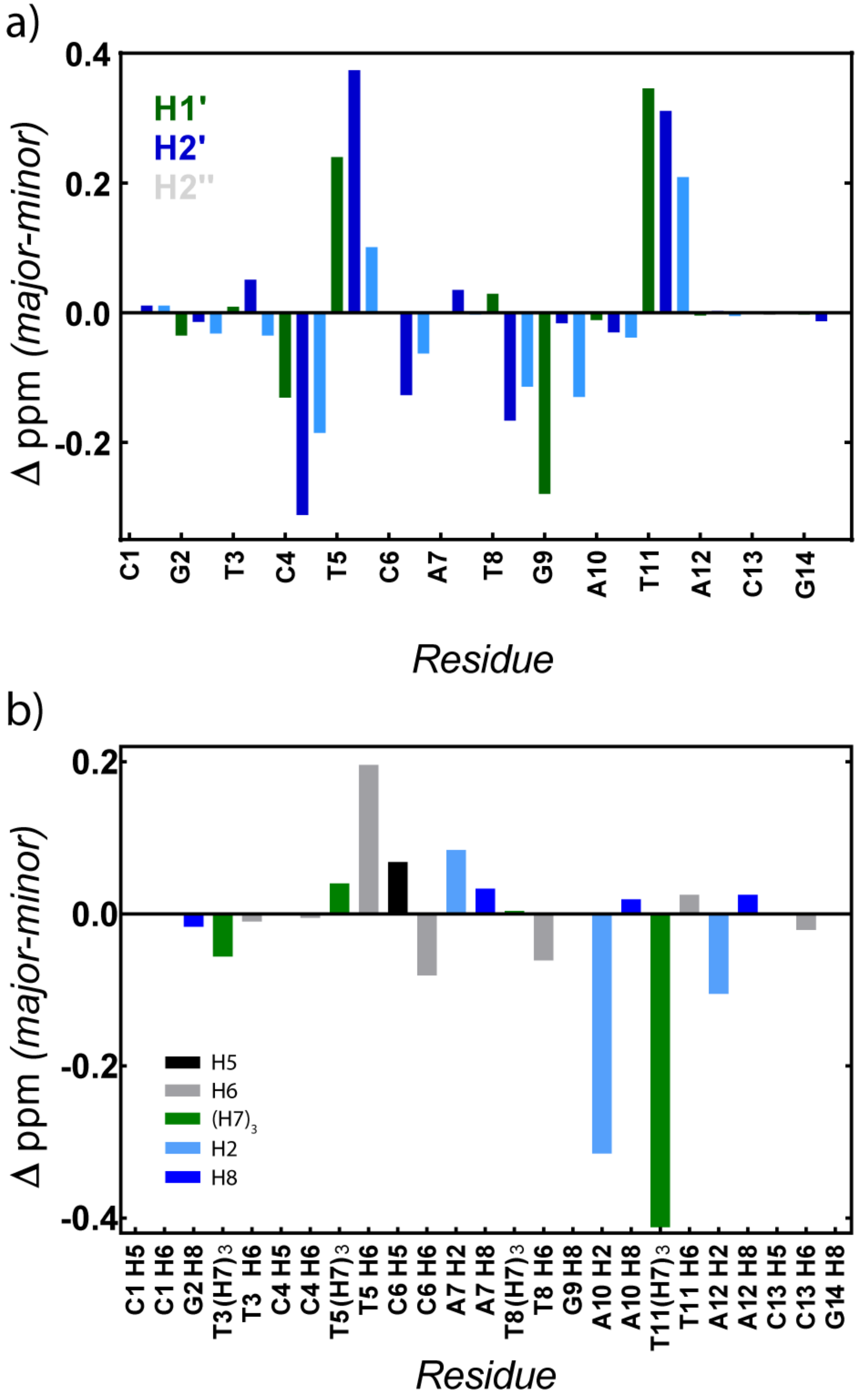

Supplementary Figure 15 | Chemical shift differences between major and minor duplexes. a) Chemical shift difference of H1' (green), H2' (blue), and H2'' (grey) sugar proton signals, and b) of aromatic nucleobase signals H5 (black), H6 (grey), $(H7)_3$ (green), H2 (light blue), and H8 (dark blue). Δppm calculated as ppm(major) - ppm(minor). The DNA sample contained 0.4 mM duplex DNA (ODN[1] "C-T") and 1.2 mM Hg^{II} (1.5 equiv Hg^{II} relative to mismatch) in an aqueous solution of $NaClO_4$ (50 mM, D_2O, pD = 7.75) and was measured at 25 °C. Source data are provided as a Source Data file.

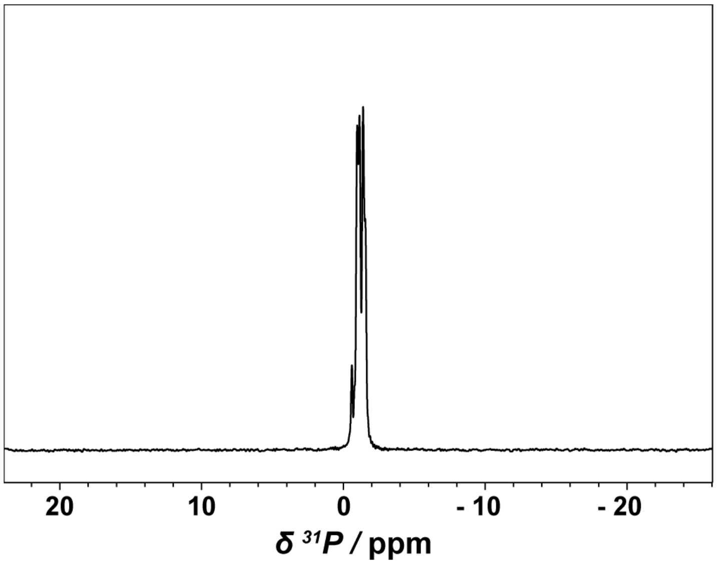

Supplementary Figure 16 | ^{31}P NMR of duplex ODN[1] "C-T". The DNA sample contained 0.4 mM duplex DNA and 1.2 mM Hg^{II} (1.5 equiv Hg^{II} relative to mismatch) in an aqueous solution of $NaClO_4$ (50 mM, D_2O, pD = 7.75) at 25 °C.

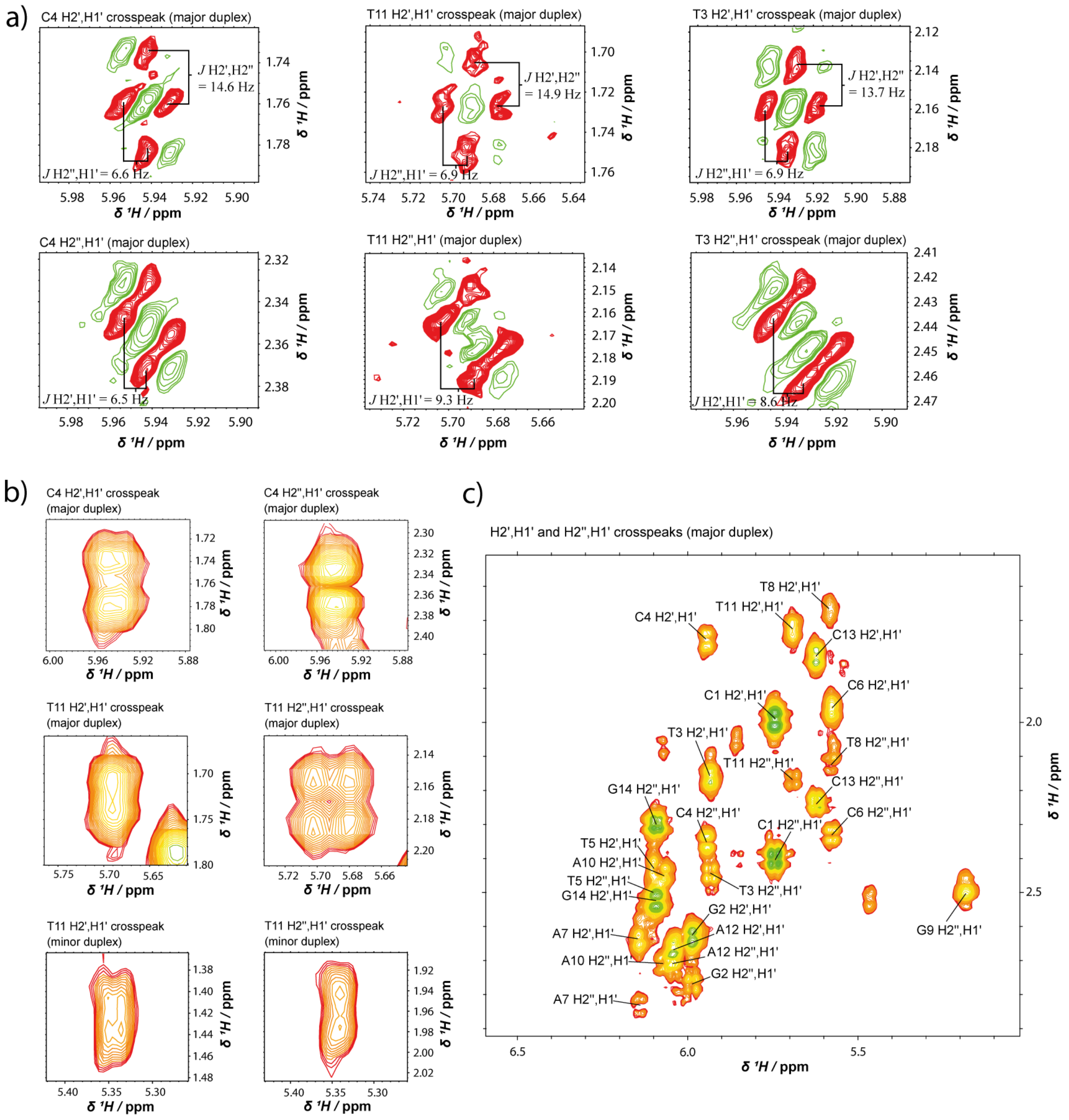

Supplementary Figure 17 | [^{1}H,^{1}H]-E.COSY and magnitude-processed [^{1}H,^{1}H]-COSY. a) Selected H1',H2' and H1',H2" cross peaks in the [^{1}H,^{1}H]-E.COSY spectrum used to determine coupling constants for Hg^{II}-bound residues C4 and T11 and coupling constants of T3 as a selected example for a Hg^{II} unbound residue. b) H1',H2' and H1',H2" cross peaks in the magnitude processed [^{1}H,^{1}H]-COSY spectrum for Hg^{II}-coordinated residues. c) H1',H2' and H1',H2" cross peaks of the major duplex in the magnitude processed [^{1}H,^{1}H]-COSY spectrum illustrating similar shape of T11 cross peaks of the minor duplex species and C4 cross peaks of the major duplex species. The DNA sample contained 0.4 mM duplex DNA (ODN[1] "C-T") and 1.2 mM Hg^{II} (1.5 equiv Hg^{II} relative to mismatch) in an aqueous solution of $NaClO_4$ (50 mM, D_2O, pD = 7.75) and was measured at 25 °C.

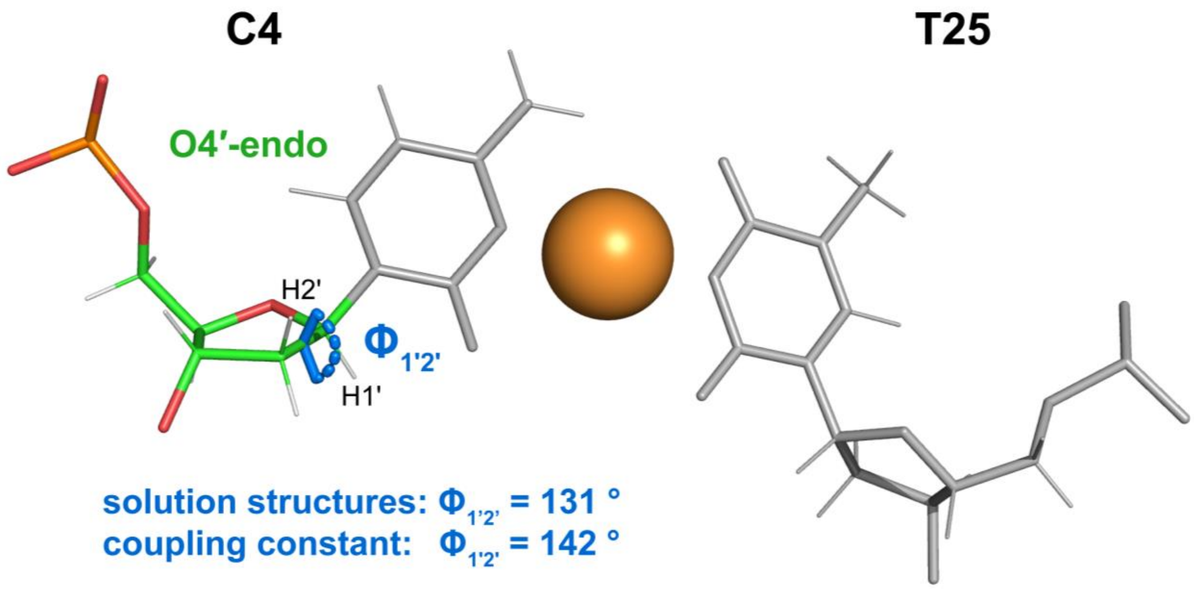

Supplementary Figure 18 | O4'-endo sugar pucker at the Hg^{II}-bound C4 residue of the major duplex species. The dihedral angle ($\Phi_{1'2'}$ = 131 ° ± 5 °) determined from solution structures was in excellent agreement with the dihedral angle calculated from the experimentally determined coupling constant ($\Phi_{1'2'}$ = 142 °) according to the Karplus relationship (Supplementary Equation 1).[2,3] This value is inconsistent with both C2'-endo (^{3}J H1',H2' = 9.5 Hz)[4] and C3'-endo (^{3}J H1',H2' = 1.5 Hz)[4] sugar conformations (Supplementary Table 5). According to the Karplus relationship a pseudorotation phase angle (P)[2] of 85° and 202° was calculated for the Hg^{II}-coordinated cytosine residue of the major duplex species. A pseudorotation phase angle of 85° corresponds to an O4'-endo sugar pucker.[2,5] $\Phi_{1'2'}$ determined from the solution structures represents the mean value and standard deviation of the 20 lowest energy conformations. Figure was generated with PyMol.[6]

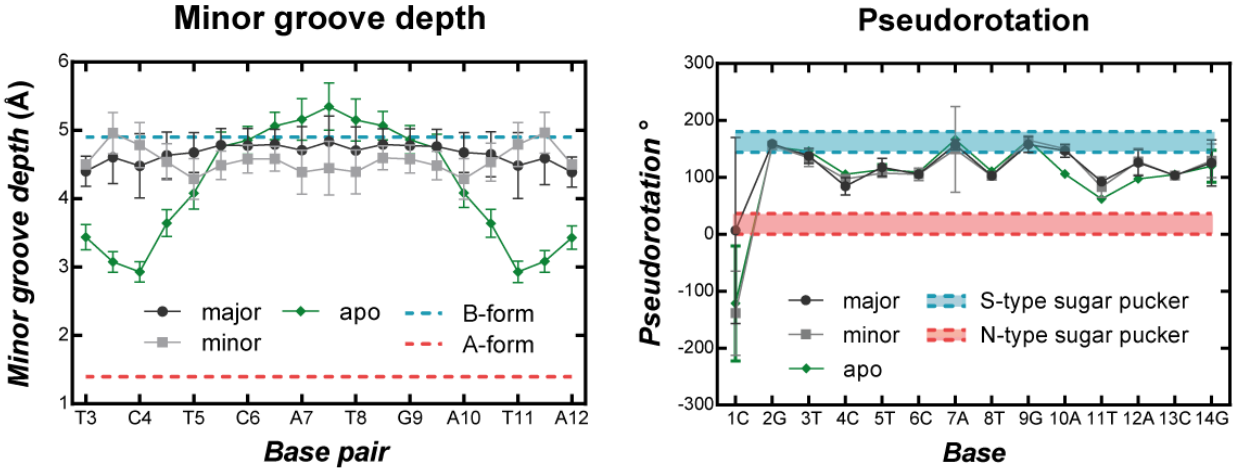

Supplementary Figure 19 | Minor groove depth and pseudorotation phase angles. Parameters were analyzed using Curves+[7] and represent mean and standard deviation of the 20 lowest energy conformations of major- (black), minor- (grey) and apo (green) duplex species. Reference A-form (red) and B-form (blue) values for pseudorotation were taken from (ref 8) and for minor groove depth from (ref 7) which analyzed crystal structures of A-DNA (PDB ID 1d13[9]) and B-DNA (PDB ID 1bna[10]) dodecamers. Source data are provided as a Source Data file.

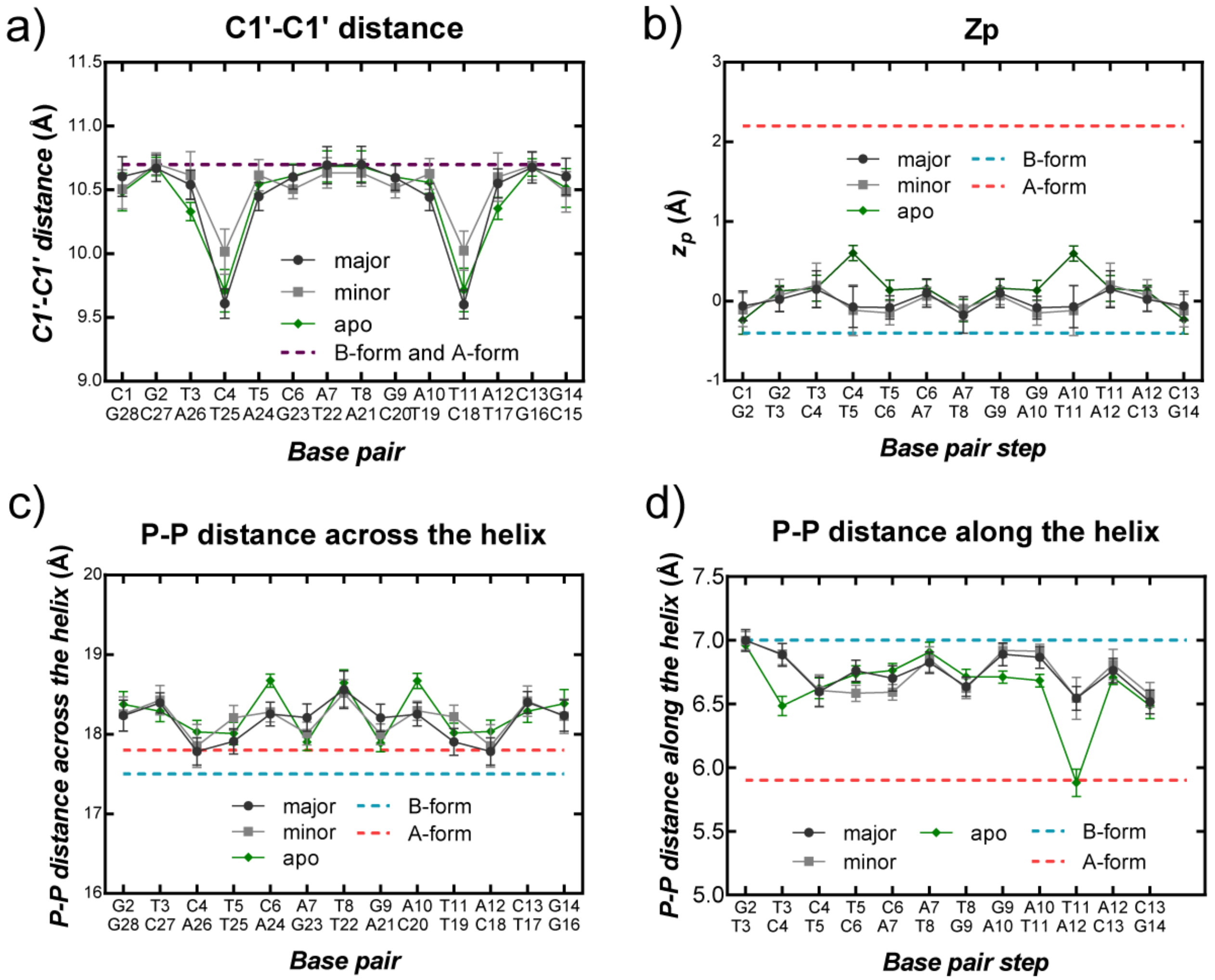

Supplementary Figure 20 | Helical parameters. a) C1'- C1' distances in each base pair. b) Displacement of phosphate group from the middle of a base pair step (Zp). c) P-P distance across the helix. d) P-P distance along the helix. C1'- C1' distance, phosphate displacement, and P-P distance along the helix were calculated using 3DNA (a-b)[11,12] and P-P distance across the helix were determined using PyMOL.[6] Standard A-form (red) and B-form (blue) values for C1'- C1' distance and phosphate displacement were taken from (ref 13) and (ref 14), respectively. Reference A-form and B-form values for P-P distance across the helix were determined by generating an ideal A-form and B-form using scfbio-iitd software[15] and for P-P distance along the helix from (ref 11,12). Reported values are mean and standard deviation of the 20 lowest energy conformations of major- (black), minor- (grey) and apo (green) duplex species. Source data are provided as a Source Data file.

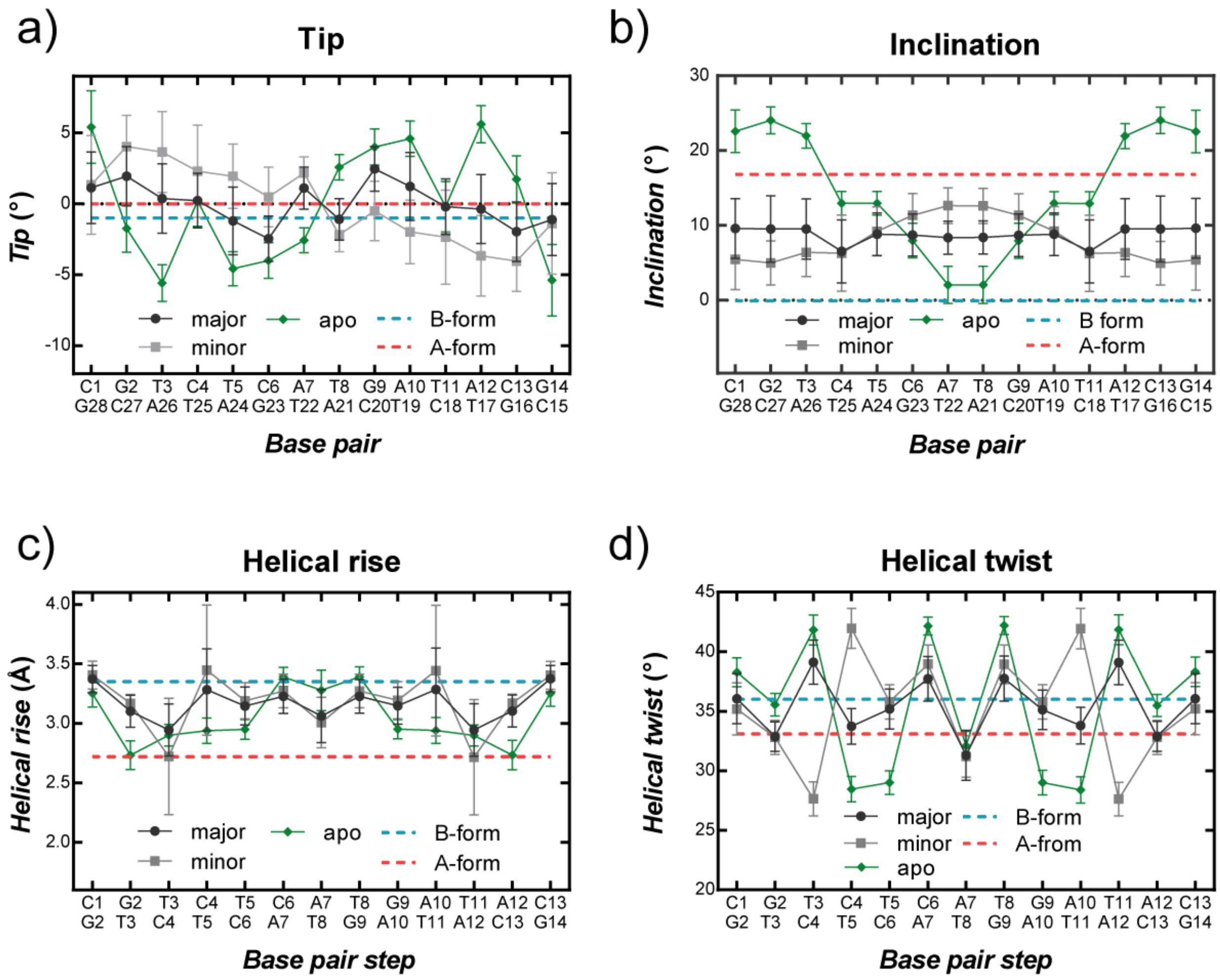

Supplementary Figure 21 | Base pair-axis parameters tip and inclination and helical parameters helical rise and helical twist. a) tip (rotation around the long axis of the base pair) and b) inclination of base pairs with respect to the helical axis (rotation around the short axis of the base pair). c) Helical rise and d) helical twist angles. All parameters were analyzed using Curves+[7] and represent mean and standard deviation of the 20 lowest energy conformations of major- (black), minor- (grey) and apo (green) duplex species. Reference A-form (red) and B-form (blue) values were taken from (ref 7) which analyzed crystal structures of A-DNA (PDB ID 1d13[9]) and B-DNA (PDB ID 1bna[10]) dodecamers. Source data are provided as a Source Data file.

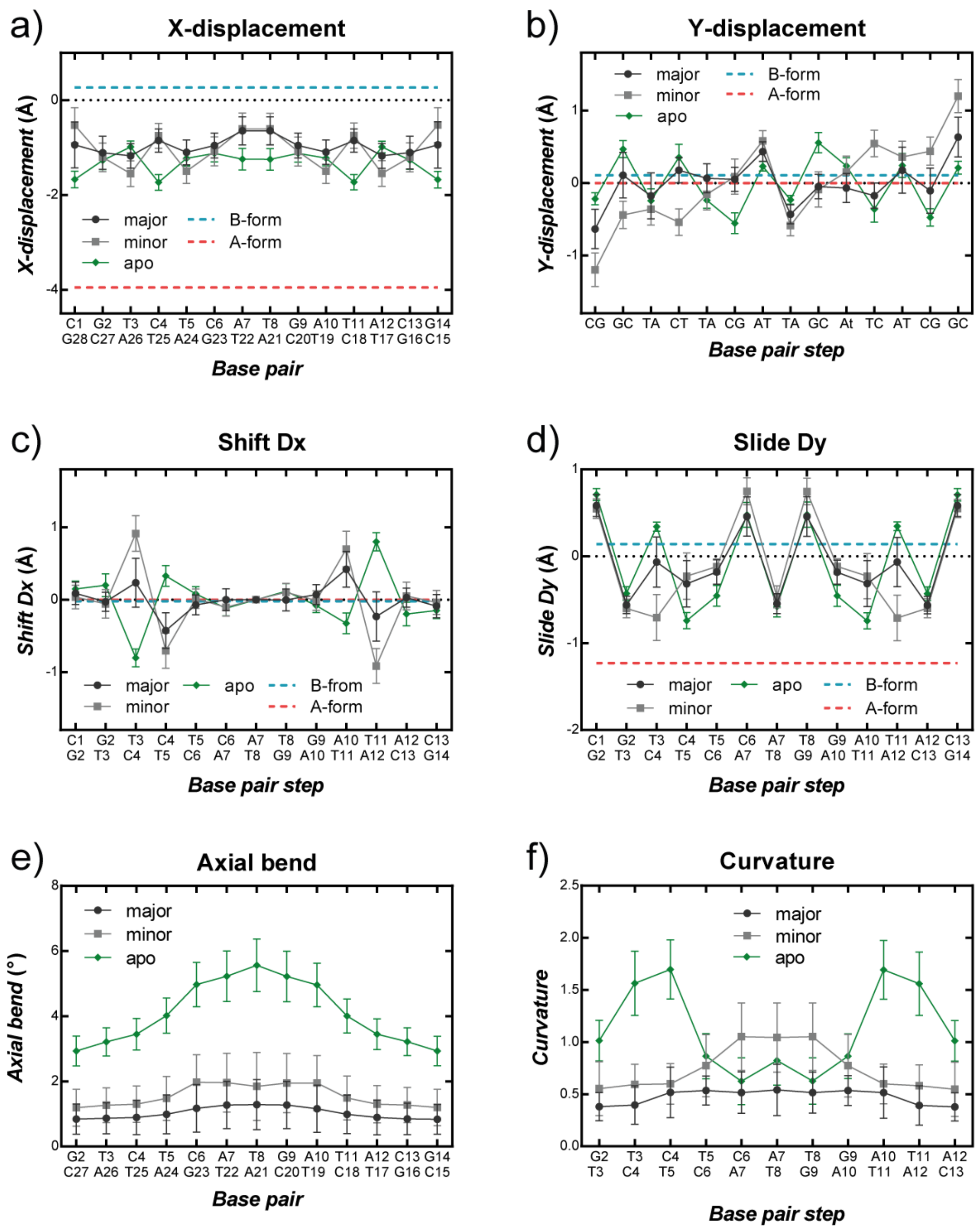

Supplementary Figure 22 | Base pair parameters X- and Y-displacement, shift and slide and curvature analysis. a) Translation of base pair towards grooves (a, x-displacement) and perpendicular to the grooves (b, y-displacement). Local dimer step parameters shift (c, Dx) and slide (d, Dy) describing relative positioning of successive base pairs with respect to the x- and y-axis of the relative base pair reference frame. e) axial bend and f) curvature analysis of major-, minor-, and apo duplexes. Curvature is a dimensionless quantity normalizing the curvature to DNA on the nucleosome (curvature ~ 1).[16] All parameters were analyzed using Curves+[7] and represent mean and standard deviation of the 20 lowest energy conformations of major- (black), minor- (grey) and apo (green) duplex species. Reference A-form (red) and B-form (blue) values were taken from (ref 7) which analyzed crystal structures of A-DNA (PDB ID 1d13[9]) and B-DNA (PDB ID 1bna[10]) dodecamers. Source data are provided as a Source Data file.

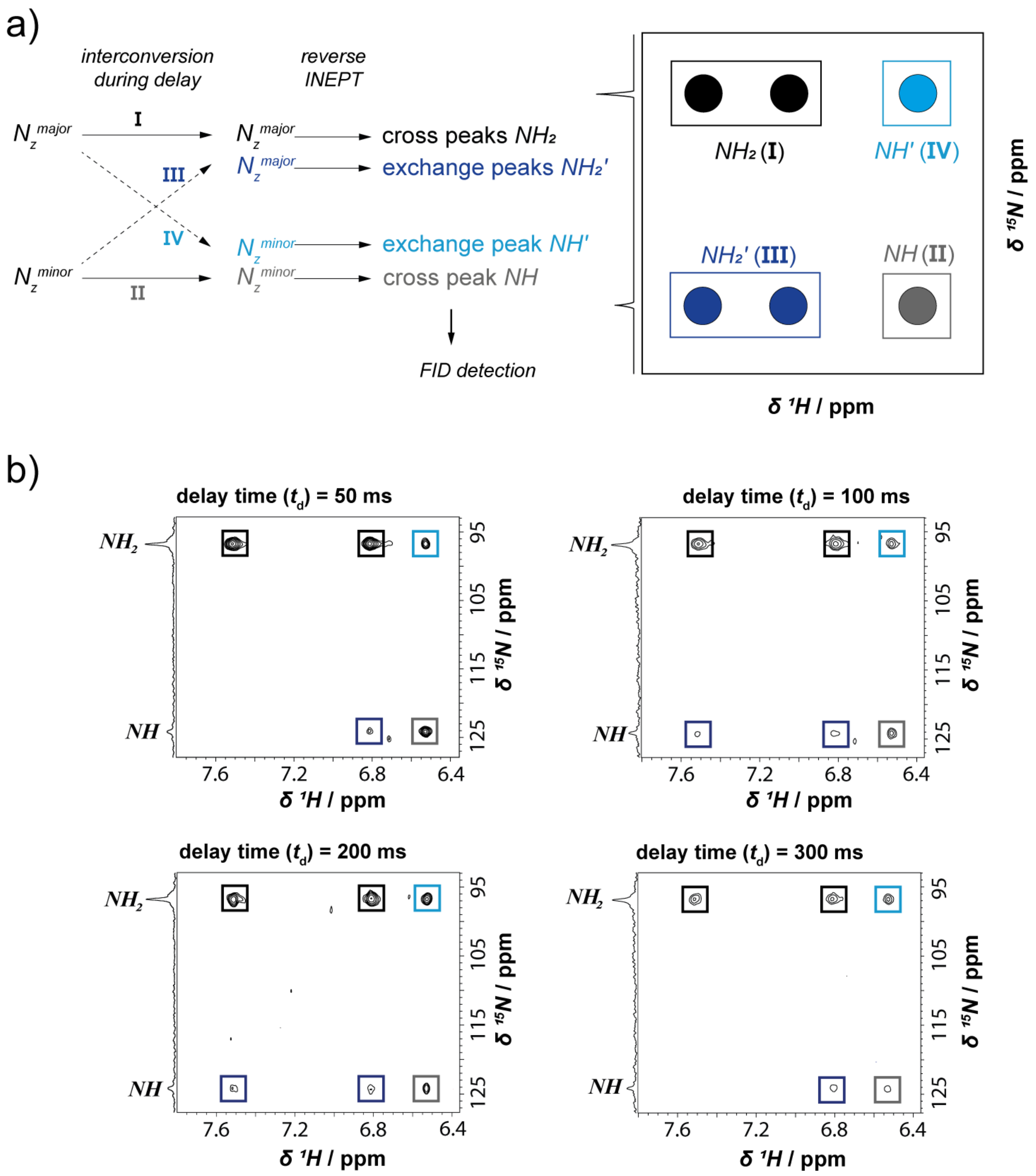

Supplementary Figure 23 | Dynamic changes in local metal ion coordination. a) Schematic description of a [^{15}N,^{1}H]-HSQC experiment with a delay introduced before the reverse INEPT showing all possible ^{15}N z-z exchange during delay (I-IV), followed by INEPT transfer. Exchange I and II give cross peaks *NH*$_2$ (black) and *NH* (grey), corresponding to those of [^{15}N,^{1}H]-HSQC experiments without delay. Interconversion of major- and minor structures during the delay results in exchange III and IV to give exchange cross peaks *NH*$_2$*'* (dark blue) and *NH'* (light blue). b) Interconversion of major- and minor structures according to exchange crosspeaks in [^{15}N,^{1}H]-HSQC spectra with various delay times before reverse INEPT at 25 °C. The DNA sample contained 0.5 mM duplex DNA (ODN[1] "C-T") and 1.5 mM of $Hg(ClO_4)_2$ (1.5 equiv Hg^{II} relative to mismatch) in aqueous buffer (200 mM $NaClO_4$, 50 mM cacodylic acid in H_2O / D_2O (9:1) at pH = 7.8).

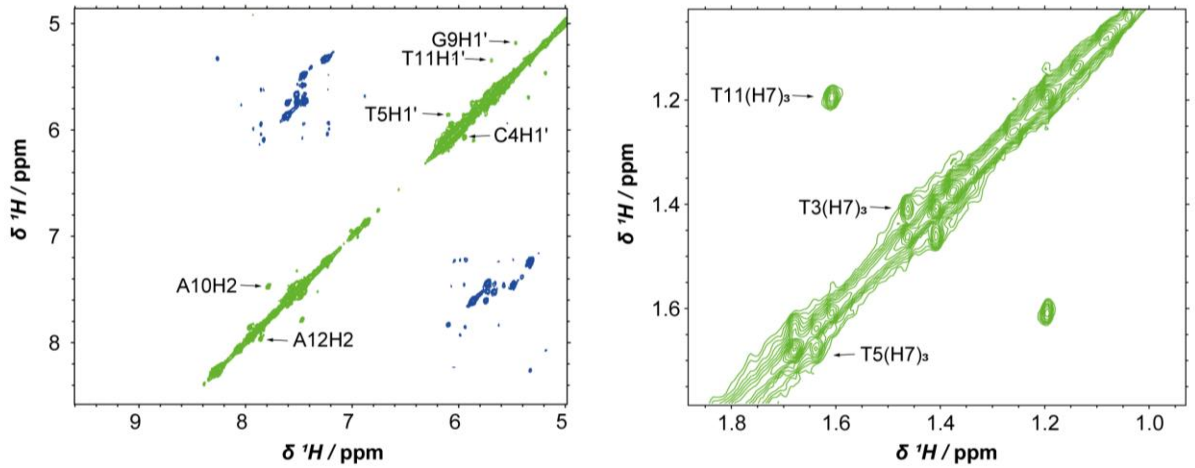

Supplementary Figure 24 | Sections of the [^{1}H,^{1}H]-ROESY spectrum of ODN^1 "C-T". Exchange signals can be distinguished from ROE cross peaks by the phase. ROE cross peaks are opposite in phase to the diagonal and exchange cross peaks in phase to the diagonal. The DNA sample contained 0.4 mM duplex DNA and 1.2 mM Hg^{II} (1.5 equiv Hg^{II} relative to mismatch) in an aqueous solution of $NaClO_4$ (50 mM, D_2O, pD = 7.75) and was measured at 25 °C.

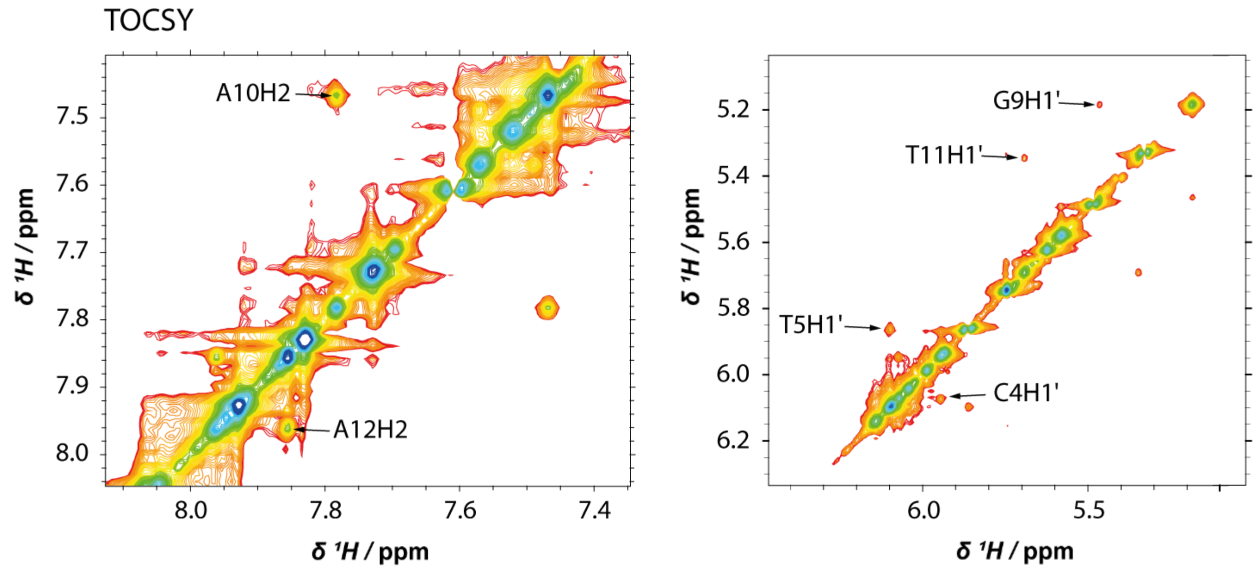

Supplementary Figure 25 | Exchange cross peaks in [^{1}H,^{1}H]-TOCSY spectrum. The DNA sample contained 0.4 mM duplex DNA (ODN^1 "C-T") and 1.2 mM $Hg(ClO_4)_2$ (1.5 equiv Hg^{II} relative to mismatch) in an aqueous solution of $NaClO_4$ (50 mM, D_2O, pD = 7.75) and was measured at 25 °C.

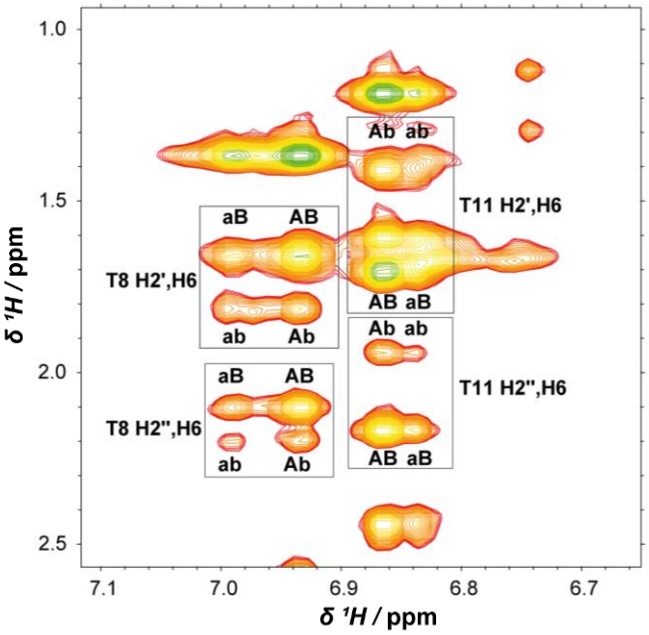

Supplementary Figure 26 | Exchange-mediated NOE cross peaks in the [^{1}H,^{1}H]-NOESY spectrum. 'AB' and 'ab' are defined as NOE cross peaks of major- and minor duplex species, respectively, and 'Ab' and 'aB' exchange-mediated NOE cross peaks (Supplementary Figure 36). The DNA sample contained 1 mM duplex DNA (ODN[1] "C-T") and 3 mM of ^{199}Hg-enriched $Hg(ClO_4)_2$ (1.5 equiv $^{199}Hg^{II}$ relative to mismatch) in aqueous buffer (200 mM $NaClO_4$, 50 mM cacodylic acid in H_2O / D_2O (9:1) at pH = 7.8) and was measured at 25 °C.

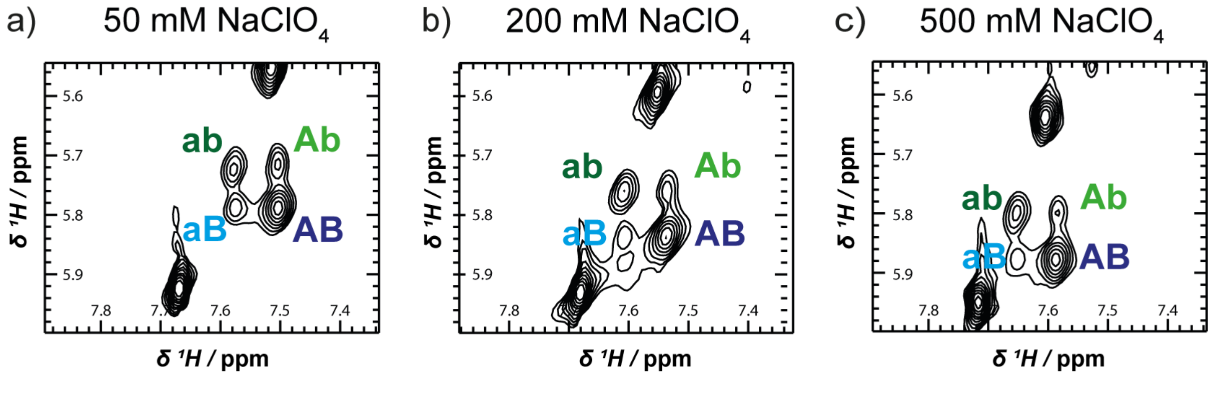

Supplementary Figure 27 | Decrease in overall exchange rate of major- and minor duplexes with increasing ionic strength. Cytosine C4 H5/H6 [^{1}H,^{1}H]-TOCSY resonances of major- (AB, dark blue) and minor (ab, dark green) and their exchange-mediated cross peaks (aB, light blue) and (Ab, light green) in the presence of a) 50 mM $NaClO_4$, b) 200 mM $NaClO_4$, and c) 500 mM $NaClO_4$. Decreasing signal intensities of the exchange-mediated cross peaks (aB and Ab) compared to [^{1}H,^{1}H]-TOCSY cross peaks (AB and ab) revealed a slower overall interconversion rate of the helices with increasing ionic strength. The DNA samples contained 0.96 mM duplex DNA and 2.88 mM of $Hg(ClO_4)_2$ (1.5 equiv Hg^{II} relative to mismatch) in aqueous buffer (50 mM, 200 mM or 500 mM $NaClO_4$, 50 mM cacodylic acid in H_2O / D_2O (9:1) at pH = 7).

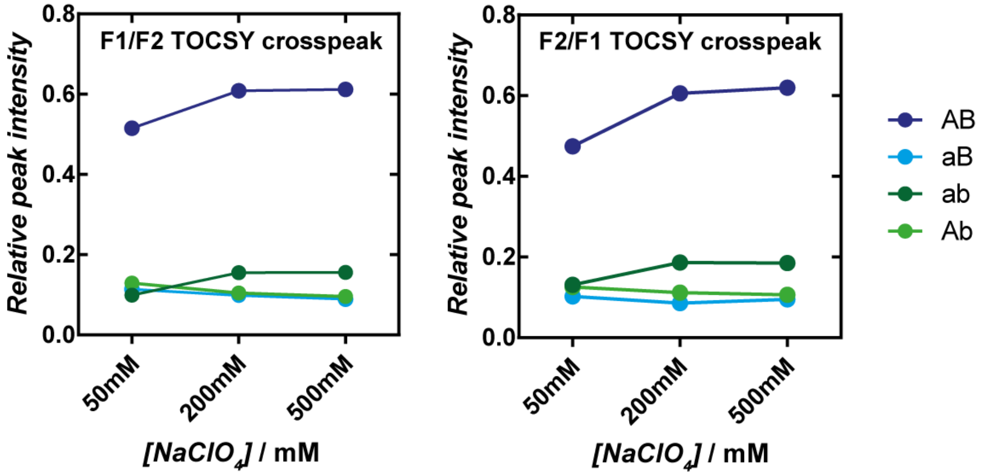

Supplementary Figure 28 | Relative peak intensities of F1/F2- and F2/F1 cross peaks at variable ionic strength. For definitions of labels see Supplementary Figure 27. Source data are provided as a Source Data file.

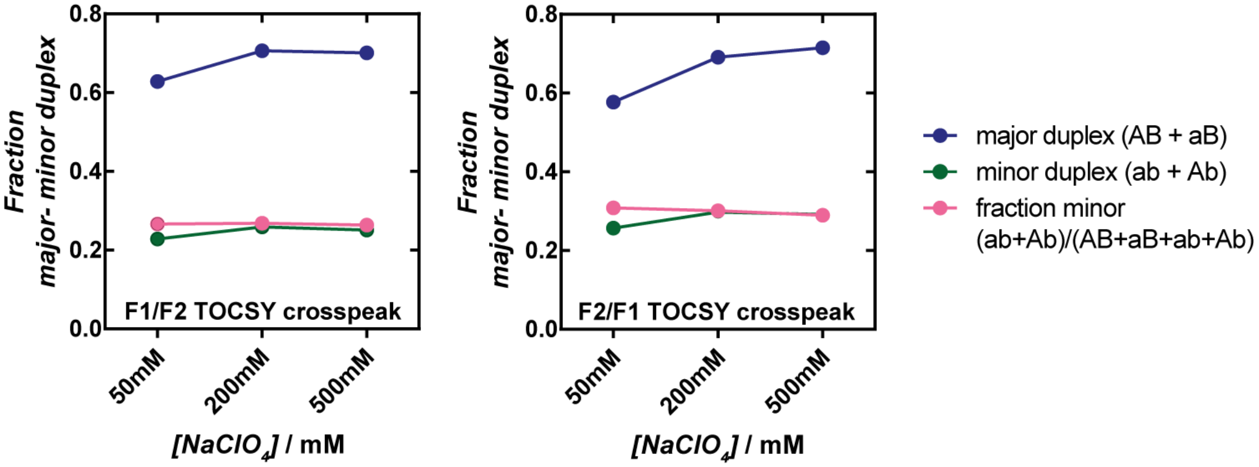

Supplementary Figure 29 | Fraction minor duplex species at variable ionic strength. Sum of integrals of the major- (AB + aB, blue) and minor species (ab + Ab, green), and the fraction minor species ((ab + Ab)/(AB + aB + ab + Ab), pink) of F1/F2 and F2/F1 cross peaks at variable ionic strength. Source data are provided as a Source Data file.

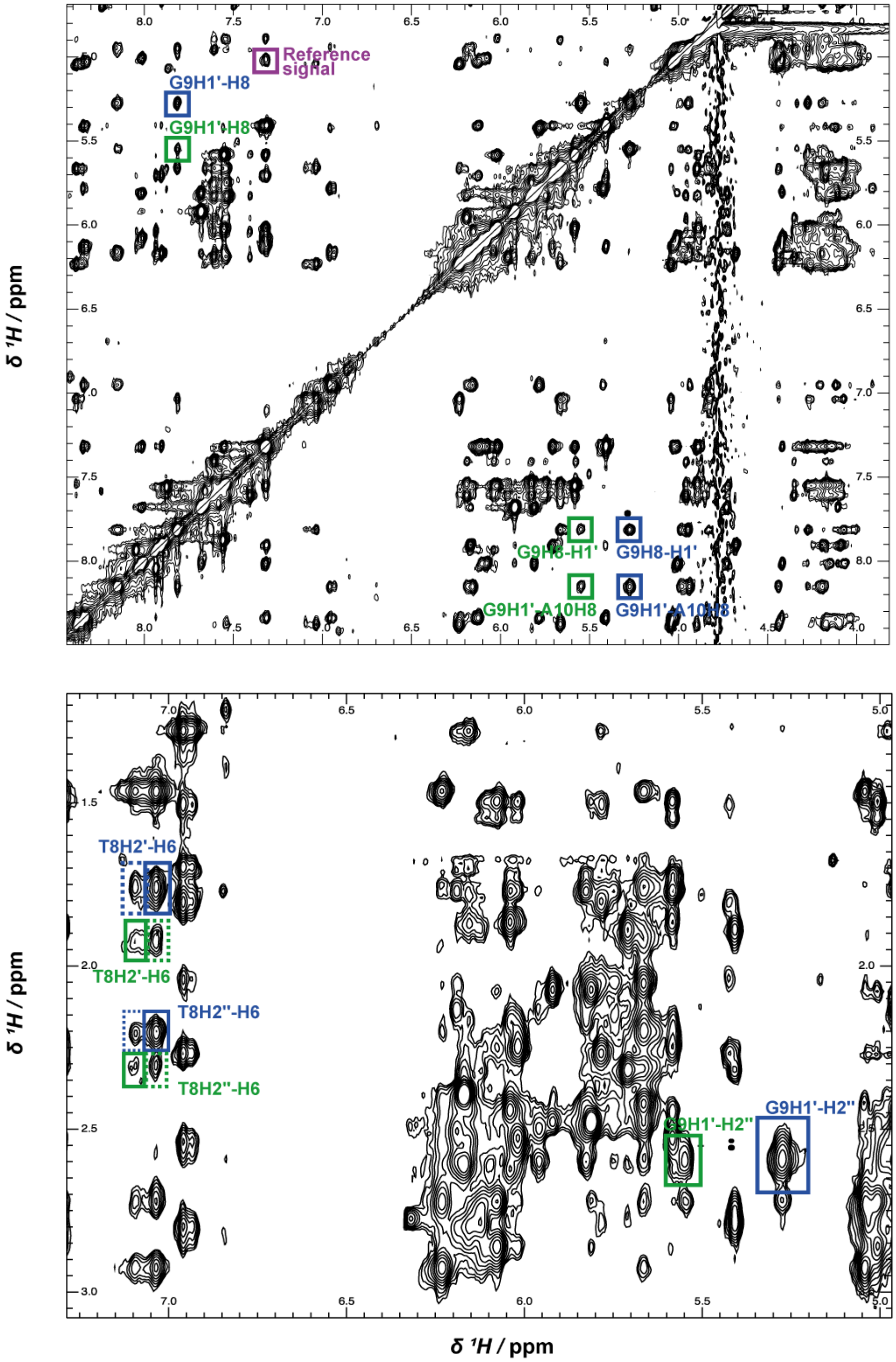

Supplementary Figure 30 | NOE cross peaks used to determine the fraction of minor duplex species at variable ionic strength. NOE cross peaks of the major duplex species are depicted in blue and of the minor duplex species in green. NOE cross peak of the selected reference signal (A12H3' – C13H6) is depicted in purple. The DNA samples contained 0.96 mM duplex DNA and 2.88 mM of $Hg(ClO_4)_2$ (1.5 equiv Hg^{II} relative to mismatch) in aqueous buffer (50 mM, 200 mM or 500 mM $NaClO_4$, 50 mM cacodylic acid in H_2O / D_2O (9:1) at pH = 7).

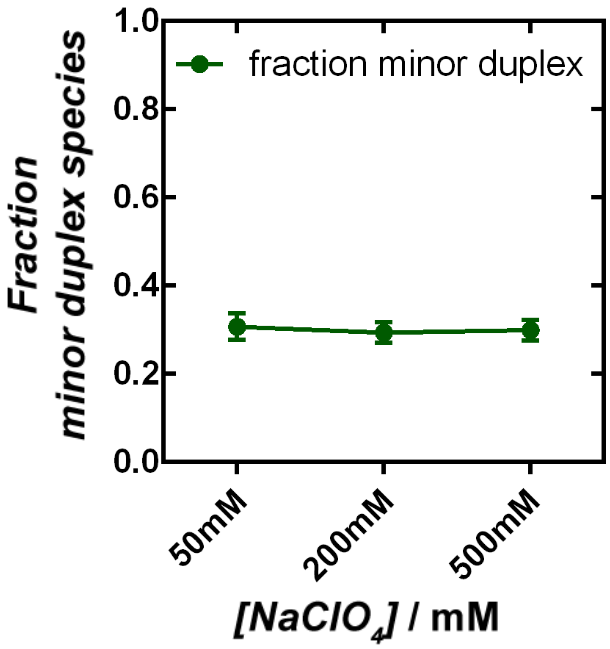

Supplementary Figure 31 | Fraction minor duplex species at variable ionic strength. Six well resolved NOE signals in the [^{1}H,^{1}H]-NOESY spectra of metallo duplex samples were used to determine fraction minor duplex species (Supplementary Figure 30). The DNA samples contained 0.96 mM duplex DNA and 2.88 mM of $Hg(ClO_4)_2$ (1.5 equiv Hg^{II} relative to mismatch) in aqueous buffer (50 mM, 200 mM or 500 mM $NaClO_4$, 50 mM cacodylic acid in H_2O / D_2O (9:1) at pH = 7). The data represent the mean value from six NOE signals and standard deviation is indicated by error bars. Source data are provided as a Source Data file.

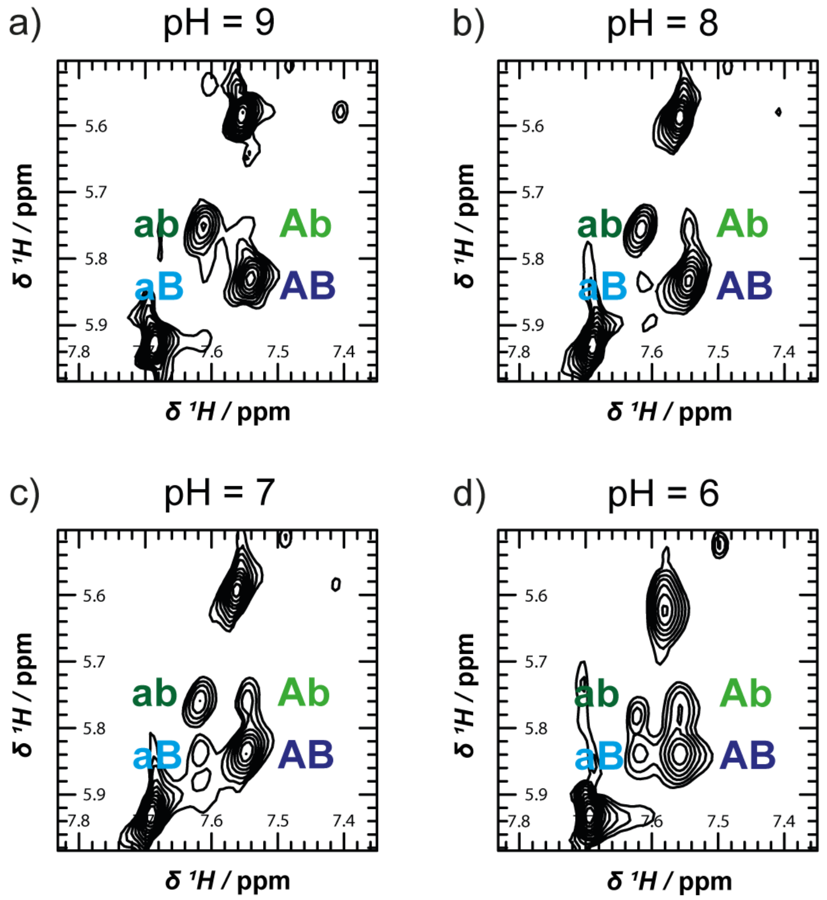

Supplementary Figure 32 | Decrease in the overall exchange rate of major- and minor duplexes with increasing pH. Cytosine C4 H5/H6 [^{1}H,^{1}H]-TOCSY resonances of major- (AB, dark blue) and minor (ab, dark green) and their exchange-mediated cross peaks (aB, light blue) and (Ab, light green) at a) pH = 9, b) pH = 8, c) pH = 7, and d) pH = 6. Decreasing signal intensities of the exchange-mediated cross peaks (aB and Ab) compared to [^{1}H,^{1}H]-TOCSY cross peaks (AB and ab) revealed a slower overall interconversion rate of the helices with increasing pH. DNA samples contained 0.76 mM duplex DNA and 2.28 mM of $Hg(ClO_4)_2$ (1.5 equiv Hg^{II} relative to mismatch) in aqueous buffer (200 mM $NaClO_4$, 50 mM cacodylic acid in H_2O / D_2O (9:1) at pH = 6 – 9).

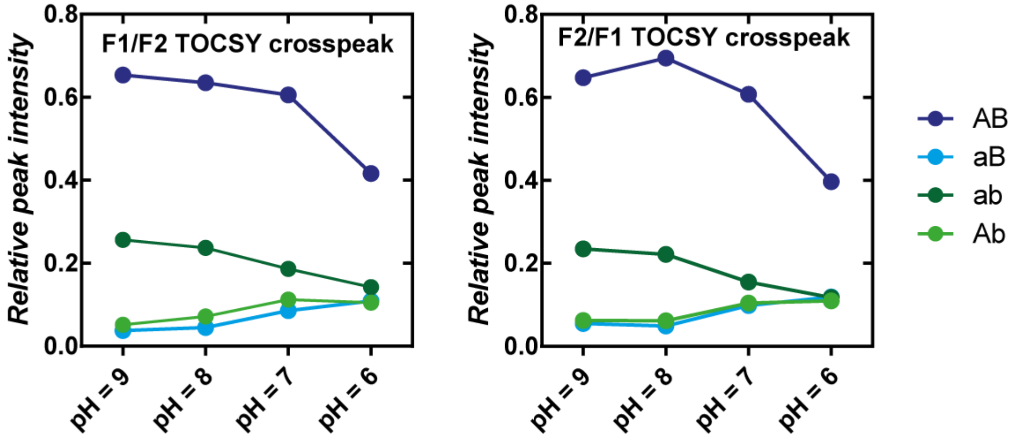

Supplementary Figure 33 | Relative peak intensities of F1/F2- and F2/F1 cross peaks at variable pH. For definitions of labels see Supplementary Figure 32. Source data are provided as a Source Data file.

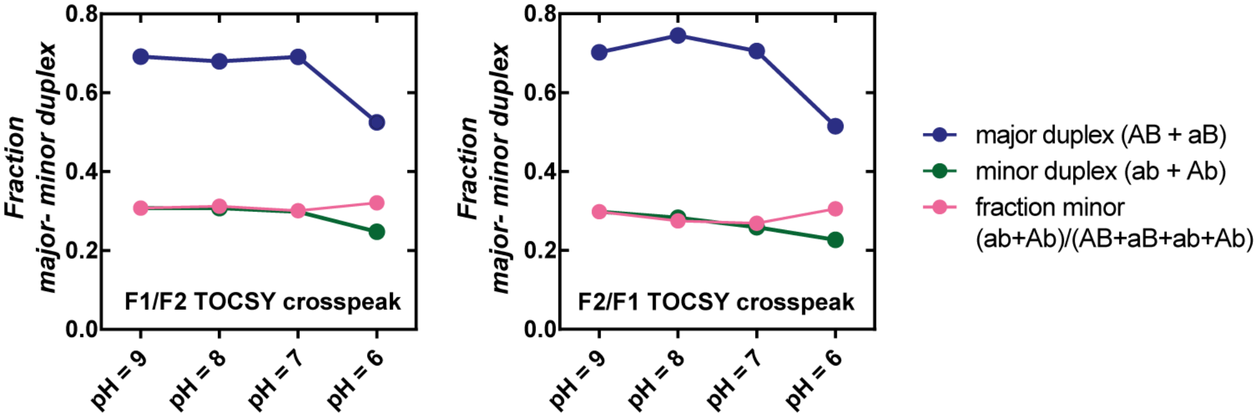

Supplementary Figure 34 | Fraction minor duplex species at variable pH. Sum of integrals of the major- (AB + aB, blue) and minor species (ab + Ab, green), and the fraction minor species ((ab + Ab)/(AB + aB + ab + Ab), pink) of the F1/F2- and F2/F1 cross peaks at variable pH. Source data are provided as a Source Data file.

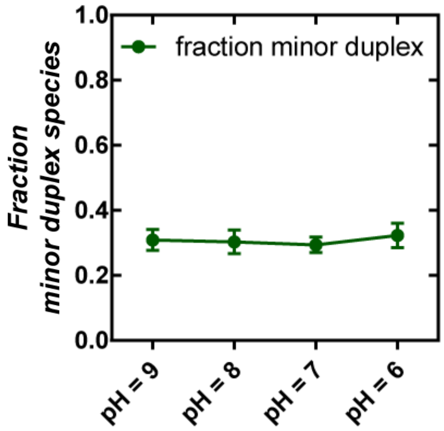

Supplementary Figure 35 | Fraction minor duplex species at variable pH. Six well resolved NOE signals in the [^{1}H,^{1}H]-NOESY spectra of metallo duplex samples were used to determine fraction minor duplex species (Supplementary Figure 30). DNA samples contained 0.76 mM duplex DNA and 2.28 mM of $Hg(ClO_4)_2$ (1.5 equiv Hg^{II} relative to mismatch) in aqueous buffer (200 mM $NaClO_4$, 50 mM cacodylic acid in H_2O / D_2O (9:1) at pH = 6 – 9). The data represent the mean value from six NOE signals and standard deviation is indicated by error bars. Source data are provided as a Source Data file.

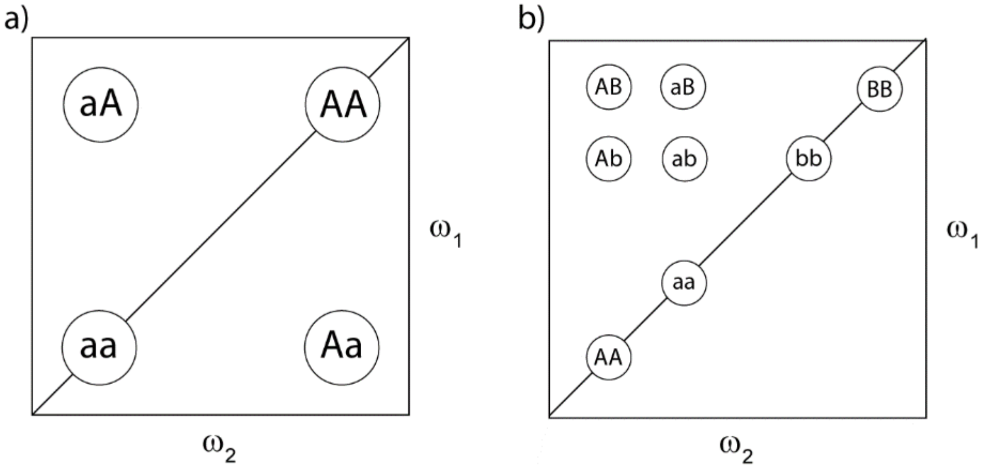

Supplementary Figure 36 | Definition of diagonal peaks, exchange cross peaks, and exchange-mediated NOE cross peaks. a) diagonal peaks (‘AA’ and ‘aa’) undergoing conformational interconversion to give exchange cross peaks (‘aA’ and ‘Aa’) and b) ‘AB’ and ‘ab’ NOE cross peaks undergoing conformational interconversion to give exchange-mediated NOE cross peaks ‘Ab’ and aB’.[17] Figure adapted from ref 17.

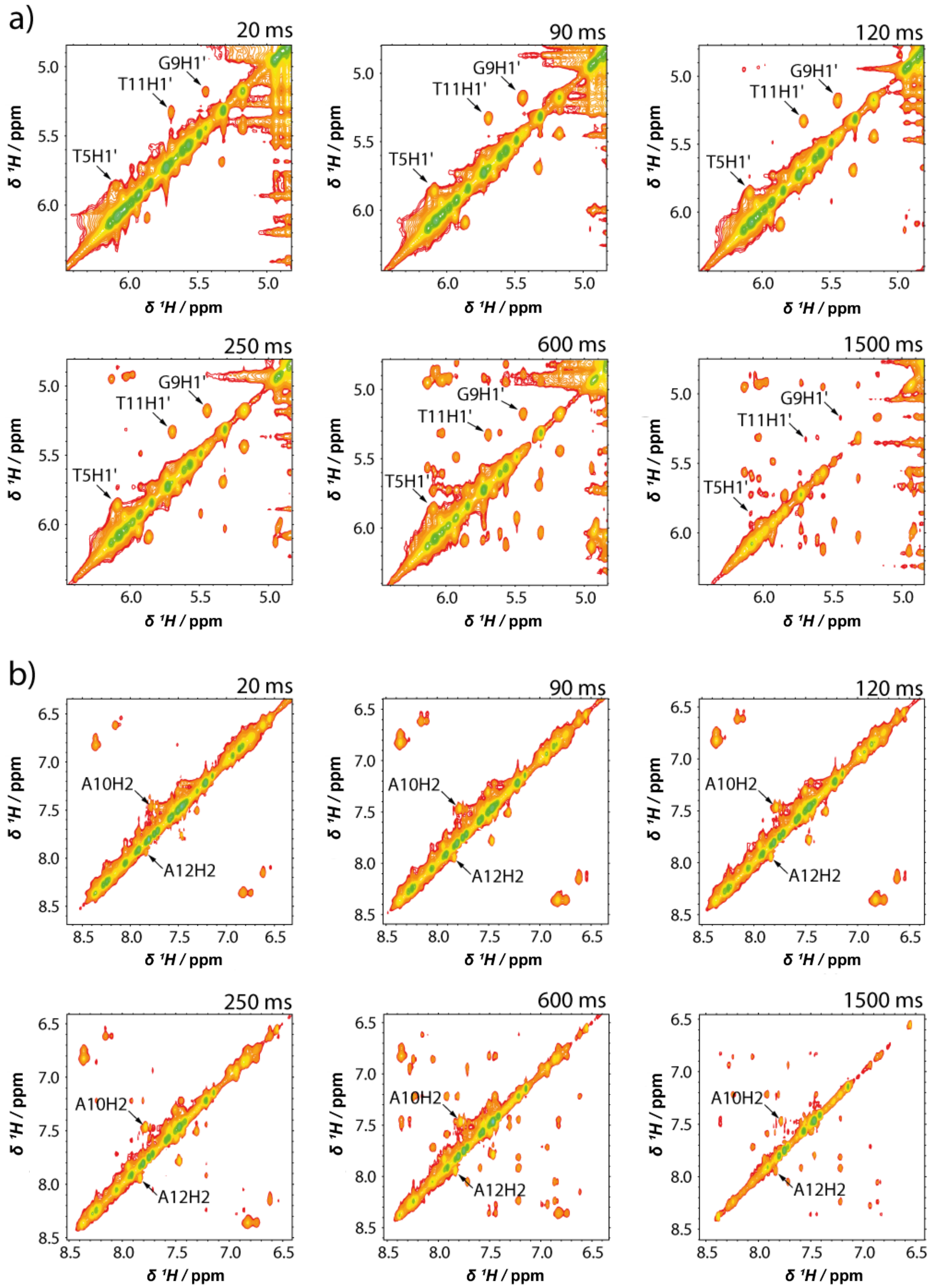

Supplementary Figure 37 | [^{1}H,^{1}H]-NOESY spectra with selected mixing times ranging from 20 ms to 1.5 s. Change of exchange cross peak intensities as a function of mixing time for a) H1' protons and b) H2 protons. The DNA sample contained 1 mM duplex DNA and 3 mM of ^{199}Hg-enriched $Hg(ClO_4)_2$ (1.5 equiv $^{199}Hg^{II}$ relative to mismatch) in aqueous buffer (200 mM $NaClO_4$, 50 mM cacodylic acid in H_2O / D_2O (9:1) at pH = 7.8).

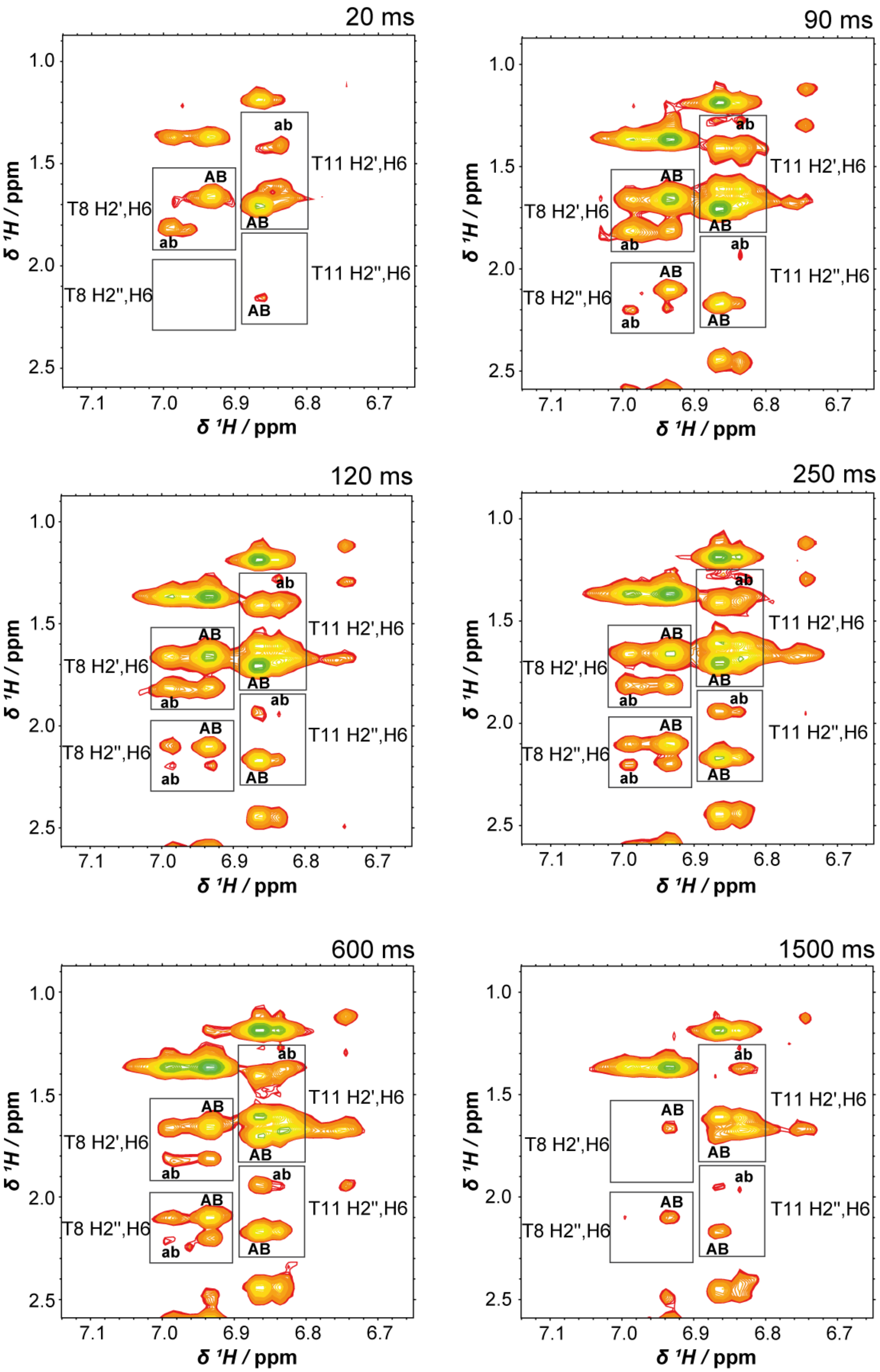

Supplementary Figure 38 | Changes of exchange-mediated NOE cross peak intensities as a function of mixing time. Exchange-mediated NOE cross peaks in the aromatic → H2'/H2'' region of the [^{1}H,^{1}H]-NOESY spectrum with selected mixing times ranging from 20 ms to 1.5 s. The DNA sample contained 1 mM duplex DNA and 3 mM of ^{199}Hg-enriched $Hg(ClO_4)_2$ (1.5 equiv $^{199}Hg^{II}$ relative to mismatch) in aqueous buffer (200 mM $NaClO_4$, 50 mM cacodylic acid in H_2O / D_2O (9:1) at pH = 7.8).

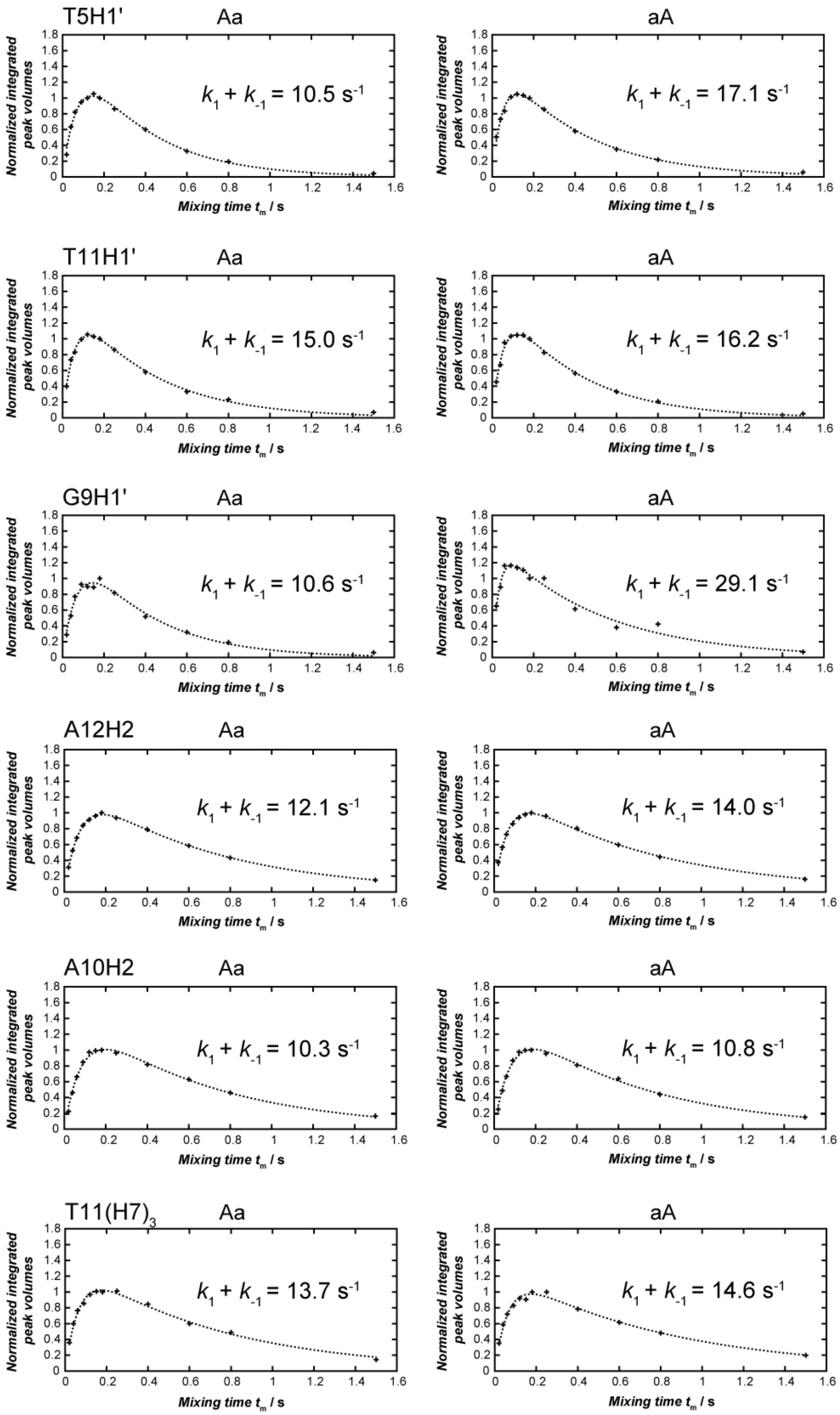

Supplementary Figure 39 | Sum of interconversion rate constants (k_1+k_{-1}). Sum of rate constants were determined by fit (Supplementary Equation 12) of change in cross peak intensities vs mixing time (t_m). The DNA sample contained 1 mM duplex DNA and 3 mM of ^{199}Hg-enriched $Hg(ClO_4)_2$ (1.5 equiv $^{199}Hg^{II}$ relative to mismatch) in aqueous buffer (200 mM $NaClO_4$, 50 mM cacodylic acid in H_2O / D_2O (9:1) at pH = 7.8). Source data are provided as a Source Data file.

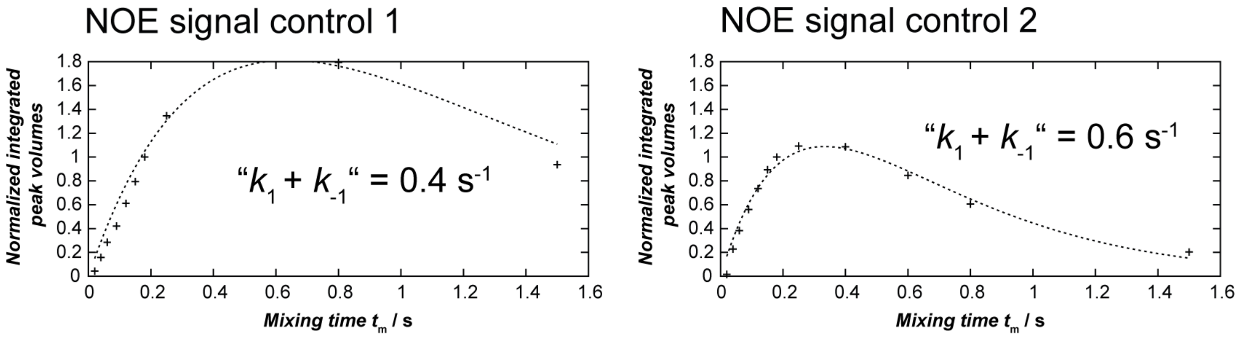

Supplementary Figure 40 | NOE signals A7H3',H1' processed equivalent to exchange signals as a control experiment. Both examples showed large deviations from the values obtained for exchange cross peaks. The DNA sample contained 1 mM duplex DNA and 3 mM of ^{199}Hg-enriched $Hg(ClO_4)_2$ (1.5 equiv $^{199}Hg^{II}$ relative to mismatch) in aqueous buffer (200 mM $NaClO_4$, 50 mM cacodylic acid in H_2O / D_2O (9:1) at pH = 7.8). Source data are provided as a Source Data file.

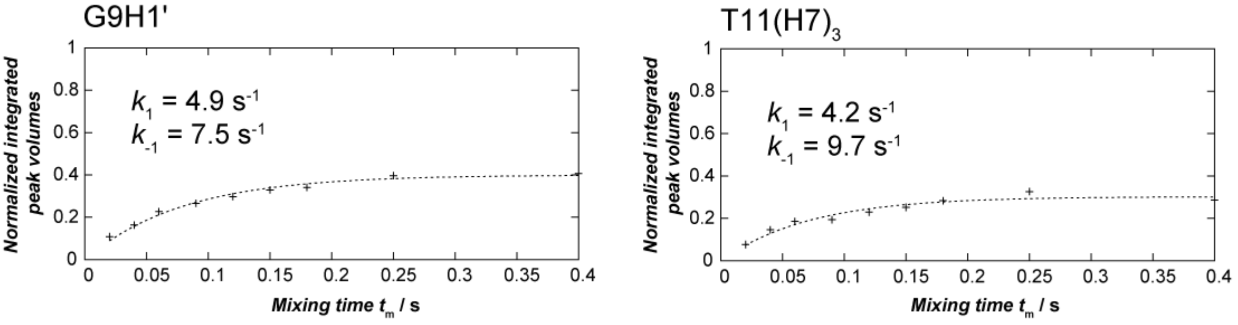

Supplementary Figure 41 | Exchange cross peak intensities normalized to the intensities of diagonal peaks. aA (t_m) / (aA (t_m) + AA (t_m)) is plotted as a function of mixing time (t_m). Rate constants of interconversion were determined by fit with Supplementary Equation 14. The DNA sample contained 1 mM duplex DNA and 3 mM of ^{199}Hg-enriched $Hg(ClO_4)_2$ (1.5 equiv $^{199}Hg^{II}$ relative to mismatch) in aqueous buffer (200 mM $NaClO_4$, 50 mM cacodylic acid in H_2O / D_2O (9:1) at pH = 7.8). Source data are provided as a Source Data file.

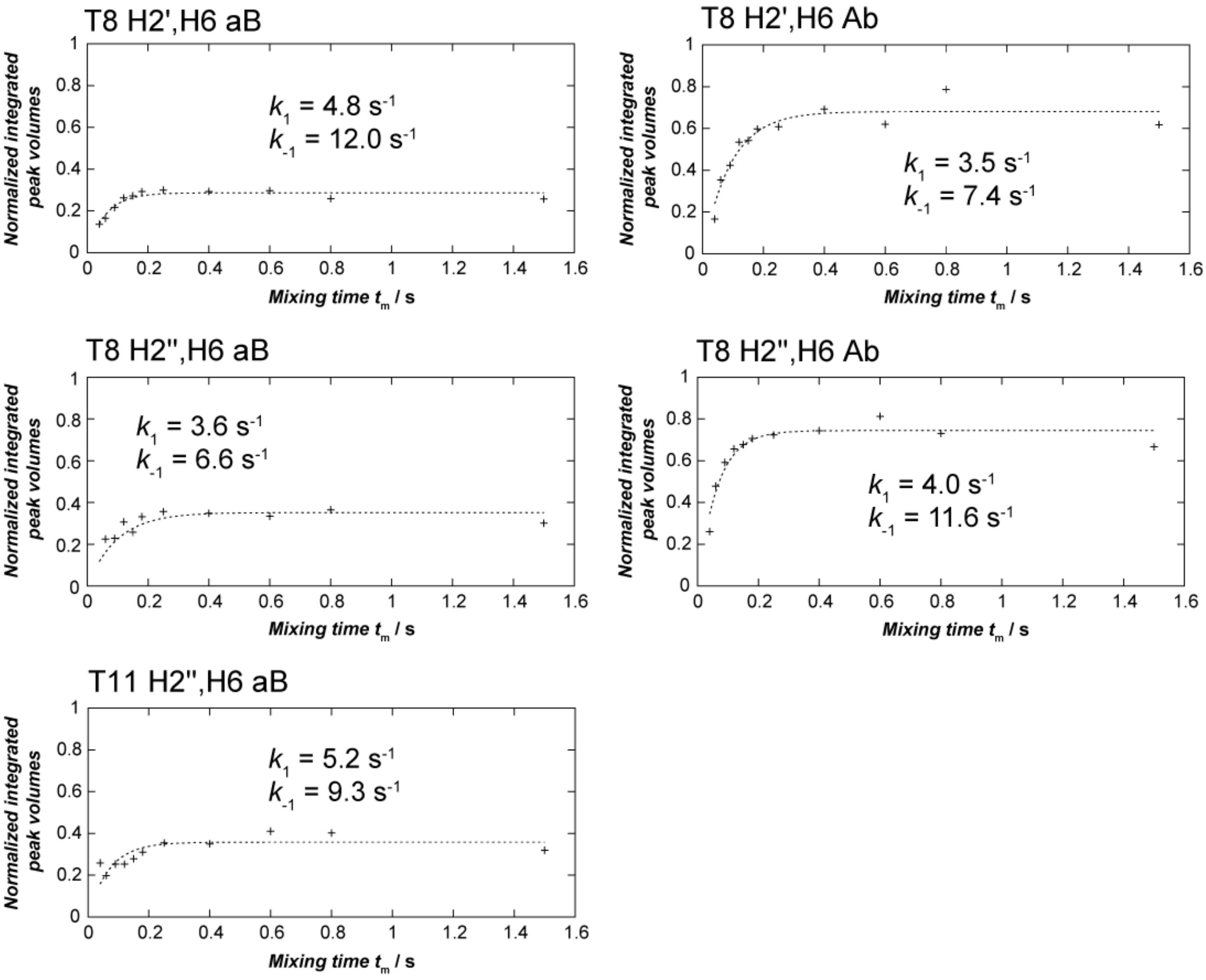

Supplementary Figure 42 | Normalized areas of exchange-mediated NOE cross peaks as a function of mixing time (t_m). Rate constants of interconversion were determined by fit with Supplementary Equation 18 ('aB') or Supplementary Equation 19 ('Ab'). The DNA sample contained 1 mM duplex DNA and 3 mM of ^{199}Hg-enriched $Hg(ClO_4)_2$ (1.5 equiv $^{199}Hg^{II}$ relative to mismatch) in aqueous buffer (200 mM $NaClO_4$, 50 mM cacodylic acid in H_2O / D_2O (9:1) at pH = 7.8). Source data are provided as a Source Data file.

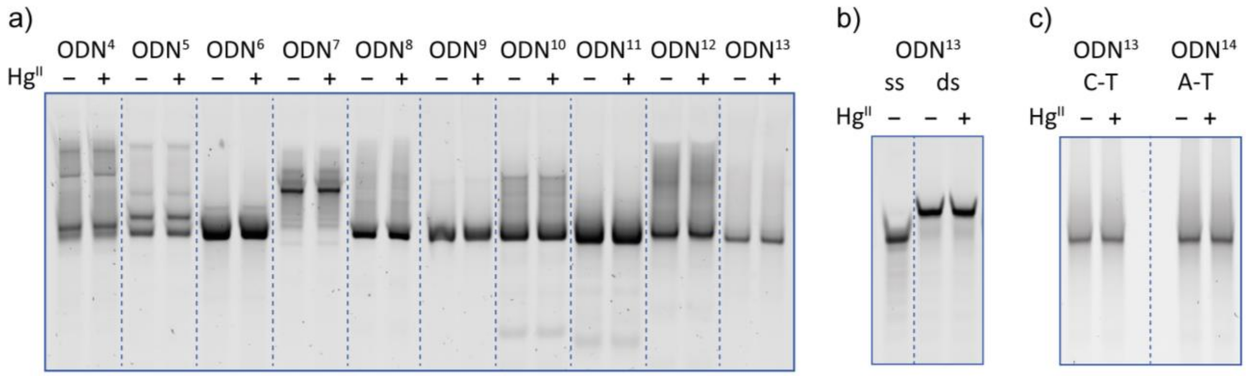

Supplementary Figure 43 | Native gel analysis of DNA repeat sequences in the presence and absence of Hg^{II}. a) intramolecular hairpin formation, b) ODN^{13} duplex DNA formation, and c) intramolecular hairpin formation of ODN^{13} containing C-T mismatches and ODN^{14} containing A-T base pairs in the absence or presence of Hg^{II}. All samples were prepared in aqueous buffer containing 200 mM $NaClO_4$ and 50 mM cacodylic acid (pH = 7.8). Sucrose (40 %, 5 µl) was given to pre-annealed hairpin or duplex DNA (5 µl, 5 pmol) prior to loading on gel. DNA samples measured in the presence of Hg^{II} were incubated with $Hg(ClO_4)_2$ (1.5 equiv relative to mismatch present) for 3 h prior to use. For oligonucleotide sequences see Supplementary Table 8. Source data are provided as a Source Data file.

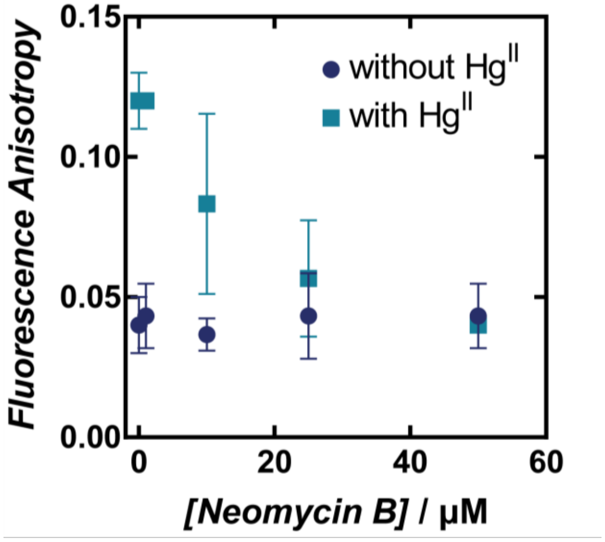

Supplementary Figure 44 | Changes in fluorescence anisotropy of Neo-BODIPY upon addition of unlabeled neomycin B. Unlabeled neomycin B was added to samples of ODN^{13} preincubated with Neo-BODIPY in the absence or presence of Hg^{II}. Samples in the absence of Hg^{II} (dark blue circles) contained 40 nM Neo-BODIPY, 0.6 µM ODN^{13} hairpin, and 0 – 50 µM unlabeled neomycin B. Samples in the presence of Hg^{II} (light blue squares) contained 40 nM Neo-BODIPY, 0.6 µM ODN^{13} hairpin, 13.5 µM $Hg(ClO_4)_2$ (1.5 equiv relative to mismatch present), and 0 – 50 µM unlabeled neomycin B and were incubated with Hg^{II} for 3 h prior to addition of Neo-BODIPY and unlabeled neomycin B. Samples were excited at λ_{ex} = 480 nm and fluorescence anisotropy was monitored at λ_{em} = 515 nm. All samples were prepared in aqueous buffer containing 200 mM $NaClO_4$ and 50 mM cacodylic acid (pH = 7.8). The data represent mean values and error bars represent standard deviation of three independent measurements. Source data are provided as a Source Data file.

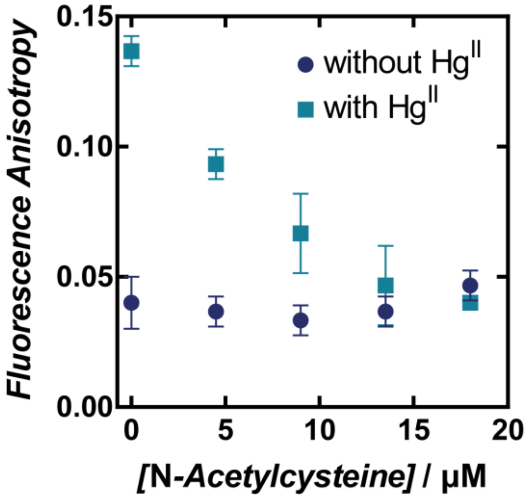

Supplementary Figure 45 | Changes in fluorescence anisotropy of Neo-BODIPY upon addition of *N*-acetylcysteine. *N*-acetylcysteine was added to samples of ODN[13] preincubated with Neo-BODIPY in the absence or presence of Hg^{II}. Samples in the absence of Hg^{II} (dark blue circles) contained 40 nM Neo-BODIPY, 0.6 µM ODN[13] hairpin, and 0 – 18 µM *N*-acetylcysteine. Samples in the presence of Hg^{II} (light blue squares) contained 40 nM Neo-BODIPY, 0.6 µM ODN[13] hairpin, 13.5 µM $Hg(ClO_4)_2$ (1.5 equiv relative to mismatch present), and 0 – 18 µM *N*-acetylcysteine and were incubated with Hg^{II} for 3 h prior to addition of Neo-BODIPY and *N*-acetylcysteine. Samples were excited at λ_{ex} = 480 nm and fluorescence anisotropy was monitored at λ_{em} = 515 nm. All samples were prepared in aqueous buffer containing 200 mM $NaClO_4$ and 50 mM cacodylic acid (pH = 7.8). The data represent mean values and error bars represent standard deviation of three independent measurements. Source data are provided as a Source Data file.

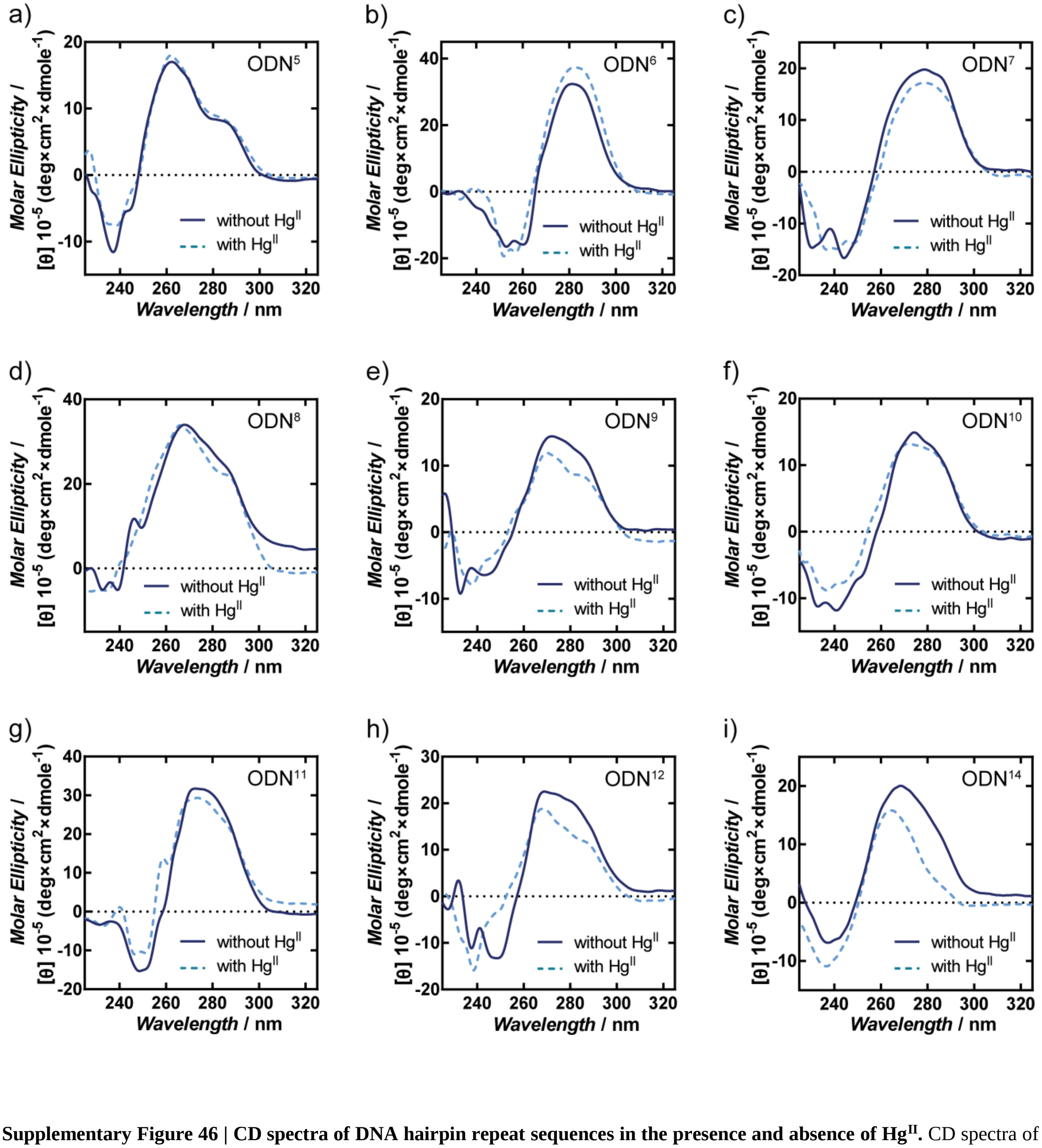

Supplementary Figure 46 | CD spectra of DNA hairpin repeat sequences in the presence and absence of Hg^{II}. CD spectra of a) ODN^5, b) ODN^6, c) ODN^7, d) ODN^8, e) ODN^9, f) ODN^{10}, g) ODN^{11}, h) ODN^{12}, and i) ODN^{14} in the presence (dashed, light blue) and absence (solid, dark blue) of 1.5 equiv Hg^{II}. Samples in the absence of Hg^{II} contained 1 μM hairpin DNA. Samples in the presence of Hg^{II} contained 1 μM hairpin DNA and were incubated with 1.5 equiv $Hg(ClO_4)_2$ (equiv relative to mismatch present) for 3 h prior to use. All samples were prepared in an aqueous buffer containing 200 mM $NaClO_4$ and 50 mM cacodylic acid (pH = 7.8).

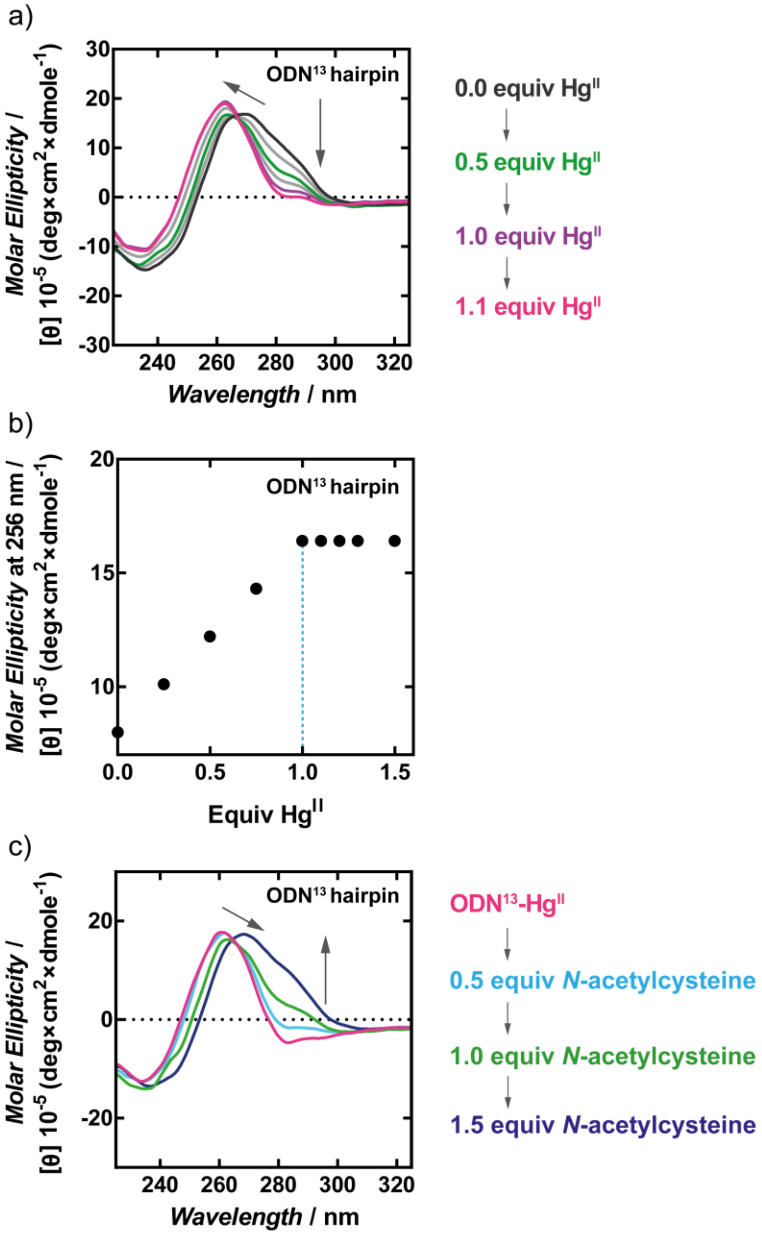

Supplementary Figure 47 | Stoichiometric Hg^{II} binding to ODN^{13} and reversibility of B-A transition by *N*-acetylcysteine. a) Binding of Hg^{II} to C-T mismatches induces B- to A-form helical transition of ODN^{13} hairpin according to changes in CD spectra. b) 1:1 stoichiometric binding of Hg^{II} to the number of C-T mismatches present in ODN^{13} hairpin according to changes in molar ellipticity at 256 nm. Equiv of Hg^{II} are given relative to mismatch present. c) Reverse A- to B-form transition upon addition of *N*-acetylcysteine. ODN^{13} hairpin (1 μM) was preincubated with $Hg(ClO_4)_2$ (22.5 μM, 1.5 equiv relative to mismatch) for 10 min prior to addition of *N*-acetylcysteine. All samples contained ODN^{13} hairpin (1 μM) in aqueous buffer (200 mM $NaClO_4$ and 50 mM cacodylic acid (pH = 7.8)). CD spectra were recorded after 10 min incubation with aliquots of $Hg(ClO_4)_2$ (a) or *N*-acetylcysteine (c). Source data are provided as a Source Data file.

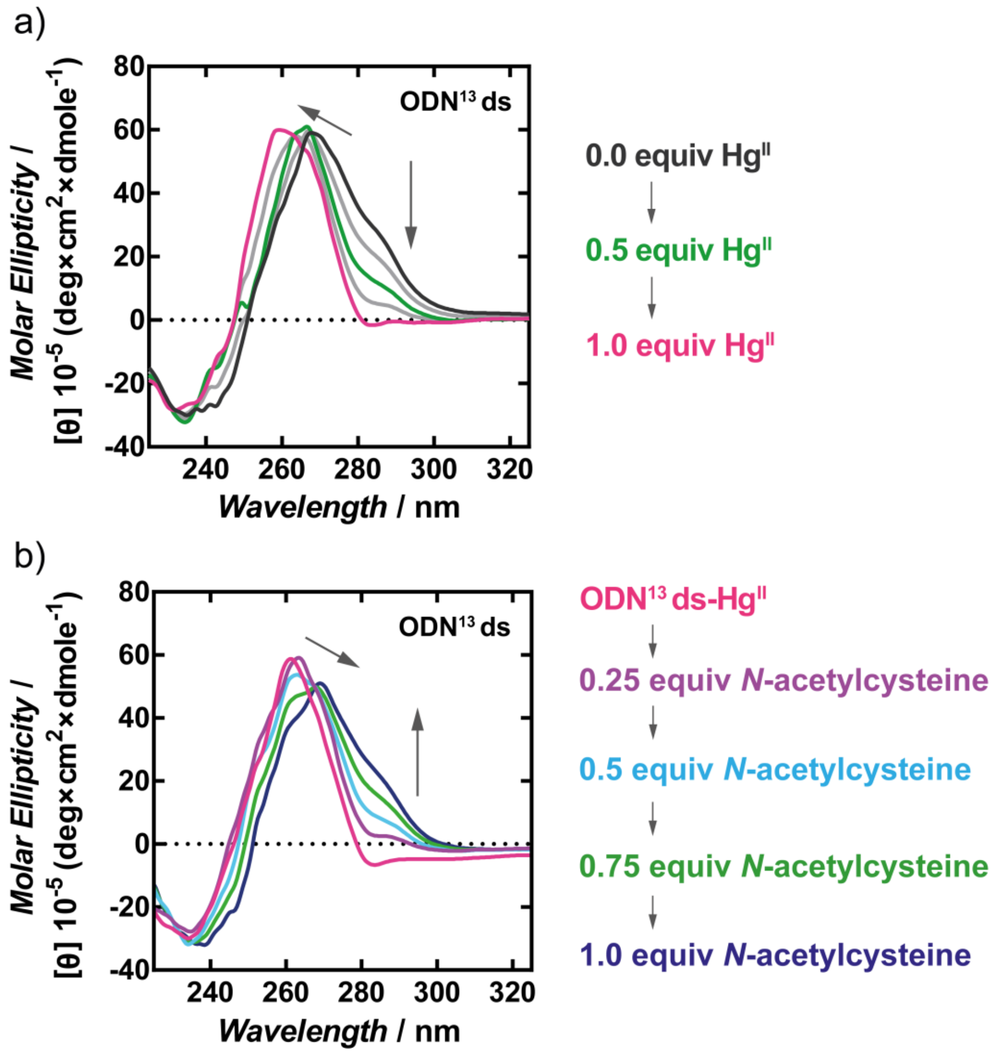

Supplementary Figure 48 | Stoichiometric Hg^{II} binding to ODN^{13} ds and reversibility of B-A transition by *N*-acetylcysteine. a) Stoichiometric binding of Hg^{II} to C-T mismatches induces B- to A-form helical transition of ODN^{13} ds according to changes in CD spectra. b) Reverse A- to B-form transition upon addition of *N*-acetylcysteine. ODN^{13} duplex (3 μM) was preincubated with $Hg(ClO_4)_2$ (45 μM, 1 equiv relative to mismatch present) for 10 min prior to addition of *N*-acetylcysteine. All samples contained ODN^{13} ds (3 μM) in aqueous buffer (200 mM $NaClO_4$ and 50 mM cacodylic acid (pH = 7.8)). CD spectra were recorded after 10 min incubation with aliquots of $Hg(ClO_4)_2$ (a) or *N*-acetylcysteine (b).

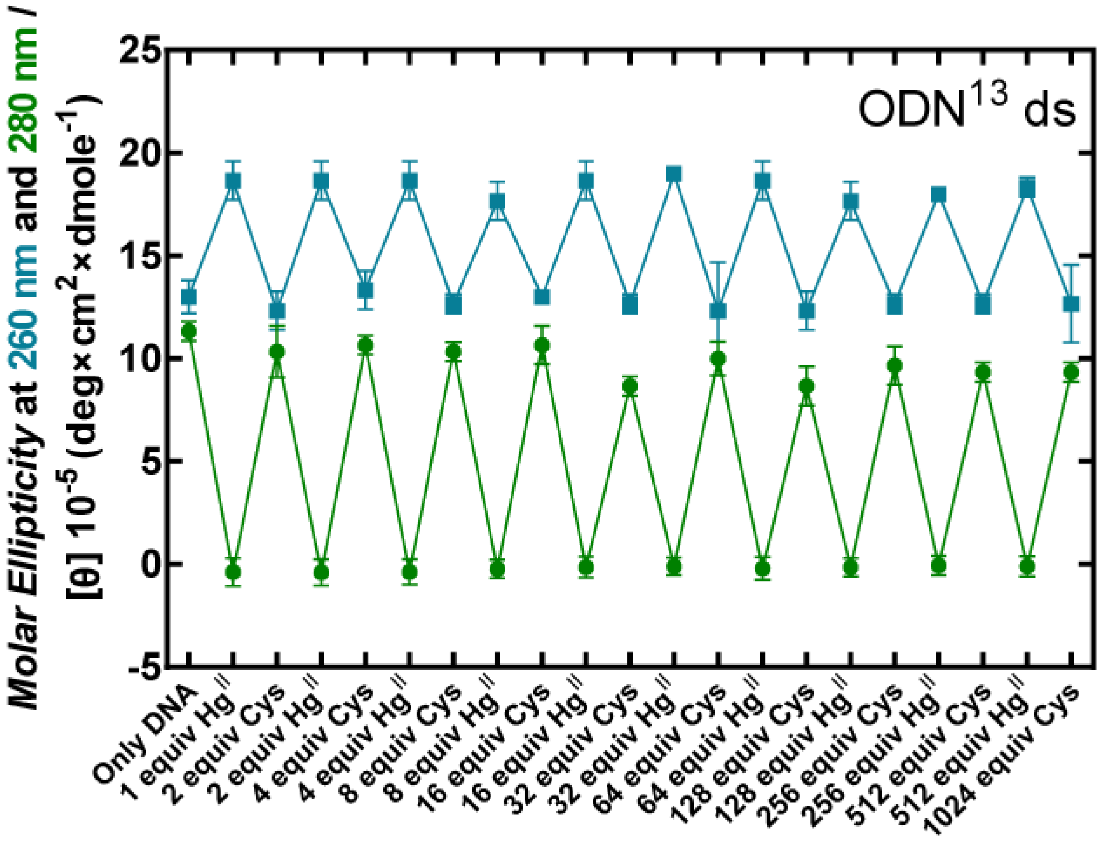

Supplementary Figure 49 | Helical switching cycle from B- to A-form and A- to B-form. Reversibility of A/B-form helical switching by the addition of Hg^{II} and *N*-acetylcysteine in alternating order according to changes in molar ellipticity at 260 nm (blue) and 280 nm (green). Samples contained ODN^{13} ds (1 µM) in aqueous buffer (200 mM $NaClO_4$ and 50 mM cacodylic acid (pH = 7.8)). Samples were incubated with $Hg(ClO_4)_2$ or *N*-acetylcysteine for 30 sec prior to measuring. Equiv of Hg^{II} and *N*-acetylcysteine are given relative to mismatch present. The data represent mean values and error bars represent standard deviation of three independent measurements. Source data are provided as a Source Data file.

Supplementary Tables

Supplementary Table 1: Names and Sequences of Duplex Oligonucleotides Used For T_m measurements and NMR spectroscopy. [a]

name		duplex sequence	ε_{260}
ODN[1]	"C-T"	`5'-CG T`**`C`**`T CAT GA`**`T`**` ACG-3'`<br>`3'-GC A`**`T`**`A GTA CT`**`C`**` TGC-5'`	133000 $M^{-1}cm^{-1}$
ODN[1]*	"C*-T*"	`5'-CG T`**`C*`**`T CAT GA`**`T*`**` ACG-3'`<br>`3'-GC A`**`T*`**`A GTA CT`**`C*`**` TGC-5'`	133000 $M^{-1}cm^{-1}$
ODN[2]	"T-T"	`5'-CG T`**`T`**`T CAT GA`**`T`**` ACG-3'`<br>`3'-GC A`**`T`**`A GTA CT`**`T`**` TGC-5'`	133900 $M^{-1}cm^{-1}$
ODN[3]	"G-T"	`5'-CG T`**`G`**`T CAT GA`**`T`**` ACG-3'`<br>`3'-GC A`**`T`**`A GTA CT`**`G`**` TGC-5'`	136500 $M^{-1}cm^{-1}$

[a] Bold bases indicate C-T mismatches. Stars indicate ^{15}N-labeled residues (**C*** = ^{15}N-labeled C, **T*** = ^{15}N-labeled T).

Supplementary Table 2: Imino-Proton Chemical Shift Values (ppm) of ODN[1]* "C*-T*" with and without Hg^{II} at 4 °C.[a]

Imino-proton signal	ppm in the absence of Hg^{II}	ppm in the presence of Hg^{II}
G28	12.75	12.79 (+0.04)
G2	12.64	12.65 (+0.01)
T3	13.64	13.45 (-0.19)
T25	10.86 (*d*, ^{1}J ^{1}H,^{15}N = 88 Hz)	-
T5	14.33	13.26 (-1.07)
G23	11.91	12.13 (+0.22)
T22	13.15	13.17 (+0.02)

[a] Values in brackets represent chemical shift changes upon addition of Hg^{II}. The DNA sample contained 0.5 mM duplex DNA in aqueous buffer (200 mM $NaClO_4$, 50 mM cacodylic acid in H_2O / D_2O (9:1) at pH = 7.8) in the absence and the presence of 1.5 mM of $Hg(ClO_4)_2$ (1.5 equiv relative to mismatch).

Supplementary Table 3: ^{15}N-Chemical Shift Values (ppm) of ODN^{1*} "C*-T*" with and without Hg^{II} at 25 °C.[a]

^{15}N signal	ppm in the absence of Hg^{II}	ppm in the presence of Hg^{II}
N1 (T)	142.6	N1^{major}: 142.7 (+0.1)
		N1^{minor}: 146.4 (+3.8)
N3 (T)	155.2	N3^{major}: 184.3 (+29.1)
		N3^{minor}: 183.1 (+27.9)
N1 (C)	152.3	N1^{major}: 155.7 (+3.4)
		N1^{minor}: 152.5 (+0.2)
N3 (C)	200.0	N3^{major}: 207.2 (+7.2)
		N3^{minor}: 207.2 (+7.2)
NH_2 (C)	95.9	(N4)NH_2^{major}: 96.7 (+0.8)
		(N4)NH^{minor}: 124.1 (+28.2)

[a] Values in brackets represent chemical shift changes upon addition of Hg^{II}. The DNA sample contained 1 mM duplex DNA and 3 mM of $Hg(ClO_4)_2$ (1.5 equiv Hg^{II} relative to mismatch) in aqueous buffer (200 mM $NaClO_4$, 50 mM cacodylic acid in H_2O / D_2O (9:1) at pH = 7.8).

Supplementary Table 4: ^{15}N-Chemical Shift Values (ppm) and ^{1}J ^{15}N,^{199}Hg coupling constants of ODN^{1*} "C*-T*" with and without Hg^{II} at 4 °C.[a]

^{15}N- signal	ppm in the absence of Hg^{II}	ppm in the presence of Hg^{II}	^{1}J ^{15}N,^{199}Hg
N1 (T)	142.3	N1^{major}: 142.2 (-0.1)	-
		N1^{minor}: 146.4 (+4.1)	-
N3 (T)	155.1	N3^{major}: 184.0 (+28.9)	1095 Hz
		N3^{minor}: 182.8 (+27.7)	n. d.
N1 (C)	151.6	N1^{major}: 155.5 (+3.9)	-
		N1^{minor}: 152.1 (+0.5)	-
N3 (C)	199.4	N3^{major}: 206.9 (+7.5)	114 Hz
		N3^{minor}: 206.9 (+7.5)	-
NH_2 (C)	95.9	(N4)NH_2^{major}: 96.9 (+1.0)	-
		(N4)NH^{minor}: 124.2 (+28.3)	1063 Hz

[a] Values in brackets represent chemical shift changes upon addition of Hg^{II}. The DNA sample contained 1 mM duplex DNA and 3 mM of ^{199}Hg-enriched $Hg(ClO_4)_2$ (1.5 equiv Hg^{II} relative to mismatch) in aqueous buffer (200 mM $NaClO_4$, 50 mM cacodylic acid in H_2O / D_2O (9:1) at pH = 7.8).

Supplementary Table 5: ^{3}J $^{1}H,^{1}H$ Coupling Constants of the major duplex species.[a]

Residue	H1',H2'	H1',H2"	H2',H2"
C1	7.8	6.9	13.8
G2	9.2	6.6	13.2
T3	8.6	6.9	13.7
C4	6.5	6.6	14.6
T5	n. d.	n. d.	n. d.
C6	8.4	7.1	13.5
A7	9	6.6	14.1
T8	n. d.	n. d.	n. d.
G9	n. d.	n. d.	n. d.
A10	n. d.	n. d.	n. d.
T11	9.3	6.9	14.9
A12	n. d.	n. d.	n. d.
C13	8.3	6.6	13.8
G14	8.4	6.6	13.9
S-type (C2'-endo) [4]	9.5	5.8	14.1
N-type (C3'-endo) [4]	1.5	7.7	14.1

[a] The DNA sample contained 0.4 mM duplex DNA (ODN[1] "C-T") and 1.2 mM Hg^{II} (1.5 equiv Hg^{II} relative to mismatch) in an aqueous solution of $NaClO_4$ (50 mM, D_2O, pD = 7.75) and was measured at 25 °C. n.d. = not determined.

Supplementary Table 6: Frequency of C2'-Endo, O4'-Endo, and C1'-Exo Sugar Pucker Conformation.[a]

Residue	C2'-endo (%)			O4'-endo (%)			C1'-exo (%)		
	major	minor	apo	major	minor	apo	major	minor	apo
C1	50	5	10	0	0	0	0	0	0
G2	100	100	100	0	0	0	0	0	0
T3	50	35	60	0	0	0	50	65	40
C4	0	0	0	65	100	80	10	0	20
T5	10	0	0	40	55	25	50	45	75
C6	0	0	0	75	80	45	25	20	55
A7	95	95	100	0	0	0	5	0	0
T8	0	0	0	100	90	10	0	10	90
G9	95	100	100	5	0	0	0	0	0
A10	75	90	0	5	0	70	20	10	30
T11	0	0	0	100	65	5	0	5	0
A12	25	45	0	15	30	95	55	25	5
C13	0	0	0	70	100	75	30	0	25
G14	45	40	25	35	35	60	10	25	15

[a] % represent percentage of sugar pucker in the 20 lowest energy conformations of major-, minor-, and apo duplex species. Sugar pucker conformations were determined using Curves+.[7] A single C3'-endo sugar pucker was observed in one of the 20 models of the major duplex. Otherwise no C3'-endo sugar puckers were observed.

Supplementary Table 7: Rate Constants of Nucleobase-Metal-Nucleobase Isomerization and of Duplex Interconversion.[a]

	$k_1 + k_{-1}$ (s^{-1})	k_1 (s^{-1})	k_{-1} (s^{-1})
**[^{15}N,^{1}H]-HSQC exchange peaks**	11.2	3.5	7.7
**exchange cross peaks**			
**T5H1'**	10.5	n.d.	n.d.
**T11H1'**	15.0	n.d.	n.d.
**G9H1'**	10.6	4.9	7.5
**A12H2**	12.1	n.d.	n.d.
**A10H2**	10.3	n.d.	n.d.
**T11(H7)$_3$**	13.7	4.2	9.7
**mean ± standard deviation**	12.0 ± 1.8	4.6 ± 0.4	8.6 ± 1.1
**exchange-mediated NOE cross peaks**			
**T8 H2',H6**	n.d.	4.2	9.7
**T8 H2'',H6**	n.d.	3.8	9.1
**T11 H2'',H6**	n.d.	5.2	9.3
**mean ± standard deviation**	n. d.	4.4 ± 0.6	9.4 ± 0.2

[a] Rate constants of nucleobase-metal-nucleobase isomerization were determined by global fitting of integrated peak volumes versus delay time (t_d) using Supplementary Equations 6-9, SI. Sums of rate constants (k_1+k_{-1}) were determined by fitting exchange cross peak vs mixing time (t_m) with Supplementary Equation 12. Rate constants k_1 and k_{-1} were determined from normalized exchange cross peak areas vs mixing time (t_m) by fitting with Supplementary Equation 14, or from plot of exchange-mediated NOE cross peaks by fit with Supplementary Equation 18 or Supplementary Equation 19. Values for T8 H2',H6 and T8 H2",H6 were calculated as the mean of both exchange mediated NOE cross peaks. The DNA sample contained 0.5 mM duplex DNA and 1.5 equiv of $Hg(ClO_4)_2$ (nucleobase-metal-nucleobase isomerization) or 1 mM duplex DNA and 1.5 equiv of ^{199}Hg-enriched $Hg(ClO_4)_2$ (global helix interconversion) in aqueous buffer (200 mM $NaClO_4$, 50 mM cacodylic acid in H_2O / D_2O (9:1) at pH = 7.8) and spectra were measured at 25 °C. For spectra see Supplementary Figures 23 and 37-38. For fits see Figure 5a and Supplementary Figures 39-42. Equiv of Hg^{II} are given relative to mismatch.

Supplementary Table 8: Names and Sequences of 120-mer DNA Hairpin Small Library.[a]

ODN[4]	5'-GGCC**C**GGGC**C**CGGGCC**C**GGGC**C**CGGGCC**C**GGGC**C**CGGGCC**C**GGGC**C**CGGGCC**C**GGGCC***GCTA***<br>GGCCC**T**GGCCCG**T**GCCC**T**GGCCCG**T**GCCC**T**GGCCCG**T**GCCC**T**GGCCCG**T**GCCC**T**GGCC-3'
ODN[5]	5'-CCC**C**GGGCCC**C**GGGGCCCC**T**GGGCCC**C**GGGGCCCC**T**GGGCCC**C**GGGGCCCC**T**GGGCCC***GCTA***<br>GGGCCC**C**GGGGCCCC**T**GGGCCC**C**GGGGCCCC**T**GGGCCC**C**GGGGCCCC**T**GGGCCC**T**GGG-3'
ODN[6]	5'-GCG**T**GCG**T**GCG**T**GCG**T**GCG**T**GCG**T**GCG**T**GCG**T**GCG**T**GCG**T**GCG**T**GCG**T**GCG**T**GCG**T**GC***GCTA***<br>GC**C**CGC**C**CGC**C**CGC**C**CGC**C**CGC**C**CGC**C**CGC**C**CGC**C**CGC**C**CGC**C**CGC**C**CGC**C**CGC**C**CGC-3'
ODN[7]	5'-GGC**T**C**T**GGGC**T**C**T**GGGC**T**C**T**GGGC**T**C**T**GGGC**T**C**T**GGGC**T**C**T**GGGC**T**C**T**GGGC**T**C**T**GGC***GCTA***<br>GCC**C**G**C**GCCC**C**G**C**GCCC**C**G**C**GCCC**C**G**C**GCCC**C**G**C**GCCC**C**G**C**GCCC**C**G**C**GCCC**C**G**C**GCC-3'
ODN[8]	5'-CGG**T**GG**T**GCC**C**CCGG**T**GG**T**GCC**C**CCGG**T**GG**T**GCC**C**CCGG**T**GG**T**GCC**C**CCGG**T**GG**T**GCC***GCTA***<br>GGC**C**CC**C**CCGG**T**GGC**C**CC**C**CCGG**T**GGC**C**CC**C**CCGG**T**GGC**C**CC**C**CCGG**T**GGC**C**CC**C**CCG-3'
ODN[9]	5'-CGC**T**GGG**T**GG**T**CGC**T**GGG**T**GG**T**CGC**T**GGG**T**GG**T**CGC**T**GGG**T**GG**T**CGC**T**GGG**T**GG**T**CGC***GCTA***<br>GCG**C**CC**C**CCC**C**GCG**C**CC**C**CCC**C**GCG**C**CC**C**CCC**C**GCG**C**CC**C**CCC**C**GCG**C**CC**C**CCC**C**GCG-3'
ODN[10]	5'-CGC**C**GGG**T**GG**C**CGC**C**GGG**T**GG**C**CGC**C**GGG**T**GG**C**CGC**C**GGG**T**GG**C**CGC**C**GGG**T**GG**C**CGC***GCTA***<br>GCG**T**CC**C**CCC**T**GCG**T**CC**C**CCC**T**GCG**T**CC**C**CCC**T**GCG**T**CC**C**CCC**T**GCG**T**CC**C**CCC**T**GCG-3'
ODN[11]	5'-CGC**T**GGG**C**GG**T**CGC**T**GGG**C**GG**T**CGC**T**GGG**C**GG**T**CGC**T**GGG**C**GG**T**CGC**T**GGG**C**GG**T**CGC***GCTA***<br>GCG**C**CC**T**CCC**C**GCG**C**CC**T**CCC**C**GCG**C**CC**T**CCC**C**GCG**C**CC**T**CCC**C**GCG**C**CC**T**CCC**C**GCG-3'
ODN[12]	5'-GGC**T**CC**T**CGG**T**GGC**T**CC**T**CGG**T**GGC**T**CC**T**CGG**T**GGC**T**CC**T**CGG**T**GGC**T**CC**T**CGG**T**GGC***GCTA***<br>GCC**C**CCG**C**GG**C**GCC**C**CCG**C**GG**C**GCC**C**CCG**C**GG**C**GCC**C**CCG**C**GG**C**GCC**C**CCG**C**GG**C**GCC-3'
ODN[13]	5'-CGG**T**GG**T**CCC**T**CGG**T**GG**T**CCC**T**CGG**T**GG**T**CCC**T**CGG**T**GG**T**CCC**T**CGG**T**GG**T**CCC**T**CGC***GCTA***<br>GCG**C**GGG**C**CC**C**CCG**C**GGG**C**CC**C**CCG**C**GGG**C**CC**C**CCG**C**GGG**C**CC**C**CCG**C**GGG**C**CC**C**CCG-3'
ODN[13] ds	5'-CGG**T**GG**T**CCC**T**CGG**T**GG**T**CCC**T**CGG**T**GG**T**CCC**T**CGG**T**GG**T**CCC**T**CGG**T**GG**T**CCC**T**CGG-3'<br>3' GCC**C**CC**C**GGG**C**GCC**C**CC**C**GGG**C**GCC**C**CC**C**GGG**C**GCC**C**CC**C**GGG**C**GCC**C**CC**C**GGG**C**GCC-5'
ODN[14]	5'-CGG**T**GG**T**CCC**T**CGG**T**GG**T**CCC**T**CGG**T**GG**T**CCC**T**CGG**T**GG**T**CCC**T**CGG**T**GG**T**CCC**T**CGC***GCTA***<br>GCC**A**CC**A**GGG**A**GCC**A**CC**A**GGG**A**GCC**A**CC**A**GGG**A**GCC**A**CC**A**GGG**A**GCC**A**CC**A**GGG**A**GCC-3'

[a] Bold bases indicate C-T mismatches (ODN[4] – ODN[13]) or T-A base pairs (ODN[14]). Bold italic bases indicate loop regions.

Supplementary Methods

Synthesis and Folding of Duplex DNA

Unmodified oligonucleotides were purchased from Sigma-Aldrich as HPLC-purified sequences. ^{15}N-labeled cytosine and thymidine phosphoramidites were purchased from Cambridge Isotope Laboratories, Inc. ^{15}N-containing sequences were synthesized on a 1 μmol scale using a Bioautomation Mermade 4 DNA synthesizer using standard trityl-on procedure, except that 5 % dichloroacetic acid in CH_2Cl_2 was used for the deprotection step. For incorporation of ^{15}N-labeled nucleosides three coupling reactions were performed with prolonged coupling times (400 s). ^{15}N-containing oligonucleotides were purified using Glen-Pak cartridges purchased from Glen Research according to their procedure. Purified oligonucleotides were analyzed with MALDI-MS spectroscopy using THAP as matrix in a reflector-ion mode on a Bruker Ultraflextreme MALDI mass spectrometer. Unlabeled oligonucleotide was measured in parallel. In comparison to the unlabeled oligonucleotide, the ^{15}N-labeled oligonucleotide showed a mass elevation of 5 dalton (Supplementary Figure 1). Oligonucleotide stock solutions were prepared in deionized water and their concentrations were determined by absorbance at 260 nm using a molar extinction coefficient (ε_{260}) calculated using a nearest-neighbor model.[18,19] For calculated extinction coefficients see Supplementary Table 1. Double stranded oligonucleotides were prepared by diluting the self-complementary sequences in the indicated aqueous buffer and heating to 95 °C for 5 min, followed by slow cooling to room temperature over 4 h.

Melting Temperature Analysis (T_m) and CD Spectroscopy

Thermal denaturation temperatures of duplex DNA were determined by measuring the absorbance at 260 nm as a function of temperature in a 1 mm path length thermo-controlled strain-free quartz cuvette on a JASCO J-715 spectrometer equipped with a temperature control system. Solutions of pre-folded duplex DNA in aqueous buffer (200 mM $NaClO_4$, 50 mM cacodylic acid at pH = 7.0) were equilibrated at 4 °C for a minimum of 20 min and slowly ramped to 90 °C with 0.5 °C steps at a rate of 25 °C h^{-1}. The melting temperatures were determined from the obtained maximum after differentiation of the received curves on Spectra Manager. T_m values were calculated as the average from the heating and cooling curves and were measured in triplicate.

Circular dichroism spectra of pre-annealed duplex DNA (10 μM) were measured from 200 nm to 350 nm at 4 °C and 25 °C with a 2 nm band width with 0.1 nm steps at a scanning rate of 20 nm min^{-1} in 1 mm

path length thermo-controlled strain-free quartz cuvette on a JASCO J-715 spectrometer. Duplex DNA (10 μM) was formed by dissolving the self-complementary sequences in aqueous buffer (200 mM $NaClO_4$, 50 mM cacodylic acid at pH = 7.0), heating to 95 °C for 5 min, and slowly cooling to room temperature over 4 h. The pH was then adjusted to 7.9 by addition of a solution of NaOH. CD spectra of duplex DNA measured in the presence of Hg^{II} were incubated with 2.0 equiv of $Hg(ClO_4)_2$ for 3 h prior to use.[1]

Sample Preparation of Duplex DNA used for NMR studies

Duplex DNA (0.5 mM or 1.0 mM) was prepared by dissolving 1.0 mM or 2.0 mM of the self-complementary sequence in aqueous buffer (200 mM $NaClO_4$, 50 mM cacodylic acid in H_2O / D_2O (9:1) at pH = 7.0), heating to 95 °C for 5 min, and slowly cooling to room temperature over 4 h. For Hg^{II} titration experiments, aliquots of $Hg(ClO_4)_2$ were added, and NMR spectra were recorded after incubating each sample for 15 min at r.t. followed by 15 min at 4 °C. ^{15}N-labeled duplex DNA (0.5 mM or 1.0 mM) was prepared by dissolving 1.0 mM or 2.0 mM of the self-complementary sequence in an aqueous buffer (200 mM $NaClO_4$, 50 mM cacodylic acid in H_2O / D_2O (9:1) at pH = 7.0) and annealed as described above. The pH was adjusted to approximately pH = 7.8 by addition of an aqueous solution of NaOH. For solution structure determination, oligonucleotides were desalted, and duplex DNA (0.4 mM) was prepared by dissolving 0.8 mM of the self-complementary sequence in an aqueous solution of $NaClO_4$ (50 mM, 90:10 H_2O / D_2O) and the pH was adjusted to 7.75 by addition of an aqueous solution of NaOH. The samples were annealed as described above and if necessary the pH was readjusted to pH = 7.75. Samples measured in D_2O were prepared in an aqueous solution of $NaClO_4$ (50 mM) and the pH was adjusted to 7.75 by addition of an aqueous solution of NaOH and annealed as described above. The samples were lyophilized, dissolved in 99.9% D_2O and the pD was adjusted to 7.35 by addition of a solution of NaOD in 99.9 % D_2O. DNA samples used for structure determination measurements and containing Hg^{II} were treated with Chelex-100 (BIO-RAD) for 10 min after addition of Hg^{II} (1.5 equiv with respect to mismatch) to remove unspecific Hg^{II} coordination.[20] Samples measured at 4 °C were equilibrated at 4 °C for 15 min prior to measuring.

NMR Spectra Measurements

^{1}H NMR spectra were recorded on a Bruker Avance II 500 MHz spectrometer equipped with a TXI *z*-axis gradient probe head using excitation sculpting for water suppression. Proton chemical shifts were referenced to the water line at 4.70 ppm at the given temperature. The spectra were processed with a line broadening factor of 10 Hz. [^{1}H,^{1}H]-NOESY spectra for unlabeled oligonucleotides in 200 mM $NaClO_4$, 50

mM cacodylic acid in H_2O / D_2O (9:1) at pH = 7.0 and for ^{15}N-labeled oligonucleotides (200 mM $NaClO_4$, 50 mM cacodylic acid in H_2O / D_2O (9:1) at pH = 7.8) were recorded on a Bruker Avance 600 MHz spectrometer equipped with a TCI *z*-axis gradient CryoProbe at 4 °C with mixing times of 100 ms and 120 ms, respectively. Proton chemical shifts were referenced to the water line at 4.70 ppm at the given temperature. 1D ^{15}N NMR spectra were recorded on a Bruker Avance II 500 MHz spectrometer equipped with a BBO *z*-axis gradient CryoProbe at 4 °C or 25 °C using either inverse gated or no proton decoupling. An inter-scan delay of 1 s was used with a nominal 30° pulse for excitation and the pulse sequence employed a gradient spin echo of 2.5 ms duration prior to acquisition in order to get rid of the strong background signal. The total experiment time was typically on the order of 72 h. ^{15}N chemical shifts were indirectly referenced against ^1H using $\Xi = 0.101329118$.[21] The spectra were processed with a line-broadening factor of 10 Hz. ^1J [^{15}N,^1H]-HSQC spectra were recorded on a Bruker Avance II 500 MHz spectrometer equipped with a BBO *z*-axis gradient CryoProbe at 4 °C. Proton chemical shifts were referenced to the water line at 4.70 ppm at 4 °C and ^{15}N chemical shifts were indirectly referenced against ^1H using $\Xi = 0.101329118$.[21] The INEPT time in the ^1J [^{15}N,^1H]-HSQC's was set to select for a 90 Hz coupling. z-z exchange ^1J [^{15}N,^1H]-HSQC spectra containing a delay time before reverse INEPT were recorded on a Bruker Avance 600 MHz spectrometer equipped with TCI *z*-axis gradient CryoProbe at 25° C. Long-range [^{15}N,^1H]-HSQC spectra were recorded on a Bruker Avance 600 MHz spectrometer equipped with TCI *z*-axis gradient CryoProbe at 4 °C or on a Bruker Avance 700 MHz spectrometer equipped with TXI *z*-axis gradient CryoProbe at 25 °C. The INEPT time was set to select for a 20 Hz coupling. In order to enhance sensitivity, band-selective long-range [^{15}N,^1H]-HSQCs were measured in some instances in which the 180° ^{15}N pulses in both INEPT blocks were replaced by band-selective pulses with a minimal excitation to the ^{15}N nuclei of the NH_2 and NH resonances. The INEPT time was set to 25 Hz coupling. Water flip-back pulses together with the WATERGATE method were used for water suppression. For solution structure determination non-exchangeable resonances were assigned from [^1H,^1H]-NOESY spectra (4 °C and 25 °C and mixing times of 60 ms and 250 ms), [^1H,^1H]-total correlation spectra (TOCSY) (4 °C, 50 ms mixing time), and [^1H,^1H]-correlation spectra (COSY) in D_2O, recorded either on a Bruker Avance 600 MHz spectrometer equipped with a TCI *z*-axis gradient CryoProbe or on a Bruker Avance 700 MHz spectrometer equipped with TXI *z*-axis gradient CryoProbe. Exchangeable protons were assigned from [^1H,^1H]-NOESY spectra (4 °C, 150 ms mixing time) recorded on a Bruker Avance 600 MHz spectrometer equipped with a TCI *z*-axis gradient CryoProbe in H_2O / D_2O (9:1). ^{31}P spectra were recorded on a Bruker Avance II 500 MHz spectrometer equipped with a BBO *z*-axis gradient CryoProbe at 25 °C. [^1H,^1H]-Exclusive correlation spectra (E.COSY)[22] were recorded on a Bruker Avance 600 MHz spectrometer equipped with a TCI *z*-axis gradient CryoProbe.

NMR Solution Structure Calculations

The integrated peak volumes from the [^{1}H,^{1}H]-NOESY spectrum measured at 25 °C with a mixing time of 250 ms were calibrated to distances using CALIBA macro in DYANA.[23] The NOE signals were grouped into four categories: (i) strong (1.8 – 3.0 Å), (ii) medium (1.8 – 4.5 Å), (iii) weak (3.0 – 6.0 Å), and very weak (4.0 – 7.0 Å). Structure calculations were performed with XPLOR-NIH 2.46 using standard implemented force field parameters.[24,25] For C-T mismatch- and C-Hg^{II}-T base pair residues no backbone dihedral angle-, backbone torsion angle-, or sugar pucker restraints were applied. For the other residues the following angle restraints were used: Based on ^{31}P NMR, α and ζ backbone dihedral angles were restrained to α = -70 ° ± 20 ° and ζ = -85 ° ± 20 °,[26] respectively, to exclude trans-conformation (Supplementary Figure 16).[27] Based on H1',H2', H1',H2", and H1',H3' cross peaks in [^{1}H,^{1}H]-NOESY spectra and cross peaks in [^{1}H,^{1}H]-COSY and [^{1}H,^{1}H]-TOCSY spectra, the torsion angles *ß* (180 ° ± 20 °)[26], γ (60 ° ± 20 °)[28], δ (140 ° ± 25 °)[28], and ε (-170 ° ± 20 °)[29] were set to B-form values. Glycosidic angles χ were restrained to -120 ± 20 Å (*anti*-conformation).[26] Planarity and H-bond distance restraints were used for all residues, except for C-T mismatch- and C-Hg^{II}-T base pair residues. Hg^{II} ions were included as a 2+ positive charge. For C-Hg^{II}-T base pairs in the major duplex species, the following constraints were included based on crystal structures of similar base pairs: (i) distance N3(C)–N3(T) = 4.1 ± 0.3 Å;[30] (ii) bond length N3(C)–Hg^{II} = 2.04 Å, bond length N3(T)–Hg^{II} = 2.04 Å;[30] (iii) angle N3(T)–Hg^{II}–N3(C) = 180 °,[31] angle thymidine C2/C4–N3–Hg^{II} = 117 °,[32] angle cytosine C2–N3–Hg^{II} = 113.4 °,[33] angle cytosine C4–N3–Hg^{II} = 126.1 °.[33] For C-Hg^{II}-T base pairs in the minor duplex species, the following constraints were included based on a crystal structure of a C-Hg^{II}-T-containing duplex DNA:[34] (i) distance N4(C)- N3(T) = 4.3 ± 0.3 Å; (ii) bond length N4(C)-Hg^{II} = 2.13 Å, bond length N3(T)-Hg^{II} = 2.13 Å; (iii) angle N3(T)–Hg^{II}–N4(C) = 180 °, angle thymidine C2/C4–N3–Hg^{II} = 116 °, angle cytosine C4–N4–Hg^{II} = 118 °, angle H–N4–Hg^{II} = 120 °. H3', H4', H5', and H5" sugar protons were only assigned for the major duplex species. For the minor duplex, same NOE-coupling patterns and NOE-cross peak intensities for the above mentioned sugar proton resonances were taken as for the major species for the structure calculation. Given the experimental C_2 symmetry observed in all NMR spectra, a non-crystallographic symmetry term was introduced for the calculations.

Starting from an extended structure generated based on the sequence of nucleoside residues, 2000 structures were calculated based on NOE-, dihedral-, planarity-, and H-bond distance restraints using simulated annealing. The 20 lowest energy structures were selected and used for further refinement using additional RAMA and ORIE database terms. 200 refined structures were calculated and the 20 lowest energy structures were visualized and analyzed. For the apo duplex species model #7 was replaced with #21 due to a bond angle outlier in model #7. The representative model (apo: model 1, major: model 1, minor: model 6) were chosen based on fewest outliers in the geometric quality criteria (standard geometry (bond lengths,

bond angles, chirality, and planarity)) and too-close contacts between non-bonded atoms with unfavorable steric overlap of van der Waals shells. Root mean square deviations (r.m.s.d.) were calculated using MOLMOL[35] and duplexes were visualized using PyMOL.[6] Base-pair parameters were determined using Curves+[7] or 3DNA.[11,12] Structure coordinates and NMR chemical shift assignments are deposited in the Protein Data Bank as 6RLS (apo species), 6FY6 (major species), and 6FY7 (minor species).

HR-MS of $Hg(ClO_4)_2$

Ethylenediamine tetraacetic acid disodium salt (Na_2EDTA) (60 μM) was added to ODN[1]* "C*-T*" (4 μM) containing 12 μM ^{199}Hg-enriched $Hg(ClO_4)_2$. After incubating for 20 min, high-resolution electrospray mass spectra (HR-ESI-MS) of the Hg^{II}-$EDTA^{2-}$ complex were recorded on a Bruker maXis QTOF-MS. As control experiments the HR-ESI-MS of natural abundance $Hg(ClO_4)_2$ and ^{199}Hg-enriched $Hg(ClO_4)_2$ without DNA were recorded after incubation with EDTA. Samples were measured via continuous flow injection with a flow rate of 3 μl min^{-1}. The mass spectrometer was calibrated between *m/z* 118 and 2721 using an Agilent ESI-L low concentration tuning mix solution at a resolution of 20'000 and a mass accuracy below 2 ppm.

Calculation of Pseudorotation Phase Angle

The pseudorotational phase angle (P) was determined from a plot of ^3J H1',H2' as a function of P.[2] The dihedral angle $\Phi_{1'2'}$ was calculated according to the Karplus relation.[3] (Supplementary Equation 1)

$$J = 10.2 \cos^2 \Phi - 0.8 \cos \Phi \qquad \text{Supplementary Equation 1}$$

Rate Constants of Nucleobase-Metal-Nucleobase Isomerization

To investigate dynamic changes in local metal ion coordination, we measured [^{15}N,^{1}H]-HSQC with a delay before reverse INEPT. To determine rate constants of interconversion, the decreases of cross peaks and increases of exchange cross peaks were monitored as a function of delay time (t_d) at 25 °C (Supplementary Figure 23). The integrals of the cross peaks (NH_2 and NH) and exchange peaks (NH_2' and NH') were taken, normalized to one of the NH_2^{major} signals (6.81/96.71 ppm) from the [^{15}N,^{1}H]-HSQC spectrum with a delay time (t_d) = 100 ms, and plotted as a function of delay time (t_d). The rate constants of interconversion were determined by global fitting of integrated peak volumes versus delay times (t_d) using Supplementary Equations 6-9,[36] respectively, under the assumption that all proton- and nitrogen atoms have the same relaxation rate (R_1) and that the NH proton is converted equally to both NH_2 protons during the exchange.[36] A global fit was used, where R_1, k_1, and k_{-1} was constrained to be equal for all four curves.

The decay of NH_2 ^{1}J ^{1}H,^{15}N crosspeak can be expressed as

$$\frac{\mathrm{d(NH_2)}}{\mathrm{d}(t_\mathrm{d})} = -\mathrm{NH_2}(t_\mathrm{d})(k_1 + R_1) + k_{-1}\mathrm{NH}'(t_\mathrm{d})$$ Supplementary Equation 2

The decay of NH ^{1}J ^{1}H,^{15}N crosspeak can be expressed as

$$\frac{\mathrm{d(NH)}}{\mathrm{d}(t_\mathrm{d})} = -\mathrm{NH}\,(t_\mathrm{d})(k_{-1} + R_1) + k_1\mathrm{NH_2}'(t_\mathrm{d})$$ Supplementary Equation 3

The build-up of NH' ^{1}J ^{1}H,^{15}N crosspeak can be expressed as

$$\frac{\mathrm{d(NH')}}{\mathrm{d}(t_\mathrm{d})} = -\mathrm{NH}'(t_\mathrm{d})(k_{-1} + R_1) + k_1\mathrm{NH_2}(t_\mathrm{d})$$ Supplementary Equation 4

The build-up of NH_2' ^{1}J ^{1}H,^{15}N crosspeaks can be expressed as

$$\frac{\mathrm{d(NH_2')}}{\mathrm{d}(t_\mathrm{d})} = -\mathrm{NH_2}'(t_\mathrm{d})(k_1 + R_1) + k_{-1}\mathrm{NH}\,(t_\mathrm{d})$$ Supplementary Equation 5

with t_d = delay time, R_1 = autorelaxation rate and k_1 and k_{-1} = rate constants of interconversion.

Integration of Supplementary Equations 2 – 5 gives Supplementary Equations 6 – 9, respectively.

$$\mathrm{NH_2}(t_\mathrm{d}) = \frac{a_0 \mathrm{c}\, \mathrm{e}^{-R_1 t_\mathrm{d}}(\,k_{-1} + k_1 \mathrm{e}^{\mathrm{t_d}(-k_1 - k_{-1})})}{k_1 + k_{-1}}$$ Supplementary Equation 6

$$\mathrm{NH}\ (t_\mathrm{d}) = \frac{q c_0 \mathrm{e}^{-R_1 t_\mathrm{d}}(\,k_1 + k_{-1} \mathrm{e}^{t_d(-k_1 - k_{-1})})}{k_1 + k_{-1}}$$ Supplementary Equation 7

$$\mathrm{NH}'(t_\mathrm{d}) = -\,\frac{q a_0 \mathrm{e}^{-R_1 t_\mathrm{d}} k_1(\,-1 + \mathrm{e}^{t_\mathrm{d}(-k_1 - k_{-1})})}{k_1 + k_{-1}}$$ Supplementary Equation 8

$$\mathrm{NH}_2'(t_\mathrm{d}) = -\frac{c c_0 k_{-1} \mathrm{e}^{-R_1 t_\mathrm{d}}(-1 + \mathrm{e}^{t_\mathrm{d}(-k_1 - k_{-1})})}{k_1 + k_{-1}}$$ Supplementary Equation 9

with t_d = delay time, R_1 = autorelaxation rate, k_1 and k_{-1} = rate constants of interconversion and a_0 , c, and c_0, q = constants.

Variable Ionic Strength

To evaluate the impact of variable ionic strength [^{1}H,^{1}H]-TOCSY and [^{1}H,^{1}H]-NOESY spectra were recorded of metallo duplex samples in the presence of 50 mM, 200 mM, and 500 mM $NaClO_4$. Comparing signal intensities of cytosine (C4) H5/H6 [^{1}H,^{1}H]-TOCSY resonances of major- (AB) and minor species (ab) to their exchange-mediated cross peaks (aB) and (Ab) revealed a decrease in the overall exchange rate of the helices with increasing ionic strength (Supplementary Figure 27).

To investigate changes in the ratio of major- and minor duplex species, the areas of integration of AB, ab, aB, and Ab were determined, scaled to the intensity of the cytosine C1 H5/H6 [^{1}H,^{1}H]-TOCSY correlation, and globally normalized to the spectrum at pH = 9 (Supplementary Figure 32a) to give a sum of integrals = 1. The symmetrical F1/F2 (Supplementary Figure 28) and F2/F1 cross peaks (Supplementary Figure 28) were analyzed separately. The sum of integrals of the major- (AB + aB) and minor species (ab + Ab), and the fraction minor species ((ab + Ab)/(AB + aB + ab + Ab)) revealed minimal change in the ratio of the two species at different ionic strengths (Supplementary Figure 29).

Further evidence for this is found in the [^{1}H,^{1}H]-NOESY spectra of metallo duplex samples in the presence of 50 mM, 200 mM, and 500 mM $NaClO_4$ (Supplementary Figure 30 – 31). Six well resolved NOE

signals of the major- (blue) and minor species (green) were integrated and the area of integration was normalized to a selected reference signal (A12H3' – C13H6, purple, Supplementary Figure 30). The fraction of the minor species was determined for each signal by dividing the area of integration by the sum of the integrals of major- and minor signals. If exchange-mediated signals were present they were included in the sum of integrals (Supplementary Figure 31).

Variable pH

To evaluate the impact of variable pH, [^{1}H,^{1}H]-TOCSY and [^{1}H,^{1}H]-NOESY spectra were recorded of metallo duplex samples at pH = 9, 8, 7, and 6. Comparing signal intensities of cytosine (C4) H5/H6 [^{1}H,^{1}H]-TOCSY resonances of major- (AB) and minor species (ab) to their exchange-mediated cross peaks (aB) and (Ab) revealed a decrease in the overall exchange rate of the helices with increasing pH (Supplementary Figure 32).

The pH dependence on the ratio of major- and minor duplex species was determined as described above for varying ionic strength. Analysis of the [^{1}H,^{1}H]-TOCSY and [^{1}H,^{1}H]-NOESY experiments revealed that the ratio of the two species remained the same from pH 6 – 9 (Supplementary Figure 33 – 35).

Rate Constants of Global Helix Interconversion

To determine rate constants of global interconversion of the duplexes, we measured [^{1}H,^{1}H]-NOESY spectra with various mixing times (t_m) at 25 °C (Supplementary Figures 37 and 38). Selected exchange cross peaks (‘Aa’ and ‘aA’, Supplementary Figures 36a and 37) and exchange-mediated NOE cross peaks (‘Ab’ and ‘aB’, Supplementary Figures 36b and 38) were integrated, normalized to signal intensity at mixing time = 200 ms, and plotted as a function of mixing time (t_m) (Supplementary Figures 39-42). Two NOE signals were also included to the analysis as controls (Supplementary Figure 40). At longer mixing times, a decrease in signal intensity was observed due to auto relaxation (R_1).

The area of integration of diagonal peaks (‘AA’ and ‘aa’) and exchange cross peaks (‘Aa’ and ‘aA’) were determined at various mixing times (t_m) (Supplementary Figure 37, for definition of labels see Supplementary Figure 36a). Under the assumption that auto relaxation $R_{1,A} = R_{1,a}$ and cross relaxation $\sigma = 0$, change in area of integration can be expressed by Supplementary Equations 10 – 12.[17,37] Fit with Supplementary Equation 12 of change in area of integration of exchange cross peaks as a function of mixing time (t_m) furnishes the sum of rate constants $k_1 + k_{-1}$ (Supplementary Figure 39, Supplementary Table 7).

$$\text{diagonal peak 'AA' } (t_{\mathrm{m}}) = \frac{k_{-1}}{(k_1+k_{-1})^2}\, e^{-R_1 t_{\mathrm{m}}}(k_{-1} + k_1 \mathrm{e}^{-(k_1+k_{-1})t_{\mathrm{m}}}) \qquad \text{Supplementary Equation 10}$$

$$\text{diagonal peak 'aa' } (t_{\mathrm{m}}) = \frac{k_1}{(k_1+k_{-1})^2}\, e^{-R_1 t_{\mathrm{m}}}(k_1 + k_{-1} \mathrm{e}^{-(k_1+k_{-1})t_{\mathrm{m}}}) \qquad \text{Supplementary Equation 11}$$

$$\text{exchange cross peak 'Aa' } (t_{\mathrm{m}}) = \text{ exchange cross peak 'aA' } (t_{\mathrm{m}})$$
$$= \frac{k_1 k_{-1}}{(k_1+k_{-1})^2}\, e^{-R_1 t_{\mathrm{m}}}(1 - \mathrm{e}^{-(k_1+k_{-1})t_{\mathrm{m}}}) \qquad \text{Supplementary Equation 12}$$

For signals where the resolution of the diagonal peaks was sufficient for integration, Supplementary Equation 12 can be normalized to the intensity of diagonal peak by 'Aa' (t_{m}) / ('Aa' (t_{m}) + 'aa' (t_{m})) and 'aA' (t_{m}) / ('aA' (t_{m}) + 'AA' (t_{m})) to give the simplified Supplementary Equations 13 and 14, respectively, from which the rate constants k_1 and k_{-1} can be determined (Supplementary Figure 41).[17,37]

$$\text{normalized exchange cross peak} =$$
$$\frac{\text{'Aa'}(t_{\mathrm{m}})}{\text{'Aa'}(t_{\mathrm{m}})+\text{'aa'}(t_{\mathrm{m}})} = \frac{k_{-1}}{k_1+k_{-1}}\left(1 - \mathrm{e}^{-(k_1+k_{-1})t_{\mathrm{m}}}\right) \qquad \text{Supplementary Equation 13}$$

$$\text{normalized exchange cross peak} =$$
$$\frac{\text{'aA'}(t_{\mathrm{m}})}{\text{'aA'}(t_{\mathrm{m}})+\text{'AA'}(t_{\mathrm{m}})} = \frac{k_1}{k_1+k_{-1}}\left(1 - \mathrm{e}^{-(k_1+k_{-1})t_{\mathrm{m}}}\right) \qquad \text{Supplementary Equation 14}$$

The area of integration of NOE cross peaks ('AB' and 'ab') and exchange-mediated NOE cross peaks ('Ab' and 'aB') (for definition of labels see Supplementary Figure 36b) were determined at various mixing times (t_{m}) (Supplementary Figure 38). Under the assumption that auto relaxation $R_{1,\mathrm{A}} = R_{1,\mathrm{a}}$, change in area of integration of NOE cross peaks as a function of mixing time (t_{m}) can be expressed by Supplementary Equation 15 and Supplementary Equation 16.[17,37] Change in area of integration of exchange-mediated cross peaks as a function of mixing time (t_{m}) can be expressed by Supplementary Equation 17.[17,37]

$$\text{NOE cross peak 'AB'}\ (t_\mathrm{m}) = \frac{k_{-1}\mathrm{e}^{-t_\mathrm{m}(k_1+k_{-1}+R_1+\sigma)}\,(-1+\mathrm{e}^{2\sigma t_\mathrm{m}})\,(k_1+k_{-1}\mathrm{e}^{t_\mathrm{m}(k_1+k_{-1})})}{2(k_1+k_{-1})^2}$$ Supplementary Equation 15

$$\text{NOE cross peak 'ab'}\ (t_\mathrm{m}) = \frac{k_{1}\mathrm{e}^{-t_\mathrm{m}(k_1+k_{-1}+R_1+\sigma)}\,(-1+\mathrm{e}^{2\sigma t_\mathrm{m}})\,(k_{-1}+k_{1}\mathrm{e}^{t_\mathrm{m}(k_1+k_{-1})})}{2(k_1+k_{-1})^2}$$ Supplementary Equation 16

$$\text{exchange} - \text{mediated cross peak 'Ab'}\ (\mathrm{t_m}) = \text{exchange} - \text{mediated cross peak 'aB'}\ (\mathrm{t_m})$$

$$= \frac{k_1 k_{-1}\mathrm{e}^{-t_\mathrm{m}(k_1+k_{-1}+R_1+\sigma)}\,(-1+\mathrm{e}^{2\sigma t_\mathrm{m}})\,(-1+\mathrm{e}^{t_\mathrm{m}(k_1+k_{-1})})}{2(k_1+k_{-1})^2}$$ Supplementary Equation 17

By dividing exchange-mediated cross peaks 'Ab' and 'aB' over the sum with their corresponding NOE cross peak gives the simplified Supplementary Equations 18 and 19, respectively, from which the rate constants k_1 and k_{-1} can be determined (Supplementary Figure 42, Supplementary Table 7).[17,37]

$$\text{normalized exchange} - \text{mediated NOE cross peak} =$$

$$\frac{\text{'aB'}(t_\mathrm{m})}{\text{'AB'}(t_\mathrm{m})+\text{'aB'}(t_\mathrm{m})} = \frac{k_1-k_1\,\mathrm{e}^{-t_\mathrm{m}(k_1+k_{-1})}}{k_1+k_{-1}}$$ Supplementary Equation 18

$$\text{normalized exchange} - \text{mediated NOE cross peak} =$$

$$\frac{\text{'Ab'}(t_\mathrm{m})}{\text{'Ab'}(t_\mathrm{m})+\text{'ab'}(t_\mathrm{m})} = \frac{k_{-1}-k_{-1}\,\mathrm{e}^{-(k_1+k_{-1})t_\mathrm{m}}}{k_1+k_{-1}}$$ Supplementary Equation 19

with t_m = mixing time, R_1 = autorelaxation rate and k_1 and k_{-1} = rate constants of interconversion. For definition of labels 'AB', 'ab', 'Ab', and 'aB' see Supplementary Figure 36.

Hg^{II}-Induced Conformational Switching from B- to A-Form

Oligonucleotides were purchased from Sigma-Aldrich as HPLC- and PAGE purified sequences. Oligonucleotide stock solutions were prepared in deionized water and their concentrations were determined by absorbance at 260 nm using a molar extinction coefficient (ε_{260}) calculated using a nearest-neighbor model.[18,19]. Hairpins were prepared by diluting the sequences in aqueous buffer (200 mM $NaClO_4$, 50 mM cacodylic acid (pH = 7.8)) and heating at 95 °C for 5 min, followed by rapid cooling on ice at 0 °C. Duplex DNA ODN^{13} ds was prepared by dissolving the complementary sequences in aqueous buffer (200 mM $NaClO_4$, 50 mM cacodylic acid (pH = 7.8)) heating at 95 °C for 5 min, followed by slow cooling to room temperature over 4 h.

Gel Electrophoreses

Sucrose (40 %, 5 µl) was given to pre-annealed hairpin or duplex DNA (5 µl, 5 pmol) and the solution was loaded onto a 20 % native gel (1.6 V, 55 min). The gel was treated with SYBR gold staining and scanned on a Typhoon FLA 9500. DNA samples measured in the presence of Hg^{II} were incubated with $Hg(ClO_4)_2$ (1.5 equiv relative to mismatch present) for 3 h prior to use.

Neo-BODIPY Binding Assay

Pre-folded DNA hairpin (5 µM) in aqueous buffer (200 mM $NaClO_4$ and 50 mM cacodylic acid (pH = 7.8)) was mixed with $Hg(ClO_4)_2$ (1.5 equiv relative to mismatch present) and incubated at r.t. for 3 h. Neo-BODPIY was added to give a final concentration of 40 nM Neo-BODIPY and 0 – 3 µM DNA hairpin. The samples were incubated at r.t. for 30 min (ODN^{13}, Figure 7a) or 1 h (ODN^{4} – ODN^{13}, Table 2) prior to measurement. Fluorescence anisotropy values were determined by measuring fluorescence polarization at λ_{em} = 515 nm (λ_{ex} = 480 nm). Fluorescence polarization was measured in three independent trials at 25 °C using a Molecular Devices Spectra spectrofluorophotometer in 384-well plates.

A → B Helical Switching by Unlabeled Neomycin B

Pre-folded DNA hairpin (1 μM) in aqueous buffer (200 mM $NaClO_4$ and 50 mM cacodylic acid (pH 7.8)) was mixed with $Hg(ClO_4)_2$ (22.5 μM, 1.5 equiv relative to mismatch present) and incubated at r.t. for 3 h. Neo-BODIPY (66.7 nM) was added and incubated at r.t. for 60 min. Unlabeled neomycin B trisulfate was added to the samples to give a final concentration of 40 nM Neo-BODIPY, 0.6 μM ODN[13], 13.5 μM Hg^{II} and 0 – 50 μM unlabeled neomycin B and the mixture was incubated at r.t. for 60 min. Anisotropy was recorded at 25 °C using a Molecular Devices Spectra spectrofluorophotometer in a 384-well plate. Fluorescence anisotropy was recorded in triplicate by fluorescence polarization measurement (λ_{ex} = 480 nm, λ_{em} = 515 nm).

A → B Helical Switching by *N*-Acetylcysteine

Pre-folded DNA hairpin (1 μM) in aqueous buffer (200 mM $NaClO_4$ and 50 mM cacodylic acid (pH 7.8)) was mixed with $Hg(ClO_4)_2$ (22.5 μM, 1.5 equiv relative to mismatch present) and incubated at r.t. for 3 h. Neo-BODIPY (66.7 nM) was added and incubated at r.t. for 60 min. *N*-acetylcysteine (0 – 2 equiv relative to mismatch present) was added to the sample to give a final concentration of 40 nM Neo-BODIPY, 0.6 μM ODN[13], 13.5 μM Hg^{II} and 0 – 18 μM *N*-acetylcysteine and the mixture was incubated at r.t. for 60 min. Anisotropy was recorded at 25 °C using a Molecular Devices Spectra spectrofluorophotometer in a 384-well plate. Fluorescence anisotropy was recorded in triplicate by fluorescence polarization measurement (λ_{ex} = 480 nm, λ_{em} = 515 nm).

Circular Dichroism Spectroscopy of DNA Repeat Sequences

Circular dichroism spectra of pre-folded DNA hairpins (1 μM) were measured from 200 nm to 350 nm at 25 °C with a 2 nm band width with 0.1 nm steps at a scanning rate of 20 nm min^{-1} in 1 mm path length thermo-controlled strain-free quartz cuvette on a JASCO J-715 spectrometer. CD spectra were recorded as an average of three measurements, each with three scans. CD spectra of hairpin DNA measured in the presence of Hg^{II} were incubated with 1.5 equiv (relative to mismatch present) of $Hg(ClO_4)_2$ for 3 h prior to use.

Hg^{II}–ODN^{13} Binding and Reversibility by *N*-Acetylcysteine

Aliquots of $Hg(ClO_4)_2$ (0 – 1.5 equiv relative to mismatch) were added to pre-folded ODN^{13} hairpin (1 μM) in aqueous buffer (200 mM $NaClO_4$ and 50 mM cacodylic acid (pH = 7.8). CD spectra were recorded as described above after 10 min incubation time. After complete B- to A-form helical transition (after addition of 1.5 equiv of Hg^{II} relative to mismatch) aliquots of *N*-acetylcysteine (0 – 1.5 equiv relative to mismatch) were added and CD spectra were recorded after 10 min of incubation.

Hg^{II} –ODN^{13} ds Binding and Reversibility by *N*-Acetylcysteine

Aliquots of $Hg(ClO_4)_2$ (0 – 1.0 equiv relative to mismatch) were added to pre-folded ODN^{13} ds (3 μM) in aqueous buffer (200 mM $NaClO_4$ and 50 mM cacodylic acid (pH = 7.8). CD spectra were recorded as described above after 10 min incubation time. After complete B- to A-form helical transition (after addition of 1.0 equiv of Hg^{II} relative to mismatch) aliquots of *N*-acetylcysteine (0 – 1.0 equiv relative to mismatch) were added and CD spectra were recorded after 10 min of incubation.

To investigate the reversibility of A/B-form helical switching, various equiv Hg^{II} (equiv relative to mismatch) and *N*-acetylcysteine (equiv relative to mismatch) were added in alternating order to pre-folded ODN^{13} ds (1 μM) in aqueous buffer (200 mM $NaClO_4$ and 50 mM cacodylic acid (pH = 7.8). CD spectra were recorded after 30 sec of incubation and changes in molar ellipticity were monitored at 260 nm and 280 nm.

Supplementary References

1. Schmidt, O. P., Benz, A. S., Mata, G., Luedtke, N. W. Hg^{II} binds to C–T mismatches with high affinity. *Nucleic Acids Res.* **46**, 6470–6479 (2018).
2. Hosur, R. V et al. Solution structure of d-GAATTCGAATTC by 2D NMR: A new approach to determination of sugar geometries in DNA segments. *FEBS Lett.* **205**, 71–76 (1986).
3. Davies, D. B. Conformations of nucleosides and nucleotides. *Prog. Nucl. Magn. Reson. Spectroscopy* **12**, 135–225 (1978).
4. Widmer, H. & Wuthrich, K. Simulated two-dimensional NMR cross-peak fine structures for ^1H spin systems in polypeptides and polydeoxynucleotides. *J. Magn. Reson.* **74**, 316–336 (1987).

5. Altona, C. & Sundaralingam, M. Conformational analysis of the sugar ring in nucleosides and nucleotides. a new description using the concept of pseudorotation. *J. Am. Chem. Soc.* **94**, 8205–8212 (1972).

6. The PyMOL Molecular Graphics System, Version 2.0.7, Schrödinger, LLC. The PyMOL Molecular Graphics System, Version 2.0.7, Schrödinger, LLC.

7. Lavery, R., Moakher, M., Maddocks, J. H., Petkeviciute, D. & Zakrzewska, K. Conformational analysis of nucleic acids revisited: Curves +. *Nucleic Acids Res.* **37**, 5917–5929 (2009).

8. Roberts, G. C. K. *NMR of Macromolecules*. (Oxford University Press, New York, 1993).

9. Frederick, C. A. et al. Molecular structure of an A-DNA decamer d(ACCGGCCGGT). *Eur. J. Biochem.* **181**, 295–307 (1989).

10. Drew, H. R. et al. Structure of a B-DNA dodecamer : Conformation and dynamics. *Proc. Natl. Acad. Sci.* **78**, 2179–2183 (1981).

11. Lu, X.-J. & Olson, W. K. 3DNA: a software package for the analysis, rebuilding and visualization of three-dimensional nucleic acid structures. *Nucleic Acids Res.* **31**, 5108–5121 (2003).

12. Lu, X.-J. & Olson, W. K. 3DNA: a versatile, integrated software system for the analysis, rebuilding and visualization of three-dimensional nucleic-acid structures. *Nat. Protoc.* **3**, 1213–1227 (2008).

13. Olson, W. K. et al. A standard reference frame for the description of nucleic acid base-pair geometry. *J. Mol. Biol.* **313**, 229–237 (2001).

14. Lu, X.-J., Shakked, Z., Olson, W. K. A-form conformational motifs in ligand-bound DNA structures. *J. Mol. Biol.* **300**, 819–840 (2000).

15. S. Arnott, P.J. Campbell-Smith & R. Chandrasekaran. In *Handbook of Biochemistry and Molecular Biology*, 3rd ed. Nucleic Acids--Volume II, G.P. Fasman, Ed. Cleveland: CRC Press, (1976).

16. Pasi, M. et al. Analyzing DNA curvature and its impact on the ionic environment: application to molecular dynamics simulations of minicircles. *Nucleic Acids Res.* **45**, 4269–4277 (2017).

17. Choe, B., Cook, G. W., Rama Krishna, N., Effect of slow conformational exchange in 2D NOESY spectra. *J. Magn. Res.*, **94**, 387–393 (1991).

18. Cantor, C. R., Warshaw, M. M. & Shapiro, H. Oligonucleotide Interactions III. Circular dichroism studies of the conformation of deoxyoligonucleotides. *Biopolymers* **9**, 1059–1077 (1970).

19. Borer, P. N. Optical properties of nucleic acids, absorption and circular dichroism spectra. In *Handbook of Biochemistry and Molecular Biology: Nucleic Acids* (ed. Fasman, G. D.) 589–595 (CRC Press, 1975).

20. Schmidt, O. P., Mata, G., Luedtke, N. W. Fluorescent base analogue reveals T-Hg^{II}-T base pairs have high kinetic stabilities that perturb DNA metabolism. *J. Am. Chem. Soc.* **138**, 14733–14739 (2016).

21. Wishart, D. S. et al. ^{1}H, ^{13}C, ^{15}N chemical shift referencing in biomolecular NMR. *J. Biomol. NMR* **6,** 135–140 (1995).

22. Bax, A. & Lerner, L. Measurement of ^{1}H-^{1}H coupling constants in DNA fragments by 2D NMR. *J. Magn. Reson.* **79**, 429–438 (1988).

23. Güntert, P., Mumenthaler, C. & Wüthrich, K. Torsion angle dynamics for NMR structure calculation with the new program DYANA. *J. Mol. Biol.* **273**, 283–298 (1997).

24. Schwieters, C. D., Kuszewski, J. J., Tjandra, N. & Clore, G. M. The Xplor-NIH NMR molecular structure determination package. *J. Magn. Reson.* **160**, 65–73 (2003).

25. Schwieters, C. D., Kuszewski, J. J. & Clore, G. M. Using Xplor – NIH for NMR molecular structure determination. *Prog. Nucl. Magn. Reson. Spectroscopy* **48**, 47–62 (2006).

26. Tjandra, N.; Tate, S.-I.; Ono, A.; Kainosho, M.; Bax, A., The NMR structure of a DNA dodecamer in an aqueous dilute liquid crystalline phase. *J. Am. Chem. Soc.* **122**, 6190–6200 (2000).

27. Varani, G., Aboul-ela, F. & Allain, F. H. NMR investigation of RNA structure. *Prog. Nucl. Magn. Reson. Spectroscopy* **29**, 51–127 (1996).

28. Schneider, B.; Neidle, S.; Berman, H. M., Conformations of the sugar-phosphate backbone in helical DNA crystal structures. *Biopolymers,* **42**, 113–124 (1997).

29. Sklenar, V.; Bax, A., Measurement of ^{1}H-^{31}P NMR coupling constants in double-stranded DNA fragments. *J. Am. Chem. Soc*. **109**, 7525–7526 (1987).

30. Kondo, J. et al. Crystal structure of metallo DNA duplex containing consecutive Watson-Crick-like T-Hg(II)-T base pairs. *Angew. Chem. Int. Ed. Engl.* **53**, 2385–2388 (2014).

31. Yamaguchi, H. et al. The structure of metallo-DNA with consecutive thymine-HgII-thymine base pairs explains positive entropy for the metallo base pair formation. *Nucleic Acids Res.* **42**, 4094–4099 (2014).

32. Kosturko, L. D., Folzer, C. & Stewart, R. F. The crystal and molecular structure of a 2 : 1 complex of 1-methylthymine-mercury(II). *Biochemistry* **13**, 3949–3952 (1974).

33. Menzer, S., Sabat, M. & Lippert, B. Ag(I) modified base pair involving complementary (G,C) [guanine, cytosine] and noncomplementary (A,C) [adenine, cytosine] nucleobases. On the possible structural role of aqua ligands in metal-modified nucleobase pairs. *J. Am. Chem. Soc.* **114**, 4644–4649 (1992).

34. Liu, H. et al. Flexibility and stabilization of HgII-mediated C : T and T : T base pairs in DNA duplex. *Nucleic Acids Res.* **45**, 2910–2918 (2017).

35. Koradi, R., Billeter, M. & Wüthrich, K. MOLMOL: A program for display and analysis of macromolecular structures. *J. Mol. Graph.* **14**, 51–55 (1996).

36. Farrow, N. A., Zhang, O., Forman-Kay, J. D., Kay, L. E., A heteronuclear correlation experiment for simultaneous determination of 15N longitudinal decay and chemical exchange rates of systems in slow equilibrium. *J. Biomol. NMR,* **4**, 727–734 (1994).

37. Jeener, J., Meier, B. H., Bachmann, P., Ernst, R. R., Investigation of exchange processes by two-dimensional NMR spectroscopy. *J. Chem. Phys.,* **71**, 4546–4553 (1979).
