# Supplementary figures and images for: Concerted dynamics of metallo-base pairs in an A/B-form helical transition
Source: Nat Commun. 2019 Oct 23;10:4818. doi: 10.1038/s41467-019-12440-x (PMC6811676; doi:10.1038/s41467-019-12440-x)

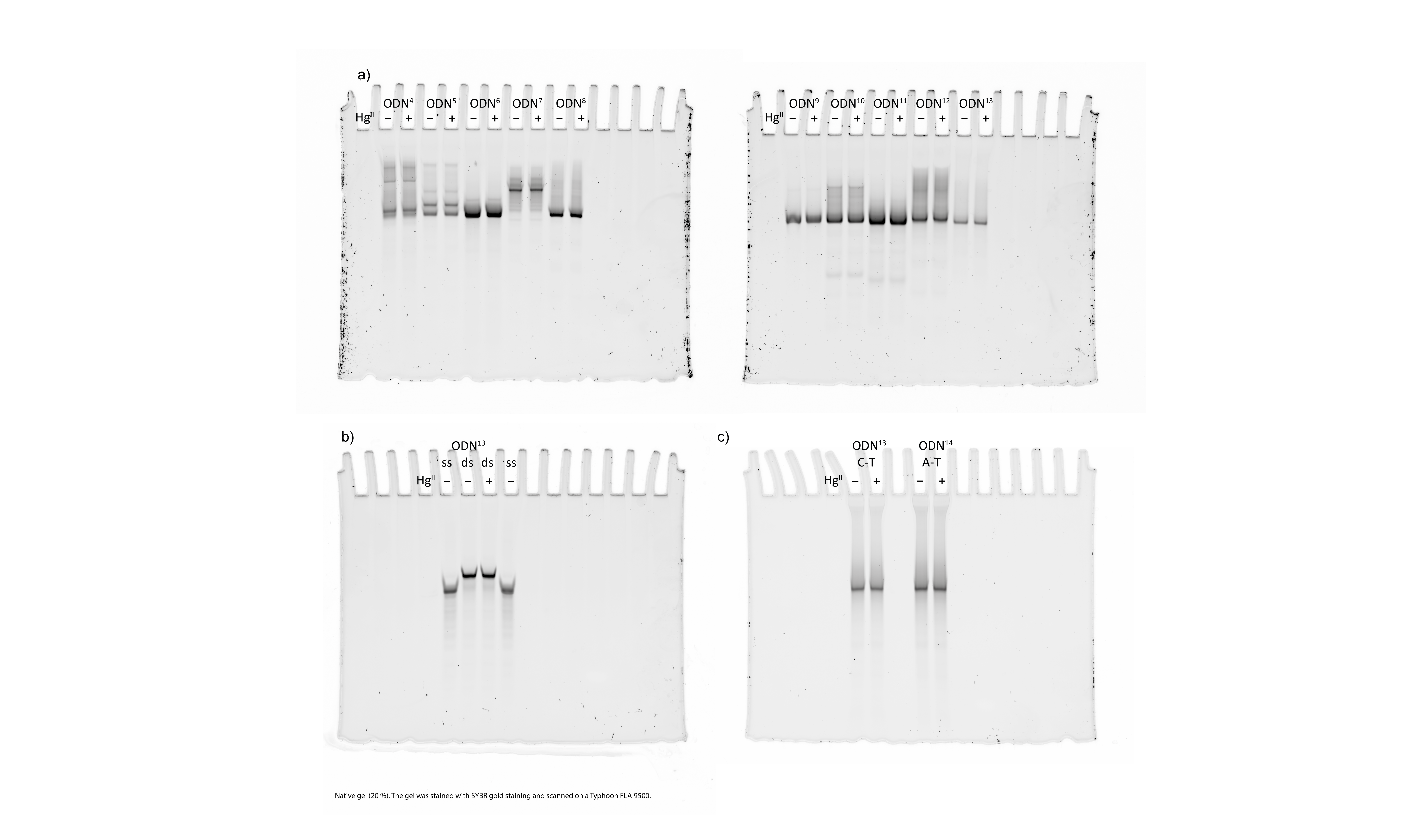

Supplement: Supplementary file 3 — Source Data [file 41467_2019_12440_MOESM3_ESM.zip › New_Sourcedata/Supplementary_Figure43.png]
